# DTNI: a novel toxicogenomics data analysis tool for identifying the molecular mechanisms underlying the adverse effects of toxic compounds

**DOI:** 10.1007/s00204-016-1922-5

**Published:** 2016-12-28

**Authors:** Diana M. Hendrickx, Terezinha Souza, Danyel G. J. Jennen, Jos C. S. Kleinjans

**Affiliations:** 1 Department of Toxicogenomics, GROW-School for Oncology and Developmental Biology, Maastricht University, Universiteitssingel 40, 6229 ER Maastricht, The Netherlands; 2 P.O. Box 616, 6200 MD Maastricht, The Netherlands

**Keywords:** Time series, Dose–response, Gene regulatory network inference, Mode of action, Molecular initiating event, Key event

## Abstract

Unravelling gene regulatory networks (GRNs) influenced by chemicals is a major challenge in systems toxicology. Because toxicant-induced GRNs evolve over time and dose, the analysis of global gene expression data measured at multiple time points and doses will provide insight in the adverse effects of compounds. Therefore, there is a need for mathematical methods for GRN identification from time-over-dose-dependent data. One of the current approaches for GRN inference is Time Series Network Identification (TSNI). TSNI is based on ordinary differential equations (ODE), describing the time evolution of the expression of each gene, which is assumed to be dependent on the expression of other genes and an external perturbation (i.e. chemical exposure). Here, we present Dose-Time Network Identification (DTNI), a method extending TSNI by including ODE describing how the expression of each gene evolves with dose, which is supposed to depend on the expression of other genes and the exposure time. We also adapted TSNI in order to enable inclusion of time-over-dose-dependent data from multiple compounds. Here, we show that DTNI outperforms TSNI in inferring a toxicant-induced GRN. Moreover, we show that DTNI is a suitable method to infer a GRN dose- and time-dependently induced by a group of compounds influencing a common biological process. Applying DTNI on experimental data from TG-GATEs, we demonstrate that DTNI provides in-depth information on the mode of action of compounds, in particular key events and potential molecular initiating events. Furthermore, DTNI also discloses several unknown interactions which have to be verified experimentally.

**Electronic supplementary material:**

The online version of this article (doi:10.1007/s00204-016-1922-5) contains supplementary material, which is available to authorized users.

## Introduction

A dedicated and robust analysis of human in vitro toxicogenomics data by means of computational methods is crucial for the development of alternatives to animal testing (Goh et al. 2015). In this context, studying the influence of toxic compounds on gene regulatory networks (GRN) is important for the identification of their adverse effects on the cellular level (Gautier et al. 2013). However, understanding how chemical compounds influence GRN is challenging, because it requires the identification of causal events (Liebler and Guengerich 2005). Thus, we need to reliably capture the dynamics of toxicogenomics responses. Time series analysis allows for detecting similar patterns across multiple time scales, while static data analysis through searching for associations only identifies simultaneously occurring patterns (Cavill et al. 2013; Hendrickx et al. 2015). Therefore, analyses of toxicogenomics responses over time are required to infer hypotheses on the order of causal events (Bar-Joseph et al. 2012). Apart from the time, the dose is also an important factor for identifying mechanisms of toxicity (Allen et al. 2014). However, time-over-dose integration approaches are currently lacking.

Open TG-GATEs (http://toxico.nibiohn.go.jp/) (Igarashi et al. 2015), containing data from 158 hepatotoxic compounds generated from human primary hepatocytes at several time points and doses, provide the opportunity to explore time-over-dose integration methods in human in vitro data. However, previous analyses of the TG-GATEs data were mainly restricted to separate analyses per time point and dose (Chung et al. 2015; Grinberg et al. 2014; Kanki et al. 2016; Zhang et al. 2014). Chung et al. (2015) explored the probability of relationships between treatment, drug and time or dose. Grinberg et al. (2014) focussed on distinguishing stereotypic and compound-specific responses of gene expression by determining differentially expressed genes (DEGs). Kanki et al. (2016) applied multivariate analysis (principal component analysis) to study the mode of action of non-genotoxic hepatocarcinogens. Zhang et al. (2014) identified an early response network for cytotoxicity based on DEGs at the earliest time point (2 h) and prior knowledge on their interactions. However, these studies do not address the identification of novel gene interactions and do not focus on time-over-dose integration.

Previously developed methods for inferring GRNs can be roughly divided into five types of approaches and combinations thereof: (1) linear regression; (2) pairwise scores; (3) nonlinear regression; (4) ordinary differential equations (ODE); and (5) Bayesian networks (Hill et al. 2016). However, none of these approaches takes into account that gene expression evolves with both time and dose.

Here, we have preferred to extend the ODE approach to GRN inference, because methods based on ODE are especially suitable for time series and aim at identifying quantitative dynamical gene regulatory interactions (Le Novere 2015). We present Dose-Time Network Identification (DTNI), a novel method for GRN inference based on ODE and time-over-dose integration. DTNI extends existing ODE methods for GRN inference by including ODE describing the evolution of gene expression with the dose, while traditional ODE methods only describe the evolution of gene expression over time. We show that DTNI can be used to study a group of chemicals influencing a pathway or biological process of interest. Within this pathway or biological process, DTNI determines the subnetwork influenced by this group of chemicals across all time points and doses. Analysis of subnetworks inferred by DTNI will elucidate information about the molecular mechanisms underlying the adverse effects of toxic compounds.

First, we will study the performance of DTNI using simulated data on a pre-defined network, namely the IkB-NF-kB signalling module. A simulation study aims at assessing how well a known network can be predicted by a network inference method, which cannot be accomplished by analysing experimental data, because for real data, biological knowledge is uncertain and incomplete (Mendes et al. 2005). Furthermore, simulations have the purpose to study how the performance of a network inference method would improve when sampling at a higher resolution that cannot be achieved with current experiments. This is important for future experimental design. Finally, simulations intend to determine the influence of a known level of noise (variation due to biological and/or technical factors), while for real data, only an estimation of the noise levels can be made.

Next, we will apply DTNI on experimental data on human hepatocytes from Open TG-GATEs in order to study two important pathways related to drug-induced liver injury (DILI): the nuclear factor-kappa B (NF-kB) signalling pathway and the nuclear factor (erythroid-derived 2)-like 2 (NRF2) pathway.

We furthermore demonstrate that heat maps of starting nodes (nodes with only outgoing edges) in networks inferred with DTNI at the earliest time point can be used to infer potential molecular initiating events (MIEs) within the adverse outcome pathway (AOP) framework (Ankley et al. 2010).

## Materials and methods

### Time series network identification (TSNI)

TSNI (Bansal et al. 2006) is a network inference algorithm based on (linear) ODEs. The system of ODEs relates gene expression changes over time to each other and to a perturbation (in this study: exposure to a certain dose of a toxic compound). The method can include both constant and transient perturbations. Parameters in the ODEs are estimated by means of regression techniques and represent the interaction strengths between genes. In transcriptomics data, the number of genes is mostly much larger than the number of measurements, and as a consequence, the ODEs do not have a unique solution. Therefore, the ODE approach is combined with an interpolation step to calculate extra time points, and a dimension reduction step (comparable with principal component analysis). Parameters are first estimated in the reduced dimension space, and the obtained solution is then transformed to parameters for the original system of ODE. In this way, identifiability problems are addressed and we obtain a unique set of parameters (interaction strengths) for the ODE.

TSNI has some shortcomings, which hamper application of this method to toxicogenomics data. First, TSNI requires that data are sampled at equal time intervals, which is mostly not the case in toxicogenomics. Second, the response to a compound not only evolves over time, but also over dose. TSNI includes dependency of gene expression time courses on the dose, but does not include ODEs that describe how gene expression evolves with dose given the time of exposure. Third, a threshold on the interaction strength determines which edges are kept in the network and which are not. These thresholds are quite arbitrary.

Hence, we developed a novel method, named DTNI that addresses these issues.

### Dose-Time Network Identification (DTNI)

DTNI extends TSNI by adding extra ODE describing the evolution of gene expression over dose, assuming dependence on expression of the other genes and the time of exposure. As in TSNI, a dimension reduction step is applied to avoid identifiability problems. Furthermore, the interpolation step is adapted, in order to enable that a time series with equal sampling intervals is estimated in case of unequal sampling intervals. Moreover, an interpolation step for interpolating dose series is added, so that extra dose points can be estimated and unequal dose intervals can be handled. Significance is calculated for each interaction by an adapted permutation test. Traditional permutation testing is not suitable for data obtained at multiple time points and multiple doses, because permutation testing assumes independency between the measurements, and does not take into account time and dose dependencies. While the data depend on each other, this is not necessarily the case for the residuals of the ODE model (i.e. the difference between the real data and the data estimated from the ODE model). Therefore, we can permute the residuals instead of the data and add the permuted residuals to the data estimated from the ODE model (Lee and Braun 2012). We performed 1000 permutations of the residuals and calculated a *p* value for each interaction, in the same way as for a traditional permutation test. In this way, we could determine significance of the inferred interactions, instead of relying on arbitrary thresholds on the interaction strength. The method was implemented within MATLAB (MATLAB 2014). A detailed description of the algorithm is provided in Supplementary Material 2. The MATLAB code is available in Supplementary Material 3.

### Leave-one-out cross-validation (LOOCV)

When determining the network affected by a group of compounds, leave-one-out cross-validation (LOOCV) was performed by leaving out the data from one compound. This was repeated for each compound in the data set to create as many LOOCV data sets as there were compounds. DTNI was performed on each LOOCV data set. Interactions found by applying DTNI to the full data set that were not found for all LOOCV data sets were removed from the network.

### Performance of DTNI

To assess the performance of DTNI, a simulation study was conducted. Simulations were performed using the free software COPASI (http://copasi.org/), a bioinformatics tool able to simulate time series data from an ODE model by solving its equations over a time interval of interest (Hoops et al. 2006). A kinetic ODE model on the IkB-NF-kB signalling module (Hoffmann et al. 2002), which is part of the NF-kB pathway, was retrieved from the BioModels Database (Li et al. 2010) (BioModels ID: BIOMD0000000140). The model is based on mass action kinetics, a detailed overview of all reactions, and ODE can be retrieved from the BioModels Database. Additional reactions for drugs acting on this pathway were added by adjusting the system of ODE as described in Sung and Simon (2004). The interaction network consists of 22 nodes and 92 edges. The list of drug reactions in the model and the interaction network is provided in Table S3-1 and Figure S3-1.

Data were simulated to compare the performance of DTNI with TSNI. Furthermore, the influence of the number of time points, number of doses, number of compounds, noise and LOOCV was assessed. Table S3-2 gives an overview of the simulations performed in this study.

Time series were simulated by solving the system of ODE with COPASI. Initial values of the variables in the model for all simulations are shown in Table S3-3. The doses of the compounds for each simulation are given in Table S3-4. An overview of the time points included in the simulated data is presented in Table S3-5. Control data were simulated by setting the concentration value of all compounds to zero. Log2 ratios were calculated as input voor TSNI and DTNI. For the simulations with three time points and three doses, the time points and the ratio between the doses were chosen in such a way that they reach the same values as in the TG-GATEs data, in order to mimic real experimental data as good as possible.

For the simulation study, we assess how well the network of the IkB-NF-kB signalling module extracted from the BioModels Database can be identified by DTNI. Therefore, we define (1) a direct gene–gene interaction: two genes interacting without the intervention of other genes; (2) an indirect gene–gene interaction: two genes interacting through the intervention of one or more intermediate genes; (3) a true-positive interaction (TP): a correctly identified direct interaction; (4) a false-positive interaction (FP): an incorrectly identified direct interaction (the interaction in the network extracted from BioModels is indirect, or there is no interaction); (5) a true negative (true absent interaction) (TN): absence of a direct interaction is correctly identified; and (6) a false negative (false absent interaction) (FN): absence of a direct interaction is incorrectly identified (there is a direct interaction in the network extracted from BioModels).

The following performance measures were used to check out the inferred networks: (1) sensitivity (true-positive rate (TPR)): the proportion of directly interacting gene pairs that are inferred as such; (2) specificity (true-negative rate (TNR)): the proportion of not directly interacting gene pairs that are inferred as such; (3) area under the receiver operator characteristics curve (AUROC): graph of TPR against the false-positive rate (FPR = 1 − TNR), a larger AUROC means a better prediction of the toxicant(s)-induced GRN; (4) geometric mean score (geometric mean of the sensitivity and the specificity), a larger geometric mean score means a better prediction of the toxicant(s)-induced GRN; and (5) positive predictive value (PPV): the proportion of direct interactions predicted by network inference that are true direct interactions.

Apart from conducting a simulation study, we also validated DTNI in real experiments with compounds that were known to have an effect on the peroxisome proliferator-activated receptor (PPAR) signalling pathway, among other clofibrate. A detailed description of the experiments and their analysis is given in Supplementary Material 4 (paragraph “Validation on real experiments—PPAR signalling pathway”).

### Approach to analyse experimental data

**Fig. 1:**
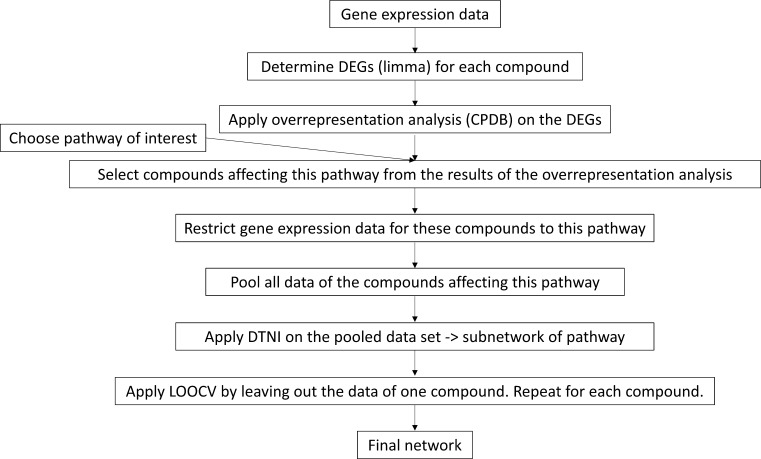
Overview of the approach to analyse experimental data with DTNI

The approach to analyse experimental data with DTNI is outlined in Fig. 1. Because experimental data consist of thousands of variables and only relatively few samples, the number of genes considered for further analysis had to be reduced. This was achieved by restricting the analysis to a group of genes involved in a pathway or biological process of particular interest. Compounds influencing this pathway were selected by determining DEGs (see below) for all compounds in the data set (in this study TG-GATEs) and analysing the lists of DEGs by means of over-representation analysis in ConsensusPathDB (CPDB) (http://consensuspathdb.org/) (Kamburov et al. 2013) to obtain a lists of pathways affected by the compounds. The data of the compounds related to the pathway of interest are pooled and further analysed by applying DTNI (using only data of the genes involved in this pathway) to obtain a single network. Then, LOOCV was conducted by leaving out the data of one compound. The connections of the resulting interaction network were further validated by means of CPDB-induced network modules analysis and were classified using the following definitions: (1) a true-positive interaction (TP): a gene pair linked in CPDB by direct interaction or via biological variables not in the analysed gene list (pathway) that is predicted by DTNI as a directly interacting gene pair; (2) a false-positive interaction (FP): a gene pair only linked in CPDB via genes in the analysed gene list (pathway) that is predicted by DTNI as a directly interacting gene pair; and (3) a novel interaction: a gene pair not linked in CPDB that is predicted by DTNI as a directly interacting gene pair.

Subnetworks within the inferred networks were determined using NetworkAnalyzer/Subnetwork Creation in Cytoscape (version 3.2.1.) (Su et al. 2014). The functionality of the individual genes in the inferred networks was extracted from the GeneCards database (http://www.genecards.org). GeneCards (Safran et al. 2010) provides information on the function of genes from Genatlas (Frezal 1998) and UniProtKB (The_UniProt_Consortium 2015). Over-representation analysis in CPDB was performed on the list of nodes of the inferred networks and their subnetworks to obtain an overview of pathways related to the networks. The pathways in the list are related to effects on the cellular level involved in liver toxicity (e.g. apoptosis, inflammation). By analysing the function of the individual genes and the pathways extracted from CPDB, potential key events related to adverse outcomes of the group of compounds analysed can be detected. Furthermore, a heat map of the starting nodes (nodes with only outgoing edges) in the inferred network at the earliest time point (2 h) was created in order to infer potential MIEs.

Novel interactions not available from CPDB were further analysed for putative functional relationships by consulting the databases Biograph, STRING and CTD. We inferred putative functional relationships from known interactions (genetic interactions, protein interactions, biochemical reactions) by automated hypothesis generation in Biograph (http://biograph.be/) (Liekens et al. 2011), a database integrating 21 publicly available sources containing biomedical relations. Furthermore, we identified functional relationships between genes in STRING (http://string-db.org/) (Jensen et al. 2009), a database integrating experimental repositories, computationally predicted interactions and text mining. Finally, interactions of the gene pairs with the studied compounds were retrieved from the Comparative Toxicogenomics Database (CTD) (http://ctdbase.org/) (Davis et al. 2015). When a gene regulatory relationship was found in Biograph, we further investigated whether the genes have a common gene function using GeneCards database.

To determine whether the resulting network was specific for compounds affecting the studied pathway, the DTNI analysis was repeated for an arbitrary group of compounds in TG-GATEs not affecting this pathway.

### TG-GATEs data

Microarray data were retrieved from TG-GATEs (http://toxico.nibiohn.go.jp/) (Igarashi et al. 2015). This database comprises, among other measurements, a large in vitro data set from human hepatocytes exposed to 158 chemicals of pharmaceutical interest, of which many are known to cause DILI (Chen et al. 2011, 2013). Data were measured for two biological replicates at up to three doses (low, middle and high, with a ratio 1:5:25) across three time points (2, 8 and 24 h) and include also time-matched controls. The highest dose is the level corresponding to a 80–90% survival ratio, except for chemicals poorly dissolving in the vehicle, where the highest dose is the maximum solubility of the chemical (Igarashi et al. 2015).

#### Pre-processing and normalization

Prior to analysis, compounds were pre-selected based on full availability of data from doses, time points and both replicates. Raw files were then pre-processed using the R package affy (Gautier et al. 2004), in which quality control, background correction and robust multiarray averaging normalization (RMA) were performed separately for all chemical sets (containing 24 arrays each). Probe reannotation was performed using a customCDF annotation (version 19) with Entrez gene identifiers for Affymetrix GeneChip Human Genome U133 Plus 2.0 arrays obtained from BrainArray (http://brainarray.mbni.med.umich.edu/brainarray/).

#### Differentially expressed genes

The R/Bioconductor package LIMMA (Ritchie et al. 2015) was used to perform a moderated t-test for each chemical set independently to compare mean intensities across all doses to those from time-matched controls. For the two biological replicates in the data set, treatments were paired to controls. DEGs were further selected by applying the following criteria: (1) absolute fold change of 1.5 and (2) adjusted *p* value (Benjamini–Hochberg) ≤0.05.

#### Pathway analysis

From the pathway lists obtained by means of over-representation analysis in ConsensusPathDB, pathways with adjusted *p* value (*q* value) lower than 0.05 were considered significant.

#### Selection of pathways

We selected the NF-kB and the NRF2 pathways as examples to evaluate DTNI, because these pathways are very well established in the literature, in comparison with other DILI-related pathways.

#### Example 1: NF-kB pathway

The data of the compounds influencing the NF-kB pathway were analysed by applying DTNI. The NF-kB pathway was selected a priori because it is known to play an important role in toxicant-induced liver injury (He and Karin 2011). Activation of this pathway has been related to hepatic inflammation, fibrosis, development of hepatocellular carcinoma, cholestasis, cirrhosis, apoptosis, necrosis and hepatic steatosis (Elsharkawy and Mann 2007; He and Karin 2011).

The NF-kB pathway was extracted from KEGG (http://www.genome.jp/kegg/) (Kanehisa et al. 2016) and consisted of 91 genes, of which 87 were present in TG-GATEs.

#### Example 2: NRF2 pathway

We analysed the data of the compounds influencing the NRF2 pathway. The NRF2 pathway is one of the main pathways protecting the liver from DILI, in particular caused by oxidative and electrophilic stress (Klaassen and Reisman 2010). Activation of NRF2 prevents liver inflammation, necrosis, cholestasis and fibrosis (Bataille and Manautou 2012; Klaassen and Reisman 2010; Tang et al. 2014).

The NRF2 pathway was extracted from Wikipathways (http://www.wikipathways.org) (Kutmon et al. 2016) and consisted of 150 genes, of which 125 were present in TG-GATEs.

## Results

### Performance of DTNI

Figure S3-2 compares DTNI with TSNI for simulated data of a single compound with three time points (2, 8, 24 h), three doses (low, middle and high) and time-matched controls (dose = 0). ROC curves were produced by varying the *p* value threshold and calculating true-positive rates and false-positive rates. The resulting ROC curves show that DTNI outperforms TSNI in predicting a toxicant-induced network (AUROC for DTNI ~5% larger than for TSNI).

Figures S3-3a and S3-3b show that increasing the number of time points or the number of doses leads to a better prediction of a toxicant-induced network (increase in geometric mean score in Figures S3-3a and S3-3b, demonstrating better sensitivity/specificity). However, the curves of Figures S3-3a and S3-3b only slightly increase after having 72 time points and 14 doses, respectively. Figure S3-3c shows that the prediction of a toxicant-induced network increases with the number of compounds (increase in geometric mean score in Figure S3-3c).

The ROC curves in Figure S3-3d show that noise (variation due to biological and/or technical factors) significantly influences the performance of DTNI. However, as to be expected, having replicates reduces the influence of noise.

Figure S3-4 shows that applying LOOCV significantly increases the proportion of direct interactions predicted by DTNI that are true direct interactions (increased PPV in Figure S3-4).

For the real experiments of compounds affecting the PPAR signalling pathway, geometric mean scores of ~0.69 (69%) were obtained (see Table S3-6). Furthermore, the inferred network for clofibrate contains key genes for compound reactions, but also many other genes, of which most of them have already been related to clofibrate exposure in previous studies (see Table S3-7).

### TG-GATEs data

#### Example 1: NF-kB pathway

Four compounds could be related to the NF-kB pathway upon over-representation analysis of the DEGs: acetaminophen, carbon tetrachloride, interleukin-1 and interleukin-6. The interaction network inferred by applying DTNI on this group of compounds consists of 25 nodes, connected by 21 edges (Figure S4-1), of which 14 connections (66.7%) are true-positive interactions, only 2 connections are false-positive interactions (9.5%) and 5 connections are unknown (23.8%). Details on the edges are presented in Table S4-1. Table S4-2 presents the list of pathways related to the inferred network. The network can be divided into 6 subnetworks (Figure S4-1 and Table S4-3). The inferred network and its subnetworks are related to apoptotic pathways (a.o. TNF and FAS signalling) and inflammatory response pathways (cytokine signalling, a.o. interleukin and TNF signalling; TLR signalling).

Within the complete network of 25 nodes, 12 were starting nodes (with only outgoing edges). Figure S4-2 presents a heat map of these starting nodes at the earliest time point (2 h). TLR4, which plays a role in the inflammatory response (Broering et al. 2011), seems to be the most affected by 3 out of 4 compounds at the earliest time point. Furthermore, the other genes in the inferred network are involved in DNA recombination, cell cycle progression, apoptosis, the inflammatory response, signal transduction, cell–cell recognition, cell adhesion, immune response, DNA repair, cell growth, differentiation and tumorigenesis (Table S4-4).

When further analysing the five unknown interactions, for three of the five gene pairs, a putative relationship could be inferred from Biograph (Table S4-5): CFLAR-LTB, PLCG2-ICAM1 and TNFRSF11A-CD14. These gene pairs could be connected through intermediates involved in inflammatory response, cell–cell communication and immune response, respectively. The interaction involved in cell–cell communication could also be predicted from STRING (Table S4-6). One of the five gene pairs (LBP-TNFSF14) could only be connected in CTD by its interaction with acetaminophen (Tables S4-7 and S4-8). For the remaining unknown gene interaction (LTB → ICAM1), no evidence for a functional relationship could be found in the consulted databases.

When DTNI analysis was repeated for 4 compounds not affecting the NF-kB pathway (in our case diazepam, indomethacin, omeprazole and hepatocyte growth factor), none of the 21 edges in Figure S4-1 could be inferred, which shows that the network in Figure S4-1 is specific for compounds affecting the NF-kB pathway.

#### Example 2: NRF2 pathway

By performing pathway analysis on the DEGs, we found ten compounds related to the NRF2 pathway: azathioprine, carbamazepine, coumarin, diazepam, flutamide, ketoconazole, lomustine, nitrofurantoin, propylthiouracil and valproic acid. DTNI, applied to this group of compounds, inferred an interaction network of 123 nodes, connected by 811 edges (Figure S5-1). Within the network, 196 connections (24.0%) are true-positive interactions, 23 are false-positive interactions (3.0%), and 592 connections are unknown (73.0%). Table S5-1 presents the details on the edges. The network has no subnetworks. Pathways related to the inferred network are listed in Table S5-2. The inferred network is related to solute carrier (SLC)-mediated transmembrane transport, biotransformation pathways (phase I/phase II), response to oxidative stress (e.g. degradation of reactive oxygen species), metabolism (carbohydrates, amino acids, nucleotides), aryl hydrocarbon receptor signalling, apoptosis, tumour protein p53 signalling, transforming growth factor beta signalling, gene regulation by peroxisome proliferators and ABC family proteins-mediated transport.

Within the complete network of 123 nodes, 13 were starting nodes. The heat map in Figure S5-2 shows that SLC5A9 is most affected at the earliest time point (2 h). SLC5A9 plays a role in transporter activity and metabolism, in particular the carbohydrate metabolism (Tazawa et al. 2005). The other genes in the inferred network are involved in metabolism, drug resistance, transporter activity, immune system, cell–cell communication, apoptosis, development, cell growth, tissue repair, tumorigenesis, cellular senescence, protection against oxidative stress, drug metabolism, protein folding and degradation, inflammatory response, redox regulation of the cell, survival, chemotaxis, circadian entrainment, detoxification and elimination of xenobiotics, cellular stress response, autophagy, cytoskeletal organization (Table S5-3).

For 63 of the 592 unknown interactions, a putative relationship could be inferred in BioGraph (Table S5-4). When restricting to the ten interactions in Biograph that have the highest interaction strength (determined by DTNI), we found that five gene pairs could only be related to each other because both genes react with NADP, water or oxygen (Table S5-5). Interaction between the other five gene pairs (SLC6A9-HSPA1A, MAFG-SLC39A7, SLC6A8-HSPA1A, TXN-HBEGF and PDGFB-NRG1) could be explained by a common biological function between the genes (Table S5-5). SLC6A9-HSPA1A could be connected by intermediates involved in inflammatory response. MAFG-SLC39A7, SLC6A8-HSPA1A and their intermediates play a role in cell signalling. The interaction of TXN and HBEGF through EGFR is part of the immune system. PDGFB-NRG1 and its intermediates play a role in cell–cell communication.

Thirteen interactions could be retrieved from STRING, of which 4 could also be inferred from Biograph (Table S5-8). One interaction was known from curated databases, two were experimentally determined, one gene pair was known to be co-expressed, and the remainder could be extracted with the help of text mining (Table S5-6). A total of 379 gene pairs could only be connected in CTD by their interaction with at least one of the 10 studied compounds (Tables S5-7 and S5-8). For the remaining 141 interactions, no evidence for a functional relationship could be found in the consulted databases.

When DTNI analysis was repeated for 10 compounds not affecting the NRF2 pathway (in our case indomethacin, omeprazole, hepatic growth factor, diclofenac, haloperidol, adapin, allopurinol, benzbromarone, ethionine and fluphenazine), 32 (3.9%) of the 811 edges in Figure S5-1 could be inferred, which shows that 96.1% of the network in Figure S5-1 is specific for compounds affecting the NRF2 pathway. Of the 32 edges in the overlap, 9 were true-positive interactions and 23 unknown in CPDB. The 9 true-positive interactions are relationships between genes from the NRF2 pathway that occur in other pathways, in particular xenobiotics metabolism by cytochrome P450 and transport pathways (Table S5-9). Of the 23 unknown interactions, none of the gene pairs has overlap in GO terms or an interaction in STRING. Only 4 of these 23 interactions could be inferred from known interactions in Biograph (Table S5-10). Three of the 32 interactions in the intersection have gene pairs that are affected by one of the 10 compounds (not affecting the NRF2 pathway) in CTD: TGFA-GCLC (both affected by diclofenac), TXN-HBEGF (both affected by diclofenac) and SLC7A11-ABCC3 (both affected by indomethacin).

## Discussion

Unravelling the mechanism of action (MoA) of compounds is a challenging topic in toxicology. Previous methods for elucidating MoA by analysing gene expression data on multiple time points and doses, such as DeMAND (Woo et al. 2015), require a pathway with its genes and interactions as input and test the dysregulation of the known interactions of each gene within this pathway, in order to provide a list of genes that are most dysregulated by the toxicant. In contrast to methods such as DeMAND, DTNI only requires a gene list of a pathway (or biological process) of interest as input and not its interactions. Therefore, DTNI not only tests (dys)regulation of existing interactions, but also infers novel interactions that can be tested experimentally. Furthermore, the output of DTNI is a list of interactions with their statistical significance, while methods such as DeMAND provide a list of genes with their statistical significance. As a consequence, DTNI provides information complementary to the output of DeMAND.

An interaction in a network inferred with DTNI can occur due to several reasons: (1) a direct gene–gene interaction; (2) an indirect gene–gene interaction (through one or more intermediates); (3) both genes affected by one or more of the studied compounds.

The ROC curves for the simulations show that DTNI outperforms TSNI in inferring a network induced by a toxicant. Moreover, DTNI allows including data from multiple perturbations (compounds), while only one perturbation can be included within TSNI. Including multiple compounds has the additional advantage that a LOOCV step can be applied, leaving out one compound, which further improves the proportion of direct interactions predicted by network inference that are true direct interactions (PPV).

In this study, we demonstrated the performance of DTNI on experimental data for two groups of compounds: those affecting the NF-kB pathway and those influencing the NRF2 pathway.

The network inferred for the NF-kB pathway is specific for compounds affecting this pathway and cannot be inferred when analysing a group of compounds not influencing the NF-kB pathway. For the NRF2 pathway, most of the network (96.1%) was specific for compounds affecting that pathway. This shows that DTNI can infer a network specific for compounds affecting a common pathway for a moderate number (≤90) genes, but also that the performance in terms of inferring a network specific for a group of compounds slightly decreases when evaluating larger networks.

In the first example, the NF-kB pathway, we found a network related to apoptosis and inflammation, which are key events in both the AOPs for drug-induced liver fibrosis and cholestasis (Vinken 2015). From the starting nodes already induced after 2 h of drug treatment, TLR4 was the most affected (inhibition, Figure S4-2). TLR4 has an important role in pathogen recognition and activates the innate immune system (Broering et al. 2011). Also, down-regulation of TLR4 has been related to liver cirrhosis (Manigold et al. 2003). This suggests that down-regulation of TLR4 is a potential MIE in these AOPs. From the four compounds affecting the NF-kB pathway, two compounds (acetaminophen and carbon tetrachloride) have been reported to affect the expression of TLR4 (Beyer et al. 2007; De Minicis et al. 2007). Furthermore, it has been shown that TLR4 activity increases susceptibility to acetaminophen (Yohe et al. 2006).

The network inferred for the NRF2 pathway is involved in pathways which are associated with key events indicated in AOPs for fibrosis (TGFB signalling, apoptosis) or cholestasis (oxidative stress, apoptosis) (Vinken 2015), or are related to liver cancer (p53 signalling (Hanahan and Weinberg 2011), SLC-mediated transmembrane transport (El-Gebali et al. 2013)). SLC5A9 (inhibition, Figure S5-2) was the most affected starting node at 2 h of treatment, which suggests that down-regulation of SLC5A9 is a potential MIE in an AOP for fibrosis, cholestasis or liver cancer. SLCA9 regulates sodium-dependent glucose transport (Tazawa et al. 2005). To our knowledge, a relationship between SLC5A9 and fibrosis, cholestasis or liver cancer has not yet been established, so this presents a novel finding which has to be verified experimentally. However, a relationship between SLCs in general and cancer has been reported (El-Gebali et al. 2013). The ten compounds influencing the NFR2 pathway were also not known to induce SLC5A9.

For the NF-kB pathway, the largest percentage of edges in the network (66.7%) is true-positive interactions, while only 9.5% of the edges were false-positive interactions. This shows that DTNI is a reliable method for analysing a group of genes of moderate size (≤90 genes). Furthermore, 23.8% of unknown interactions could be discovered. Three of the five unknown interactions in CPDB could be predicted from known relationships in Biograph and/or STRING. One interaction could only be explained by the influence of one of the studied compounds on both genes. For the remaining interaction, no evidence could be found in the consulted databases.

For the larger group of genes of the NRF2 pathway, the percentage of true-positive interactions was still high (24%) compared to the number of false-positive interactions (3%). Interestingly, the largest part of the network (73%) represents unknown interactions. It has to be noted that on the Wikipathways website, it is reported that the NRF2 pathway is not fully connected and probably incomplete (http://www.wikipathways.org/index.php/Pathway:WP2884). This could explain the large percentage of unknown interactions reported here. After further consulting of other biological databases (Biograph, STRING, CTD), 72 of these interactions could be explained from known interactions in Biograph and/or STRING. For 379 interactions, the only evidence for an interaction was that at least one of the studied compounds affected both genes. For the remaining 141 interactions, no evidence could be extracted.

In summary, these two examples show that DTNI analysis is a suitable method for causally inferring key events and potential MIEs in AOPs across dose and time when the number of genes analysed is of moderate size. DTNI also detects novel interactions which have to be verified experimentally, e.g. by gene knockdown experiments.

## Electronic supplementary material

Below is the link to the electronic supplementary material.

Supplementary material 1 (PDF 434 kb)

Supplementary material 2 (PDF 569 kb)

Supplementary material 3 (PDF 378 kb)

Supplementary material 4 (PDF 948 kb)

Supplementary material 5 (PDF 734 kb)

Supplementary material 6 (PDF 2325 kb)
