# Supplementary material for: DTNI: a novel toxicogenomics data analysis tool for identifying the molecular mechanisms underlying the adverse effects of toxic compounds
Source: Arch Toxicol. 2016 Dec 28;91(6):2343–52. doi: 10.1007/s00204-016-1922-5 (PMC5429357; doi:10.1007/s00204-016-1922-5)
Supplement: Supplementary file 1 — Supplementary material 1 (PDF 434 kb) [file 204_2016_1922_MOESM1_ESM.pdf]

Guide to the Supplementary Material

Article title: DTNI: a novel toxicogenomics data analysis tool for identifying the molecular mechanisms underlying the adverse effects of toxic compounds

Journal name: Archives of Toxicology

Authors names: Diana M. Hendrickx[1], Terezinha Souza[1], Danyel G. J. Jennen[1], Jos C. S. Kleinjans[1]

Affiliation: [1] Department of Toxicogenomics, GROW-School for Oncology and Developmental Biology, Maastricht University, Universiteitssingel 40, 6229 ER Maastricht, The Netherlands. Postal address: P.O. Box 616, 6200 MD Maastricht, The Netherlands. Telephone: +31 43 3881845.

E-mail address of the corresponding author: Diana M. Hendrickx, d.hendrickx@maastrichtuniversity.nl

Supplementary Material 1

Description of DTNI

ODE model p.2

Interpolation step p.2

Discretization of ODE p.3

Dimension reduction step p.3

Adapted permutation test p.4

Calculation of interaction matrix p.4

Multiple compounds p.4

Supplementary Material 2

Matlab code

Main script: main.m – description p.2

Write Excel-file for further analysis: write_cytoscape_input.m – description p.2

Scripts – code that can be copied into the Matlab editor p.2-11

Supplementary Material 3

Model validation – additional information

Simulations p.2-6

Validation on real experiments – PPAR signalling pathway p.7-9

Figures:

Figure S3-1: **p.2**
Interaction network for the simulation model

Figure S3-2: **p.5**
Comparison of DTNI and TSNI for simulated data of a single compound with three time points (2h, 8h, 24h), three doses (low, middle, high) and time-matched controls (dose = 0). ROC-curve for TSNI (circles) and DTNI (triangles).

Figure S3-3: **p.5**
a) Influence of the number of time points on the performance of DTNI for simulated data of a single compound with three doses (low, middle, high) and time-matched controls (dose = 0). Plot of geometric mean score versus number of time points for a p-value threshold of 0.05. b) Influence of the number of doses on the performance of DTNI for simulated data of a single compound with three time points (2h, 8h, 24h) and time-matched controls (dose = 0). Plot of geometric mean score versus number of doses for a p-value threshold of 0.05. c) Influence of the number of compounds on the performance of DTNI for simulated data with three time points (2h, 8h, 24h), three doses (low, middle, high) and time-matched controls (dose = 0). Plot of geometric mean score versus number of compounds for a p-value threshold of 0.05. d) Influence of noise on the performance of DTNI for simulated data of a single compound with three time points (2h, 8h, 24h), three doses (low, middle, high) and time-matched controls (dose = 0). ▽: ROC curve for noiseless simulated data; □: average ROC curve for 10 simulated data sets with 15% noise and no replicates; ◊: average ROC curve for 5 simulated data sets with 15% noise and two replicates.

Figure S3-4: **p.6**
Influence of LOOCV on the performance of DTNI for simulated data of 6 compounds with three time points (2h, 8h, 24h), three doses (low, middle, high) and time-matched controls (dose = 0). Plot of positive predictive value versus number of LOOCV data sets for a p-value threshold of 0.05.

Tables:

Table S3_1: p.2
Drug reactions added to the simulation model

Table S3_2: p.3
Overview of the simulations performed in this study.
Table S3_3: p.3
Initial values for the variables in the model, used for all simulations.

Table S3_4: p.4
Values of the doses (μmol/l) of the drugs for each simulation

Table S3_5: p.4
Influence of the number of time points – values of the time points in the simulated data sets.

Table S3-6: p.7
Results of DTNI analysis of the genes of the PPAR signalling pathway

Table S3-7: p.8
Comparison of genes in the inferred network for clofibrate with prior information in CTD and Fruchart et al.

Supplementary Material 4

Example 1: NF-kB pathway – additional information

Figures:

Figure S4-1: p.2
NF-kB pathway - interaction network inferred with DTNI for p ≤ 0.05. Green edges: true positives (TP), red edges: false positives (FP), black edges: novel interactions. Subnetworks within the inferred network are indicated with ellipses.

Figure S4-2: p.7
Heat map for the 12 start nodes (nodes with only outgoing edges) in the network of Figure 1, displaying the log2 ratios at the earliest time point (2 hours). AC = acetaminophen; CT = carbon tetrachloride; IL1 = interleukin 1; IL6 = interleukin 6; L = low dose; M = middle dose; H = high dose.

Tables:

Table S4-1: p.3
Details of the edges in the network of Figure S4-1. PI = protein interaction; BI = biochemical interaction.

Table S4-2: p.4-5
Pathways (q-value ≤ 0.05) in ConsensusPathDB related to the network in Figure S4-1. Disease pathways of non-liver diseases were removed from the table.

Table S4-3: p.6-7
Pathways (q-value ≤ 0.05) in ConsensusPathDB related to the subnetworks in Figure S4-1. Disease pathways of non-liver diseases were removed from the table.

Table S4-4: p.8
Function of the genes in the network of Figure 1, extracted from GeneCards.

Table S4-5: p.8
Unknown interactions in CPDB – putative functional relationships inferred with Biograph, based on known interactions. Gene pairs without functional relationships in BioGraph were omitted from the table. E = expression, PI = protein interaction, GI = genetic interaction, P = phosphorylation.

Table S4-6: p.8
Unknown interactions in CPDB – gene pairs with functional interaction in STRING. Gene pairs without functional relationship in STRING were omitted from the table.

Table S4-7: p.9
Unknown interactions in CPDB – relation of gene pairs with one (or more) of the four studied compounds, extracted from CTD. Gene pairs that have no relationship with one of the compounds in CTD were omitted from the table.

Table S4-8: p.9
Unknown interactions in CPDB – evidence for a relationship from other databases – summary of Tables S4-6, S4-7, S4-8. Interactions for which no evidence could be found were omitted.

Supplementary Material 5

Example 2: NRF2 pathway – additional information

Figures:

Figure S5-1: **p.2**
NRF2 pathway – interaction network inferred with DTNI for $p \leq 0.05$. Green edges: true positives (TP), red edges: false positives (FP), black edges: novel interactions.

Figure S5-2: Heatmap for the 13 start nodes (nodes with only outgoing edges) in the network of Figure S5-1, displaying the log2 ratios at the earliest time point (2 hours). AZ = azathioprine; CA = carbamazepine; CO = coumarin; DI = diazepam; FL = flutamide; KE = ketoconazole; LO = lomustine; NI = nitrofurantoin; PR = propylthiouracil; VA = valproic acid; L = low dose; M = middle dose; H = high dose.

Tables:

Table S5-1: **p.3-18**
Details of the edges in the network of Figure S5-1. PI = protein interaction; BI = biochemical interaction.

Table S5-2: **p.19-20**
Pathways (q-value ≤ 0.05) in ConsensusPathDB related to the network in Figure S5-1. Disease pathways of non-liver diseases were removed from the table.

Table S5-3: **p.22-23**
Function of the genes in the network of Figure S5-1, extracted from GeneCards.

Table S5-4: **p.24-25**
Unknown interactions in CPDB – putative functional relationships inferred with Biograph, based on known interactions. Gene pairs without functional relationships in BioGraph were omitted from the table.

Table S5-5: **p.26**
Top ten interactions in Biograph, based on absolute value of the interaction strength determined by DTNI – common function of the genes.

Table S5-6: **p.26**
Unknown interactions in CPDB – gene pairs with functional interaction in STRING. Gene pairs without functional relationship in STRING were omitted from the table.

Table S5-7: **p.27-33**
Unknown interactions in CPDB – relation of gene pairs with one (or more) of the ten studied compounds, extracted from CTD. Gene pairs that have no relationship with one of the compounds in CTD were omitted from the table.

Table S5-8: **p.34-41**
Unknown interactions in CPDB – evidence for a relationship from other databases – summary of Tables S5-5, S5-6, S5-7, S5-8. Interactions for which no evidence could be found were omitted. MF = molecular function, BP = biological process, CC = cellular component.

Table S5-9: **p.41**
True positive edges in Figure S5-1 that could also be inferred when analysing 10 compounds not affecting the NRF2 pathway.

Table S5-10: **p.41**
Unknown edges in Figure S5-1 that could also be inferred when analysing 10 compounds not affecting the NRF2 pathway.
