# Supplementary material for: DTNI: a novel toxicogenomics data analysis tool for identifying the molecular mechanisms underlying the adverse effects of toxic compounds
Source: Arch Toxicol. 2016 Dec 28;91(6):2343–52. doi: 10.1007/s00204-016-1922-5 (PMC5429357; doi:10.1007/s00204-016-1922-5)
Supplement: Supplementary file 2 — Supplementary material 2 (PDF 569 kb) [file 204_2016_1922_MOESM2_ESM.pdf]

Supplementary Material 1

Article title: DTNI: a novel toxicogenomics data analysis tool for identifying the molecular mechanisms underlying the adverse effects of toxic compounds

Journal name: Archives of Toxicology

Authors names: Diana M. Hendrickx[1], Terezinha Souza[1], Danyel G. J. Jennen[1], Jos C. S. Kleinjans[1]

Affiliation: [1] Department of Toxicogenomics, Maastricht University, GROW-School for Oncology and Developmental Biology, Universiteitssingel 40, 6229 ER Maastricht, The Netherlands. Postal address: P.O. Box 616, 6200 MD Maastricht, The Netherlands. Telephone: +31 43 3881845.

E-mail address of the corresponding author: Diana M. Hendrickx, d.hendrickx@maastrichtuniversity.nl

Description of DTNI

ODE model

Dose-Time Network Identification (DTNI) is an extension of Time Series Network Identification (TSNI) (Bansal et al. 2006). For a data set of n genes, measured at m time points for p doses, the model uses the following system of (linear) ordinary differential equations (ODE):

$$\frac{\partial x_i}{\partial t}(t_k, d_l) = \sum_{j=1}^{n} a_{(time)_{ij}} x_j(t_k, d_m) + b_i d_l \quad (1)$$

$$\frac{\partial x_i}{\partial d}(t_k, d_l) = \sum_{j=1}^{n} a_{(dose)_{ij}} x_j(t_k, d_m) + c_i t_k \quad (2)$$

where $i = 1, \dots, n$; $k = 1, \dots, m$; $l = 1, \dots, p$; $x_i(t_k, d_m)$ is the expression of gene i measured at time t_k for dose d_l; $\frac{\partial x_i}{\partial t}(t_k, d_l)$ is the rate of change of the expression of gene i over time at time t_k for dose d_l; $\frac{\partial x_i}{\partial d}(t_k, d_l)$ is the rate of change of the expression of gene i over dose at time t_k for dose d_l; $a_{(time)_{ij}}$ is the influence of gene j on gene i due to a change in time; $a_{(dose)_{ij}}$ is the influence of gene j on gene i due to a change in dose; b_i is the effect of the perturbation (exposure) on gene i; c_i is the effect of the duration of exposure on gene i; t_k is the kth time point; d_l is the lth dose. The coefficients $a_{(time)_{ij}}$ and $a_{(dose)_{ij}}$ are also called interaction strength and are zero for no interaction, positive for activation and negative for repression.
In matrix notation, the system of ODE can be written as

$$\frac{\partial X}{\partial t}(t_k, d_l) = A_{(time)} X(t_k, d_l) + B d_l \quad (3)$$

$$\frac{\partial X}{\partial d}(t_k, d_l) = A_{(dose)} X(t_k, d_l) + C t_k \quad (4)$$

where $X(t_k, d_l)$ is a $(n \times 1)$ vector of expression of n genes at time t_k for dose d_l; $\frac{\partial X}{\partial t}(t_k, d_l)$ is a $(n \times 1)$ vector of first derivatives of X with respect to time at time t_k for dose d_l; $\frac{\partial X}{\partial d}(t_k, d_l)$ is a $(n \times 1)$ vector of first derivatives of X with respect to dose at time t_k for dose d_l; $A_{(time)}$ is a $(n \times n)$ matrix of influences of the genes on each other due to a change in time; $A_{(dose)}$ is a $(n \times n)$ matrix of influences of the genes on each other due to a change in time. B is a $(n \times 1)$ vector representing the effect of the perturbation (exposure) on each gene; C is a $(n \times 1)$ vector representing the effect of the duration of the exposure on each gene.

Interpolation step

Because in toxicogenomics data the number of time points and doses is low and the data are not equally sampled, an interpolation step is performed for both time and dose series.

Interpolation step for time series

For a data set with m time points within the time interval [0,T], we calculated a new time series of 2m equally sampled time points $\frac{T}{2m}, \frac{2T}{2m}, \dots, \frac{2mT}{2m}$ = T, using cubic smoothing spline interpolation (De Boor 2001) with smoothing parameters taken from TSNI (Bansal et al. 2006).

Interpolation step for dose series

For a data set with p doses within the dose interval [0,D], we calculated a new dose series of 2p equally sampled dose points $\frac{D}{2p}, \frac{2D}{2p}, \dots, \frac{2pD}{2p}$ = D, using cubic Hermite interpolation (Kahaner et al. 1988).
Using different interpolation methods for time and dose series is necessary, because cubic smoothing spline interpolation performs bad for dose series, while performance is good for time series (see Figures S1-1a and S1-1b). Hermite interpolation performs good for dose series, while it performs bad for time series (see Figures S1-1c and S1-1d).

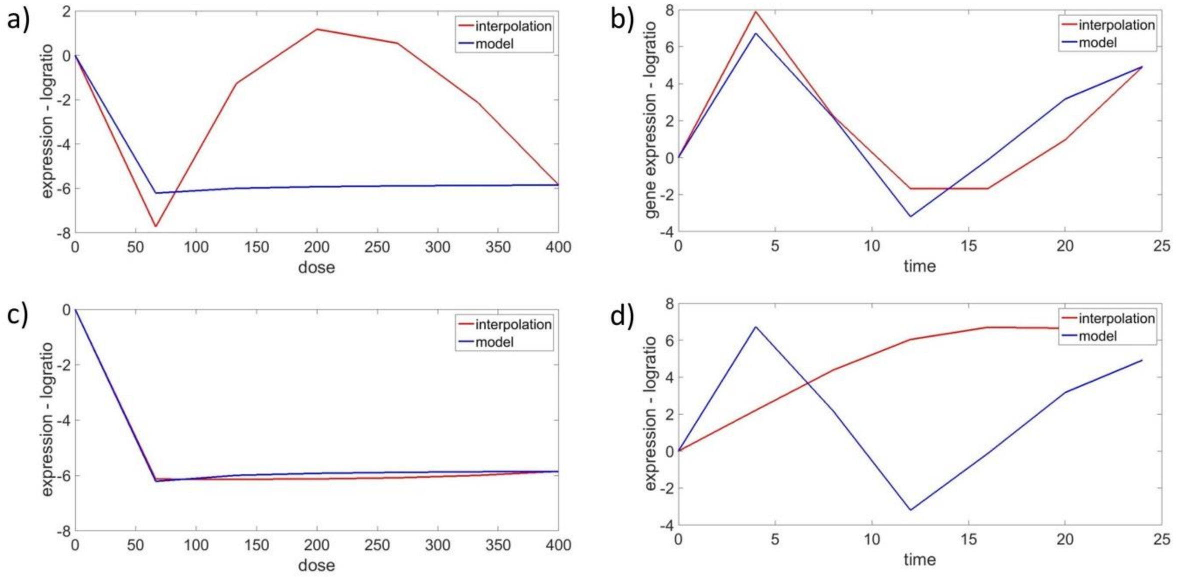

Figure S1-1: a) blue: curve connecting simulated gene expression data for 6 non-zero doses + dose 0 with the simulation model of the IkB-NF-kB signalling module; red: cubic spline interpolation through dose 0, 16, 80, 400. b) blue: curve connecting simulated gene expression data for 6 non-zero time points + time 0 with the simulation model of the IkB-NF-kB signalling module; red: cubic spline interpolation through time 0, 2, 8, 24. c) blue: curve connecting simulated gene expression data for 6 non-zero doses + dose 0 with the simulation model of the IkB-NF-kB signalling module; red: hermite interpolation through dose 0, 16, 80, 400. d) blue: curve connecting simulated gene expression data for 6 non-zero time points + time 0 with the simulation model of the IkB-NF-kB signalling module; red: hermite interpolation through time 0, 2, 8, 24.

Discretization of ODE

In ODE-based network inference methods, derivatives are mostly calculated from the equation of the fitted curve during the interpolation step (Willemsen et al. 2015). However, in case of data with high noise levels, like transcriptomics data, this will introduce more noise and decrease the accuracy of the method. This problem can be circumvented by converting the ODE to their discrete form (Bansal et al. 2006). The discrete form of an ODE is an equation that describes the biological variables at each point in function of the previous point. After conversion of the system of ODE in discrete form like described in (Ljung 1999), equations (3) and (4) become

$$X(t_{k+1}, d_l) = A_{(discr,time)}X(t_k, d_l) + B_{discr}d_l \quad (5)$$

$$X(t_k, d_{l+1}) = A_{(discr,dose)}X(t_k, d_l) + C_{discr}t_k \quad (6)$$

where $A_{(discr,time)}$, $A_{(discr,dose)}$, B_{discr} and C_{discr} are the discrete counterparts of $A_{(time)}$, $A_{(dose)}$, B and C respectively.

Equations (5) and (6) can also be written as

$$X(t_{k+1}, d_l) = [A_{(discr,time)} \quad B_{discr}] \begin{bmatrix} X(t_k, d_l) \\ d_l \end{bmatrix} \quad (7)$$

$$X(t_k, d_{l+1}) = [A_{(discr,dose)} \quad C_{discr}] \begin{bmatrix} X(t_k, d_l) \\ t_k \end{bmatrix} \quad (8)$$

Dimension reduction step

Because in toxicogenomics (and transcriptomics in general) the number of variables (genes) is much larger than the number of samples (time points, doses), the system of ODE often does not have a unique solution. To avoid these identifiability problems, we combine our ODE based method with a dimension reduction step.

Put $X_1(d_l) = [X(t_0, d_l) \quad \dots \quad X(t_{m-1}, d_l)]$ $(l = 1, \dots, p)$; $X_2(d_l) = [X(t_1, d_l) \quad \dots \quad X(t_m, d_l)]$ $(l = 1, \dots, p)$; $X_1(t_k) = [X(t_k, d_0) \quad \dots \quad X(t_k, d_{p-1})]$ $(k = 1, \dots, m)$; $X_2(t_k) = [X(t_k, d_1) \quad \dots \quad X(t_k, d_p]$ $(k = 1, \dots, m)$; $D_l = [d_l \quad \dots \quad d_l]$ $(1 \times m)$ row vector; $T_k = [t_k \quad \dots \quad t_k]$ $(1 \times p)$ row vector; $H_d = [A_{(discr,time)} \quad B_{discr}]$; $K_d = [A_{(discr,dose)} \quad C_{discr}]$; $L_l = \begin{bmatrix} X_1(d_l) \\ D_l \end{bmatrix}$ $(l = 1, \dots, p)$; $M_k = \begin{bmatrix} X_1(t_k) \\ T_k \end{bmatrix}$ $(k = 1, \dots, m)$.

Then the system of ODE can also be written as

$$X = HY \quad (9)$$

where $X = \begin{bmatrix} X_2(d_1) & \dots & X_2(d_p) & 0 & \dots & 0 \\ 0 & \dots & 0 & X_2(t_1) & \dots & X_2(t_m) \end{bmatrix}$, $H = \begin{bmatrix} H_d & 0 \\ 0 & K_d \end{bmatrix}$, $Y = \begin{bmatrix} L_1 & \dots & L_q & 0 & \dots & 0 \\ 0 & \dots & 0 & M_1 & \dots & M_p \end{bmatrix}$.
To perform the dimension reduction step, the singular value decomposition of Y is calculated: $Y = VDT'$, where V is the matrix of left singular vectors, D is a diagonal matrix with the singular values on the diagonal and T is the matrix of right singular vectors. Then we choose s top singular values. The system of ODE in the reduced dimensional space can be written as

$$X = Z_d Y_R \qquad (10)$$

where Z_d = the first s columns of HV and Y_R = the first s rows of DT'.
The system of ODE in the reduced dimension space is solved by least squares estimation, taking the pseudo-inverse of Y_R:

$$Z_d = XY_R'(Y_R Y_R')^{-1} \qquad (11)$$

Then the solution Z_d is projected to the original dimensional space. Therefore we put $Z = [Z_d \quad 0]$, where Z is obtained by adding columns of zeros to Z_d until it has the same dimension as HV. Then $H = ZV'$ is the solution of the ODE in the original space. If we know H, then we know the solution of the ODE in discrete space, i.e. $A_{(discr,time)}$, $A_{(discr,dose)}$, B_{discr}, C_{discr}. The solution of the original system of ODE (in continuous form, equations (3) and (4)) can then be obtained by applying a bilinear transformation (Ljung 1999):

$$A_{(time)} = \frac{2}{\Delta t} \frac{A_{(discr,time)} - I}{A_{(discr,time)} + I} \qquad (12)$$

$$A_{(dose)} = \frac{2}{\Delta d} \frac{A_{(discr,dose)} - I}{A_{(discr,dose)} + I} \qquad (13)$$

$$B = (A_{(discr,time)} + I)^{-1} A_t B_{discr} \qquad (14)$$

$$C = (A_{(discr,dose)} + I)^{-1} A_d C_{discr} \qquad (15)$$

where Δt is the time interval, Δd is the dose interval and I the $(n \times n)$ identity matrix.
The procedure above is repeated for all possible values of s, and we selected the optimal value of s for which the sum of squares of the residuals is minimized. The ODE solution for the optimal value of s will be kept for further calculations.

Adapted permutation test

Significance testing was done by permuting the residuals 1000 time. This results in matrices $P_{(A,time)}$ and $P_{(A,dose)}$ of p-values for the interaction strengths in matrices $A_{(time)}$ and $A_{(dose)}$ respectively.

Calculation of interaction matrix

From the p-values and the interaction strengths, interaction matrices $A_{(int,time)}$ and $A_{(int,dose)}$ were calculated for p-value thresholds of 0.01 and 0.05, where for $i, j = 1, \dots, n$:

$$A_{(int,time)}(i,j) = A_{(time)}(i,j) \text{ when } P_{(A,time)}(i,j) \leq p \text{ value threshold}$$
$$A_{(int,time)}(i,j) = 0 \qquad \text{when } P_{(A,time)}(i,j) > p \text{ value threshold}$$

$$A_{(int,dose)}(i,j) = A_{(dose)}(i,j) \text{ when } P_{(A,dose)}(i,j) \leq p \text{ value threshold}$$
$$A_{(int,dose)}(i,j) = 0 \qquad \text{when } P_{(A,dose)}(i,j) > p \text{ value threshold}$$

Then a general interaction matrix A_{int} is calculated by averaging $A_{(int,time)}$ and $A_{(int,dose)}$.

Multiple compounds

In case we analyse data from multiple compounds, an interaction matrix for dose is calculated for each compound.

References

Bansal M, Della Gatta G, di Bernardo D (2006) Inference of gene regulatory networks and compound mode of action from time course gene expression profiles. Bioinformatics 22(7):815-22
De Boor C (2001) A practical guide to splines, Revised Edition. Springer-Verlag New York
Kahaner D, Moler C, Nash S (1988) Numerical Methods and Software. Prentice Hall, Upper Saddle River, NJ
Ljung J (1999) System Identification: Theory for the User. Prentice Hall, Upper Saddle River, NJ

Willemsen AM, Hendrickx DM, Hoefsloot HC, et al. (2015) MetDFBA: incorporating time-resolved metabolomics measurements into dynamic flux balance analysis. Mol Biosyst 11(1):137-45
