# Supplementary material for: DTNI: a novel toxicogenomics data analysis tool for identifying the molecular mechanisms underlying the adverse effects of toxic compounds
Source: Arch Toxicol. 2016 Dec 28;91(6):2343–52. doi: 10.1007/s00204-016-1922-5 (PMC5429357; doi:10.1007/s00204-016-1922-5)
Supplement: Supplementary file 3 — Supplementary material 3 (PDF 378 kb) [file 204_2016_1922_MOESM3_ESM.pdf]

Supplementary Material 2

Article title: DTNI: a novel toxicogenomics data analysis tool for identifying the molecular mechanisms underlying the adverse effects of toxic compounds

Journal name: Archives of Toxicology

Authors names: Diana M. Hendrickx[1], Terezinha Souza[1], Danyel G. J. Jennen[1], Jos C. S. Kleinjans[1]

Affiliation: [1] Department of Toxicogenomics, GROW-School for Oncology and Developmental Biology, Maastricht University, Universiteitssingel 40, 6229 ER Maastricht, The Netherlands. Postal address: P.O. Box 616, 6200 MD Maastricht, The Netherlands. Telephone: +31 43 3881845.

E-mail address of the corresponding author: Diana M. Hendrickx, d.hendrickx@maastrichtuniversity.nl

Matlab code

The scripts below make use of the following online available scripts:

ssq.m — calculates the sum of squares of a matrix
available from : http://www.bdagroup.nl/content/Downloads/software/software.php
Click on the link "Fourth order approximation of the time derivatives of metabolite concentrations and calculation of the Jacobian" to download the folder "jacobian.zip".
The ssq.m script is in this folder.

interpolation_data.m — to perform the interpolation step for time series
available from:
http://dibernardo.tigem.it/softwares/time-series-network-identification-tsni
Click on the link to the Matlab code to download the folder "tsni.zip".
The interpolation_data.m script is in this folder.

Main script: main.m

To calculate the matrix of interaction strengths and the type of interaction (activation/repression/none) with DTNI, the user has to run the script "main.m". This script uses the following Matlab scripts:

calc_interactions.m
discretization_DTNI.m
DTNI.m
interpolation_data.m
perm_res.m
resid_model.m
ssq.m
ssqmatrix.m

Write Excel-file for further analysis: write_cytoscape_input.m

The script "write_cytoscape_input.m" writes an Excel file that e.g. can be used to import the network into Cytoscape (Shannon et al. 2003) (http://www.cytoscape.org/) for visualization.

Scripts

---

main.m

```
function [intA,interaction_type] =
main(data,time_points,doses,no_compounds,no_genes,no_samples,no_doses,threshold)
%calculates the matrix of interaction strengths (intA) and the type of
%interaction (interaction_type). 0=no interaction; 1=activation; -1=repression

%input
%------
%data: gene expression data, average log2 ratios
%   rows=genes; column=samples
%   Columns have to be ordered as follows (for p compounds, q time points, r doses):
%   column 1: compound 1 at time 1 and dose 1
%   ...
%   column r: compound 1 at time 1 and dose r
%   column r+1: compound 1 at time 2 and dose 1
%   ...
%   column 2*r: compound 1 at time 2 and dose r
%   ...
%   column (q-1)*r+1: compound 1 at time q and dose 1
%   ...
%   column q*r: compound 1 at time q and dose r
%   column q*r + 1: compound 2 at time 1 and dose 1
%   ...
```

```
%   column 2*q*r: compound 2 at time q and dose r
%   ...
%   column (p-1)*q*r+1: compound p at time 1 and dose 1
%   ...
%   column p*q*r: compound p at time q and dose r
%time points: row vector of time points
%doses: row vector of doses, ordered as follows:
%   compound 1 dose 1, ..., compound 1 dose r, ... , compound p dose 1,
%   compound p dose r
%no_compounds: number of compounds
%no_genes: number of genes
%no_samples: number of time points * number of doses per compound
%no_doses: number of doses per compound
%threshold: p-value threshold

%find the best value for parameter l = number of latent variables (PC)
SSQR = ssqmatrix(data,time_points,doses,no_compounds,no_genes,no_samples,no_doses);
l=find(SSQR==min(SSQR));
l=min(l);
%calculate parameters for best model
[At,B,Ads,Cs,total_states,Kds] = DTNI(data,time_points,doses,no_compounds,l);
for j=1:no_compounds
    [residuals(((j-1)*no_genes+1):(j*no_genes),:),model(((j-1)*no_genes+1):(j*no_genes),:)] =
resid_model(data(:,((j-1)*no_samples+1):(j*no_samples)),time_points,doses(:,((j-
1)*no_doses+1):(j*no_doses)),Kds(((j-1)*no_genes+1):(j*no_genes),:));
end
%permutation test
[pvalAt,pvalAds]=perm_res(data,At,Ads,no_compounds,residuals,model,time_points,doses,total_states
);
%calculates interaction strengths intA and interaction type
%(activation/repression/inhibition)
[intA,interaction_type]=calc_interactions(At,Ads,pvalAt,pvalAds,no_compounds,no_genes,threshold);
save('workspace_DTNI')
```

---

calc_interactions.m

```
function
[intA,interaction_type]=calc_interactions(At,Ads,pvalAt,pvalAds,no_compounds,no_genes,threshold)
% This function is called by main.m and calculates a matrix of interactions
% strengths (intA) and a matrix of interaction types (interaction_type) from the output of
% DTNI.m and perm_res.m
% input:
% At,Ads: interaction matrices (output of DTNI.m)
% pvalAt,pvalAds: p-values of the interactions (output of perm_res.m)
% no_compounds: number of compounds in the data set
% no_genes: number of genes in the data set
% threshold: p-value threshold

r=size(pvalAds,1);
s=size(pvalAds,2);
for i=1:r
    for j=1:s
        if pvalAds(i,j)<=threshold
            intAds(i,j)=Ads(i,j);
        else
            intAds(i,j)=0;
        end
    end
end
for i=1:s
    for j=1:s
        if pvalAt(i,j)<=threshold
            intAt(i,j)=At(i,j);
        else
            intAt(i,j)=0;
        end
    end
```

```
end
intA=intAt;
for i=1:no_compounds
    intAdi=intAds(((i-1)*no_genes + 1):(i*no_genes),:);
    intA=intA+intAdi;
end
intA=(1/(no_compounds+1))*intA;
interaction_type=[];
for i=1:no_genes
    for j=1:no_genes
        if intA(i,j)<0
            interaction_type(i,j)=-1;
        elseif intA(i,j)>0
            interaction_type(i,j)=1;
        else
            interaction_type(i,j)=0;
        end
    end
end
```

---

discretization_DTNI.m

```
function [At,B,Ads,Cs,total_states,Kds] =
discretization_DTNI(D,T,no_compounds,doses,time_points,to_interpolate_time,to_interpolate_dose,pr
incipal_components)

% This function is called by DTNI.m and estimates the parameters
% At,B,Ads,Cs of the ODE.

% This code is adapted from the discretization.m code, published in
% the supplementary data of Bansal, M., Della Gatta G, di Bernardo D.(2006).
% Inference of gene regulatory networks and compound mode of action from
% time course gene expression profiles. Bioinformatics,22(7):815-822.

% input:
% D: output of fitting time series (done within the DTNI.m script)
% T: output of fitting dose series (done within the DTNI.m script)
% no_compounds: number of compounds in the data
% doses: a vector of doses for the original data set
% time_points: a vector of time points for the original data set
% to_interpolate_time: time points for interpolation step (determined within the DTNI.m script)
% to_interpolate_dose: doses for interpolation step (determined within the DTNI.m script)
% principal components: selected number of PCs - put zero if you have no idea how many PCs to
keep

% output:
% At: interaction matrix (time Jacobian). It is a fully connected network.
% Ads: interaction matrices (dose Jacobians). Fully connected networks.
% B: dose dependence parameters for each gene.
% C: time dependence parameters for each gene.
% total_states: Number of principal components used.
% Kds: matrix containing dose interaction matrices and time dependence parameters in discrete
dose space

%---------------------------------------------------
% construction of matrix Y1 (matrix left hand side)
%---------------------------------------------------
% matrix Y
no_rows_Y=2*size(D,1);
no_columns_Y=(size(D,3)-1)*size(D,2)+(size(T,2)-1)*size(T,3);
Y=zeros(no_rows_Y,no_columns_Y);
d3=size(D,3)-1;
t2=size(T,2)-1;
no_genes=size(D,1)/no_compounds;
r2=d3*size(D,2);
for j=1:no_compounds
```

```
    for i=1:size(D,2)
        Y((2*no_genes*(j-1)+1):((2*j-1)*no_genes),((i-1)*d3+1):(i*d3))=D((no_genes*(j-
1)+1):(j*no_genes),i,2:size(D,3));
    end
    for i=1:size(T,3)
        Y(((2*j-1)*no_genes+1):(2*j*no_genes),((i-1)*t2+1+r2):(i*t2+r2))=T((no_genes*(j-
1)+1):(j*no_genes),2:size(T,2),i);
    end
end

%Matrix Y1 (matrix left hand side)
dim1=(no_compounds+1)*no_genes;
a=size(D,2)*(size(D,3)-1);
b=size(T,3)*(size(T,2)-1);
dim2=(a+b)*no_compounds;
Y1=zeros(dim1,dim2);
for j=1:no_compounds
    Y1(1:no_genes,((j-1)*a+1):j*a)=Y(((j-1)*2*no_genes+1):((j-1)*2*no_genes+no_genes),1:a);
    Y1((j*no_genes+1):((j+1)*no_genes),(a*no_compounds+b*(j-1)+1):(a*no_compounds+b*j))=Y(((j-
1)*2*no_genes+no_genes+1):((j-1)*2*no_genes+2*no_genes),(a+1):(a+b));
end

%---------------------------------------------------
% construction of matrix ph (matrix right hand side)
%---------------------------------------------------
% construction of matrix ph1
no_rows_ph1=no_compounds*2*(no_genes+1);
no_columns_ph1=no_columns_Y;
ph1=zeros(no_rows_ph1,no_columns_ph1);
for j=1:no_compounds
    for i=1:size(D,2)
        ph1(((j-1)*(2*no_genes+2)+1):((j-1)*(2*no_genes+2)+no_genes),((i-1)*d3+1):(i*d3))=D(((j-
1)*no_genes+1):(j*no_genes),i,1:d3);
        ph1(((j-1)*(2*no_genes+2)+no_genes+1),((i-1)*d3+1):(i*d3))=doses((j-
1)*(size(doses,2)/no_compounds)+i)*ones(1,d3);
    end
    for i=1:size(T,3)
        ph1(((j-1)*(2*no_genes+2)+no_genes+2):((j-1)*(2*no_genes+2)+2*no_genes+1),((i-
1)*t2+1+r2):(i*t2+r2))=T(((j-1)*no_genes+1):(j*no_genes),1:t2,i);
        ph1(((j-1)*(2*no_genes+2)+2*no_genes+2),((i-
1)*t2+1+r2):(i*t2+r2))=time_points(i)*ones(1,t2);
    end
end

% construction of matrix ph
dimph1=(no_compounds+1)*no_genes + 2*no_compounds;
dimph2=(a+b)*no_compounds;
ph=zeros(dimph1,dimph2);
for j=1:no_compounds
    ph(1:no_genes,((j-1)*a+1):j*a)=ph1(((j-1)*(2*no_genes+2)+1):((j-
1)*(2*no_genes+2)+no_genes),1:a);
    ph(no_genes+j,((j-1)*a+1):j*a)=ph1(((j-1)*(2*no_genes+2)+no_genes+1),1:a);
    ph((j*no_genes+no_compounds+j):((j+1)*no_genes+no_compounds+j),(no_compounds*a+(j-
1)*b+1):(no_compounds*a+j*b))=ph1(((j-1)*(2*no_genes+2)+no_genes+2):((j-
1)*(2*no_genes+2)+2*no_genes+2),(a+1):(a+b));
end

[s1,s2,s3] = svd(ph);
diagonal = diag(s2);

% number of PCs based on singular value ratio
states = 0;
for j =  1:length(diagonal)
    condition = s2(1,1)/s2(j,j);
    if condition <= 100
        states = states +1;
    end
end

% number of PCs not specified by the user => use number of PCs calculated above
clear diagonal condition
```

```
Z = s1'*ph;
Z2=Z;
if principal_components == 0
    total_states = states;
else
    total_states = principal_components;
end

Z = Z(1:total_states,:);

% least squares solution for ODE parameters
    for i=1:size(Y1,1)
        result1 = Y1(i,:)*pinv(Z);
        result1(1,total_states+1:size(ph,1)) = 0;
        result(i,:) = result1*s1';
    end

%%% Results
Hd=result(1:no_genes,1:(no_genes+no_compounds));
for j=1:no_compounds
    Kds(((j-
1)*no_genes+1):(j*no_genes),:)=result((j*no_genes+1):((j+1)*no_genes),(j*no_genes+no_compounds+j)
:((j+1)*no_genes+no_compounds+j));
end

Adt=Hd(:,1:no_genes); % network At in discrete time space
Bdt=Hd(:,(no_genes+1):(no_genes+no_compounds)); % dose dependence parameters inferred in discrete
time space
Adds=Kds(:,1:no_genes); % networks Ad in discrete dose space (all compounds)
Cdds=Kds(:,no_genes+1); % time dependence parameters inferred in discrete dose space (all
compounds)
%%%%
At = (2/(to_interpolate_time(2) - to_interpolate_time(1)))*(Adt-
1*eye(no_genes))*pinv(Adt+1*eye(no_genes));  %%% Bilinear transformation
B = pinv(Adt - 1*eye(no_genes))*At*Bdt; %%% Bilinear transformation
for j=1:no_compounds
    ADJ=Adds(((j-1)*no_genes+1):(j*no_genes),:);
    CDJ=Cdds(((j-1)*no_genes+1):(j*no_genes),:);
    Ads(((j-1)*no_genes+1):(j*no_genes),:)=(2/(to_interpolate_dose(j,2)-
to_interpolate_dose(j,1)))*(ADJ-1*eye(no_genes))*pinv(ADJ+1*eye(no_genes)); %%% Bilinear
transformation
    Cs(((j-1)*no_genes+1):(j*no_genes),:)=pinv(ADJ - 1*eye(no_genes))*Ads(((j-
1)*no_genes+1):(j*no_genes),:)*CDJ;
end
```

DTNI.m

```
function [At,B,Ads,Cs,total_states,Kds] =
DTNI(data,time_points,doses,no_compounds,principal_components)

% This function is called by main.m and estimates the parameters
% At,B,Ads,Cs of the ODE.

% This code is adapted from the tsni.m code, published in
% the supplementary data of Bansal, M., Della Gatta G, di Bernardo D.(2006).
% Inference of gene regulatory networks and compound mode of action from
% time course gene expression profiles. Bioinformatics,22(7):815-822.

% input:
% data: gene expression data, average log2 ratios
%   rows=genes; column=samples
%   Columns have to be ordered as follows (for p compounds, q time points, r doses):
%   column 1: compound 1 at time 1 and dose 1
%   ...
%   column r: compound 1 at time 1 and dose r
%   column r+1: compound 1 at time 2 and dose 1
%   ...
```

```
%   column 2*r: compound 1 at time 2 and dose r
%   ...
%   column (q-1)*r+1: compound 1 at time q and dose 1
%   ...
%   column q*r: compound 1 at time q and dose r
%   column q*r + 1: compound 2 at time 1 and dose 1
%   ...
%   column 2*q*r: compound 2 at time q and dose r
%   ...
%   column (p-1)*q*r+1: compound p at time 1 and dose 1
%   ...
%   column p*q*r: compound p at time q and dose r
% time points: row vector of time points
% doses: row vector of doses, ordered as follows:
%   compound 1 dose 1, ..., compound 1 dose r, ... , compound p dose 1,
%   compound p dose r
% no_compounds: number of compounds in the data set
% principal components: selected number of PCs - put zero if you have no idea how many PCs to
keep

% output:
% At: interaction matrix (time Jacobian). It is a fully connected network.
% Ads: interaction matrices (dose Jacobians). Fully connected networks.
% B: dose dependence parameters for each gene.
% C: time dependence parameters for each gene.
% total_states: Number of principal components used.
% Kds: matrix containing dose interaction matrices and time dependence parameters in discrete
dose space

no_genes=size(data,1);
no_samples=size(data,2)/no_compounds;
no_doses=size(doses,2)/no_compounds;
p=size(time_points,2);

X=[];
for i=1:p
    for j=1:no_compounds
        X(((j-1)*no_genes+1):(j*no_genes),1:no_doses,i)=data(:,((j-1)*no_samples+(no_doses*i-
(no_doses-1))):((j-1)*no_samples+(no_doses*i)));
    end
end
s = [no_genes no_samples];

sliding_window_size = 4;
smoothing_parameter = 0.8;

x = [0 time_points];
for i=1:2*length(x)-1
    to_interpolate_time(i)=(i-1)*x(length(x))/(2*(length(x)-1));
end

y=[];
for j=1:no_compounds
    y(j,:) = [0 doses(((j-1)*no_doses+1):(j*no_doses))];
    v=y(j,:);
    for i=1:(2*length(v)-1)
        to_interpolate_dose(j,i)=(i-1)*v(length(v))/(2*(length(v)-1));
    end
end

count_polynomial_coefs_spline_time = 0;
for j = 1:length(to_interpolate_time)
    if to_interpolate_time(j) < (x(2) - x(1))
        count_polynomial_coefs_spline_time = count_polynomial_coefs_spline_time + 1;
    end
end

% smoothing of time series
for j=1:no_doses
clear dd sss sd
   for k=1:p
```

```
    Y(:,k)=X(:,j,k);
   end
   for i = 1:(no_genes*no_compounds)
        z = [0 Y(i,:)];
        [scss,polynomial_coefs_spline,confidence_lower,confidence_upper,smoothed_values] =
interpolation_data(x,z,smoothing_parameter,sliding_window_size); %%% Smoothing of data and
interpolating it.
        dd(i,1:count_polynomial_coefs_spline_time) =
polyval(polynomial_coefs_spline,to_interpolate_time(1:count_polynomial_coefs_spline_time));
        dd(i,count_polynomial_coefs_spline_time+1:length(to_interpolate_time)) =
fnval(scss,to_interpolate_time(count_polynomial_coefs_spline_time+1:length(to_interpolate_time)))
;
        sss(i,:) = confidence_upper - smoothed_values;
        sss(i,1) = 0;
        sd(i,:) = spline(x,sss(i,:),to_interpolate_time);
    end
    D(:,j,:)=dd;
end

% fitting dose series (Hermite interpolation)
for k=1:p
  clear dd sss sd
  for i = 1:(no_genes*no_compounds)
        z = [0 X(i,:,k)];
        w = floor((i-1)/no_genes)+1;
        yw=y(w,:);
        f = fit(yw',z','pchipinterp');
        dd(i,1:length(to_interpolate_dose(w,:))) = feval(f,to_interpolate_dose(w,:));
  end
  T(:,:,k)=dd;
end

[At,B,Ads,Cs,total_states,Kds] =
discretization_DTNI(D,T,no_compounds,doses,time_points,to_interpolate_time,to_interpolate_dose,pr
incipal_components);
```

---

perm_res.m

```
function
[pvalAt,pvalAds]=perm_res(data,At,Ads,no_compounds,residuals,model,time_points,doses,total_states
)

% This function is called by main.m and performs an adapted permutation test by permuting the
residuals 1000 times
% input:
% data: gene expression data, average log2 ratios
%   rows=genes; column=samples
%   Columns have to be ordered as follows (for p compounds, q time points, r doses):
%   column 1: compound 1 at time 1 and dose 1
%   ...
%   column r: compound 1 at time 1 and dose r
%   column r+1: compound 1 at time 2 and dose 1
%   ...
%   column 2*r: compound 1 at time 2 and dose r
%   ...
%   column (q-1)*r+1: compound 1 at time q and dose 1
%   ...
%   column q*r: compound 1 at time q and dose r
%   column q*r + 1: compound 2 at time 1 and dose 1
%   ...
%   column 2*q*r: compound 2 at time q and dose r
%   ...
%   column (p-1)*q*r+1: compound p at time 1 and dose 1
%   ...
%   column p*q*r: compound p at time q and dose r
% At,Ads: interaction matrices (output of DTNI.m)
```

```
% no_compounds: number of compounds in the data set
% residuals: residuals (output resid_model.m)
% model: estimation of the data from the model for the optimal number of PCs (output
resid_model.m)
% time points: row vector of time points
% doses: row vector of doses, ordered as follows:
%   compound 1 dose 1, ..., compound 1 dose r, ... , compound p dose 1,
%   compound p dose r
% total_states: Number of principal components used.

% output:
% pvalAt, pvalAds: p-values for the interactions

r=size(data,1);
s=no_compounds*r;
SIGAt=zeros(r,r);
SIGAds=zeros(s,r);
for i=1:1000
    i
    d(:,i)=randperm(size(residuals,2));
    RES=residuals(:,d(:,i));
    datasum=model+RES;
    no_genes=size(data,1);
    no_samples=size(datasum,2);
    for j=1:no_compounds
        dataperm(1:no_genes,((j-1)*no_samples+1):(j*no_samples))=datasum(((j-
1)*no_genes+1):(j*no_genes),:);
    end
    [At1,B1,Ads1,Cs1,total_states1,Kds1] =
DTNI(dataperm,time_points,doses,no_compounds,total_states)
    for j=1:r
        for k=1:r
            if abs(At1(j,k))>=abs(At(j,k))%compares real outcome with permuted outcome
                SIGAt(j,k)=SIGAt(j,k)+1;
            end
        end
    end
    for j=1:s
        for k=1:r
            if abs(Ads1(j,k))>=abs(Ads(j,k))%compares real outcome with permuted outcome
                SIGAds(j,k)=SIGAds(j,k)+1;
            end
        end
    end
save('workspace_DTNI')
end
pvalAt=(1/1000)*SIGAt;
pvalAds=(1/1000)*SIGAds;
save('workspace_DTNI')
```

----

resid_model.m

```
function [residuals,model] = resid_model(data,time_points,doses,Kd)

% This function is called by main.m and calculates the residuals and the
% model estimation (from ODE for dose) of the data.
% input:
% data: gene expression data, average log2 ratios
%   rows=genes; column=samples
%   Columns have to be ordered as follows (for p compounds, q time points, r doses):
%   column 1: compound 1 at time 1 and dose 1
%   ...
%   column r: compound 1 at time 1 and dose r
%   column r+1: compound 1 at time 2 and dose 1
%   ...
%   column 2*r: compound 1 at time 2 and dose r
```

```
%   ...
%   column (q-1)*r+1: compound 1 at time q and dose 1
%   ...
%   column q*r: compound 1 at time q and dose r
%   column q*r + 1: compound 2 at time 1 and dose 1
%   ...
%   column 2*q*r: compound 2 at time q and dose r
%   ...
%   column (p-1)*q*r+1: compound p at time 1 and dose 1
%   ...
%   column p*q*r: compound p at time q and dose r
% time points: row vector of time points
% doses: row vector of doses, ordered as follows:
%   compound 1 dose 1, ..., compound 1 dose r, ... , compound p dose 1,
%   compound p dose r
% Kds: matrix containing dose interaction matrices and time dependence parameters in discrete
dose space

p=size(time_points,2);
q=size(doses,2);
r=size(data,1);
M=[];
for j=1:p
    M(:,(j-1)*q+1)=[zeros(r,1);time_points(j)];
    for i=2:q
        M(:,(j-1)*q+i)=[data(:,(j-1)*q+i-1);time_points(j)];
    end
end
model=Kd*M;
residuals=data-model;
```

---

ssqmatrix.m

```
function SSQR = ssqmatrix(data,time_points,doses,no_compounds,no_genes,no_samples,no_doses)

% This function is called by main.m and determines the sum of squares of
% the residuals for each number of PCs.
% input:
% data: gene expression data, average log2 ratios
%   rows=genes; column=samples
%   Columns have to be ordered as follows (for p compounds, q time points, r doses):
%   column 1: compound 1 at time 1 and dose 1
%   ...
%   column r: compound 1 at time 1 and dose r
%   column r+1: compound 1 at time 2 and dose 1
%   ...
%   column 2*r: compound 1 at time 2 and dose r
%   ...
%   column (q-1)*r+1: compound 1 at time q and dose 1
%   ...
%   column q*r: compound 1 at time q and dose r
%   column q*r + 1: compound 2 at time 1 and dose 1
%   ...
%   column 2*q*r: compound 2 at time q and dose r
%   ...
%   column (p-1)*q*r+1: compound p at time 1 and dose 1
%   ...
%   column p*q*r: compound p at time q and dose r
% At,Ads: interaction matrices (output of DTNI.m)
% no_compounds: number of compounds in the data set
% residuals: residuals (output resid_model.m)
% model: estimation of the data from the model for the optimal number of PCs (output
resid_model.m)
% time points: row vector of time points
% doses: row vector of doses, ordered as follows:
%   compound 1 dose 1, ..., compound 1 dose r, ... , compound p dose 1,
```

```
%   compound p dose r
% no_compounds: number of compounds
% no_genes: number of genes
% no_samples: number of time points * number of doses per compound
% no_doses: number of doses per compound

for l=1:((no_compounds+1)*no_genes + 2*no_compounds)
    [At,B,Ads,Cs,total_states,Kds] = DTNI(data,time_points,doses,no_compounds,l);
    for j=1:no_compounds
        [residuals(((j-1)*no_genes+1):(j*no_genes),:),model(((j-1)*no_genes+1):(j*no_genes),:)] =
resid_model(data(:,((j-1)*no_samples+1):(j*no_samples)),time_points,doses(:,((j-
1)*no_doses+1):(j*no_doses)),Kds(((j-1)*no_genes+1):(j*no_genes),:));
    end
    SSQR(l)=ssq(residuals);
    save('workspace_DTNI')
end
```

References

Shannon P, Markiel A, Ozier O, et al. (2003) Cytoscape: a software environment for integrated models of biomolecular interaction networks. Genome Res 13(11):2498-504
