# Supplementary material for: DTNI: a novel toxicogenomics data analysis tool for identifying the molecular mechanisms underlying the adverse effects of toxic compounds
Source: Arch Toxicol. 2016 Dec 28;91(6):2343–52. doi: 10.1007/s00204-016-1922-5 (PMC5429357; doi:10.1007/s00204-016-1922-5)
Supplement: Supplementary file 4 — Supplementary material 4 (PDF 948 kb) [file 204_2016_1922_MOESM4_ESM.pdf]

Supplementary Material 3

Article title: DTNI: a novel toxicogenomics data analysis tool for identifying the molecular mechanisms underlying the adverse effects of toxic compounds

Journal name: Archives of Toxicology

Authors names: Diana M. Hendrickx[1], Terezinha Souza[1], Danyel G. J. Jennen[1], Jos C. S. Kleinjans[1]

Affiliation: [1] Department of Toxicogenomics, GROW-School for Oncology and Developmental Biology, Maastricht University, Universiteitssingel 40, 6229 ER Maastricht, The Netherlands. Postal address: P.O. Box 616, 6200 MD Maastricht, The Netherlands. Telephone: +31 43 3881845.

E-mail address of the corresponding author: Diana M. Hendrickx, d.hendrickx@maastrichtuniversity.nl

Model validation – additional information

Simulations

Table S3_1: Drug reactions added to the simulation model

Name	Reaction	Rate Law
Reaction for drug A	A + IKK = IKK_A	Mass action (reversible)
Reaction for drug B	B + NFkB = NFkB_B	Mass action (reversible)
Reaction for drug C	C + IkBalpha = IkBalpha_C	Mass action (reversible)
Reaction for drug D	D + IkBbeta = IkBbeta_D	Mass action (reversible)
Reaction for drug E	E + IkBeps = IkBeps_E	Mass action (reversible)
Reaction for drug F	F + IkBalpha_NFkB = IkBalpha_NFkB_F	Mass action (reversible)

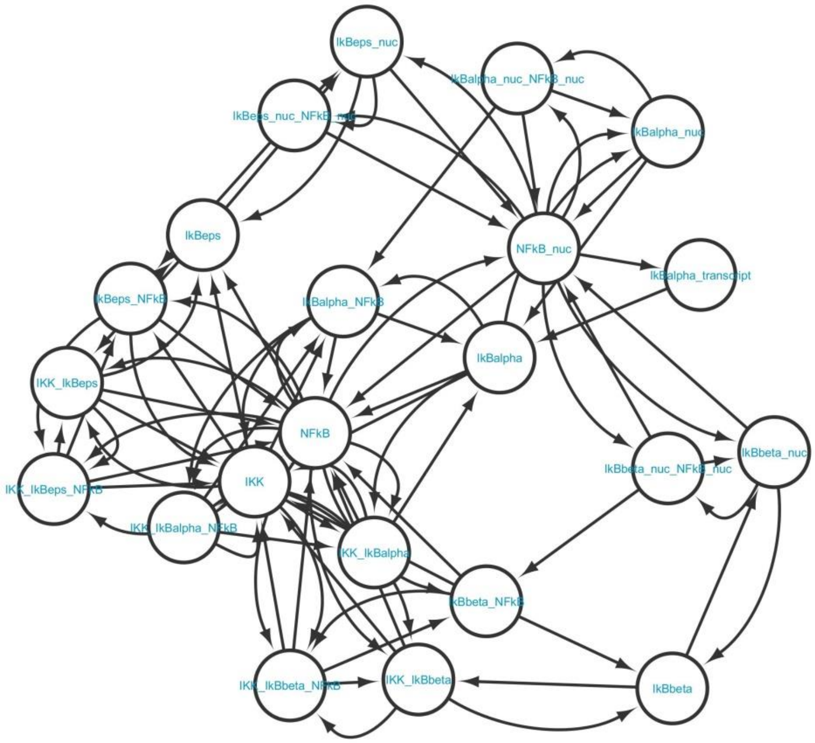

Figure S3-1: Interaction network for the simulation model

Table S3_2: Overview of the simulations performed in this study.

Goal	Number of time points	Number of doses	Number of compounds	Noise level
Compare DTNI with TSNI	3	3	1	---
Assess the influence of the number of time points	3, 7, 8, 12, 48, 72, 144, 288	3	1	---
Assess the influence of the number of doses	3	3, 6, 10, 14, 34	1	---
Assess the influence of the number of compounds	3	3	1, 2, 3, 4, 5, 6	---
Assess the influence of noise	3	3	1	10 data sets with 15% noise without replicates;<br>5 data sets with 15% noise with 2 replicates.
Assess the influence of LOOCV	3	3	6	---

Table S3_3: Initial values for the variables in the model, used for all simulations.

Name	Initial value
IkBalpha	1
NFkB	1
IkBalpha_NFkB	0
IkBbeta	1
IkBbeta_NFkB	0
IkBeps	1
IkBeps_NFkB	0
IKK_IkBalpha	0
IKK_IkBalpha_NFkB	0
IKK	1
IKK_IkBbeta	0
IKK_IkBbeta_NFkB	0
IKK_IkBeps	0
IKK_IkBeps_NFkB	0
IKK_A	0
NFkB_B	0
IKBalpha_C	0
IkBbeta_D	0
IkBeps_E	0
IkBalpha_NFkB_F	0
NFkB_nuc	0
IkBalpha_nuc	0
IkBalpha_nuc_NFkB_nuc	0
IkBbeta_nuc	0
IkBbeta_nuc_NFkB_nuc	0
IkBeps_nuc	0
IkBalpha_transcript	0
IkBbeta_transcript	0
IkBeps_transcript	0
IkBeps_nuc_NFkB_nuc	0

Table S3_4: Values of the doses (μmol/l) of the drugs for each simulation

Simulation	doses
compare DTNI with TSNI	drug A: 16, 80, 400; other drugs: dose = 0
influence of the number of time points	drug A: 16, 80, 400; other drugs: dose = 0
influence of the number of doses – 3 doses	drug A: 16, 80, 400; other drugs: dose = 0
influence of the number of doses – 6 doses	drug A: 16, 80, 200, 266.7, 333.3, 400; other drugs: dose = 0
influence of the number of doses – 10 doses	drug A: 10, 16, 20, 66.7, 80, 133.3, 200, 266.7, 333.3, 400; other drugs = 0
influence of the number of doses – 14 doses	drug A: 10, 16, 20, 30, 40, 50, 60, 66.7, 80, 133.3, 200, 266.7, 333.3, 400;<br>other drugs = 0
influence of the number of doses – 34 doses	drug A: 10, 16, 20, 30, 40, 50, 60, 66.7, 70, 80, 90, 100, 110, 120, 130, 133.3, 140, 150,<br>160, 170, 180, 190, 200, 210, 220, 230, 240, 250, 260, 266.7, 270, 280, 333.3, 400<br>other drugs = 0
influence of the number of compounds – 1 compound	drug A: 16, 80, 400; other drugs: dose = 0
influence of the number of compounds – 2 compounds	data set 1: drug A: 16, 80, 400; other drugs: dose = 0<br>data set 2: drug B: 16, 80, 400; other drugs: dose = 0
influence of the number of compounds – 3 compounds	data set 1: drug A: 16, 80, 400; other drugs: dose = 0<br>data set 2: drug B: 16, 80, 400; other drugs: dose = 0<br>data set 3: drug C: 16, 80, 400; other drugs: dose = 0
influence of the number of compounds – 4 compounds	data set 1: drug A: 16, 80, 400; other drugs: dose = 0<br>data set 2: drug B: 16, 80, 400; other drugs: dose = 0<br>data set 3: drug C: 16, 80, 400; other drugs: dose = 0<br>data set 4: drug D: 16, 80, 400; other drugs: dose = 0
influence of the number of compounds – 5 compounds	data set 1: drug A: 16, 80, 400; other drugs: dose = 0<br>data set 2: drug B: 16, 80, 400; other drugs: dose = 0<br>data set 3: drug C: 16, 80, 400; other drugs: dose = 0<br>data set 4: drug D: 16, 80, 400; other drugs: dose = 0<br>data set 5: drug E: 16, 80, 400; other drugs: dose = 0
influence of the number of compounds – 6 compounds	data set 1: drug A: 16, 80, 400; other drugs: dose = 0<br>data set 2: drug B: 16, 80, 400; other drugs: dose = 0<br>data set 3: drug C: 16, 80, 400; other drugs: dose = 0<br>data set 4: drug D: 16, 80, 400; other drugs: dose = 0<br>data set 5: drug E: 16, 80, 400; other drugs: dose = 0<br>data set 6: drug F: 16, 80, 400; other drugs: dose = 0
influence of noise	drug A: 16, 80, 400; other drugs: dose = 0
influence of LOOCV	data set 1: drug A: 16, 80, 400; other drugs: dose = 0<br>data set 2: drug B: 16, 80, 400; other drugs: dose = 0<br>data set 3: drug C: 16, 80, 400; other drugs: dose = 0<br>data set 4: drug D: 16, 80, 400; other drugs: dose = 0<br>data set 5: drug E: 16, 80, 400; other drugs: dose = 0<br>data set 6: drug F: 16, 80, 400; other drugs: dose = 0

Table S3_5: Influence of the number of time points – values of the time points in the simulated data sets.

simulation	time points
3 time points	2, 8, 24 hours
7 time points	1, 2, 5, 8, 16, 20, 24 hours
8 time points	1, 2, 5, 8, 12, 16, 20, 24 hours
12 time points	0.5, 1, 1.5, 2, 3.5, 5, 6.5, 8, 12, 16, 20, 24 hours
48 time points	from 0.5 to 24 hours, each half hour
72 time points	from 20 min to 24 hours, each 20 min
144 time points	from 10 min to 24 hours, each 10 min
288 time points	from 5 min to 24 hours, each 5 min

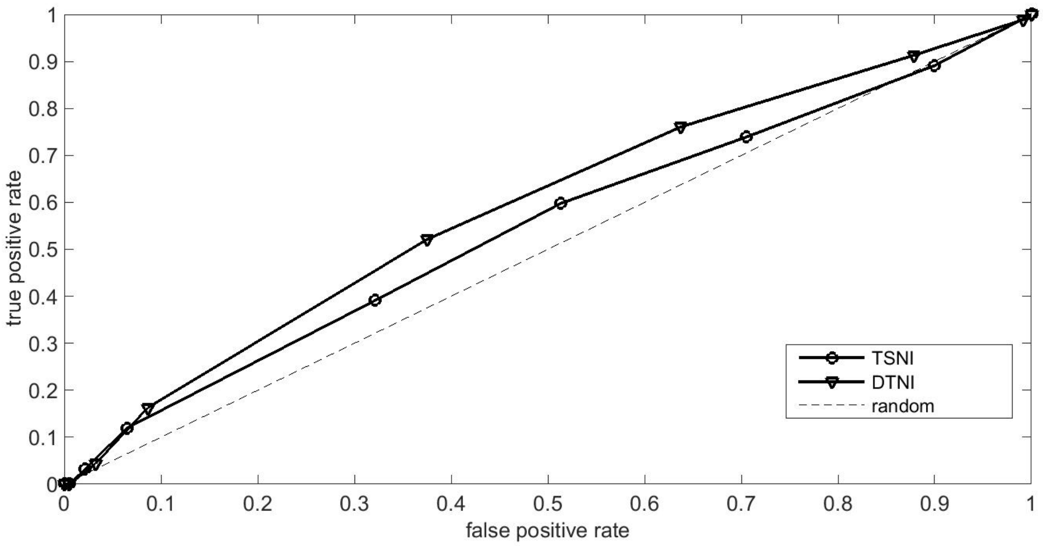

Figure S3-2: Comparison of DTNI and TSNI for simulated data of a single compound with three time points (2h, 8h, 24h), three doses (low, middle, high) and time-matched controls (dose = 0). ROC-curve for TSNI (circles) and DTNI (triangles).

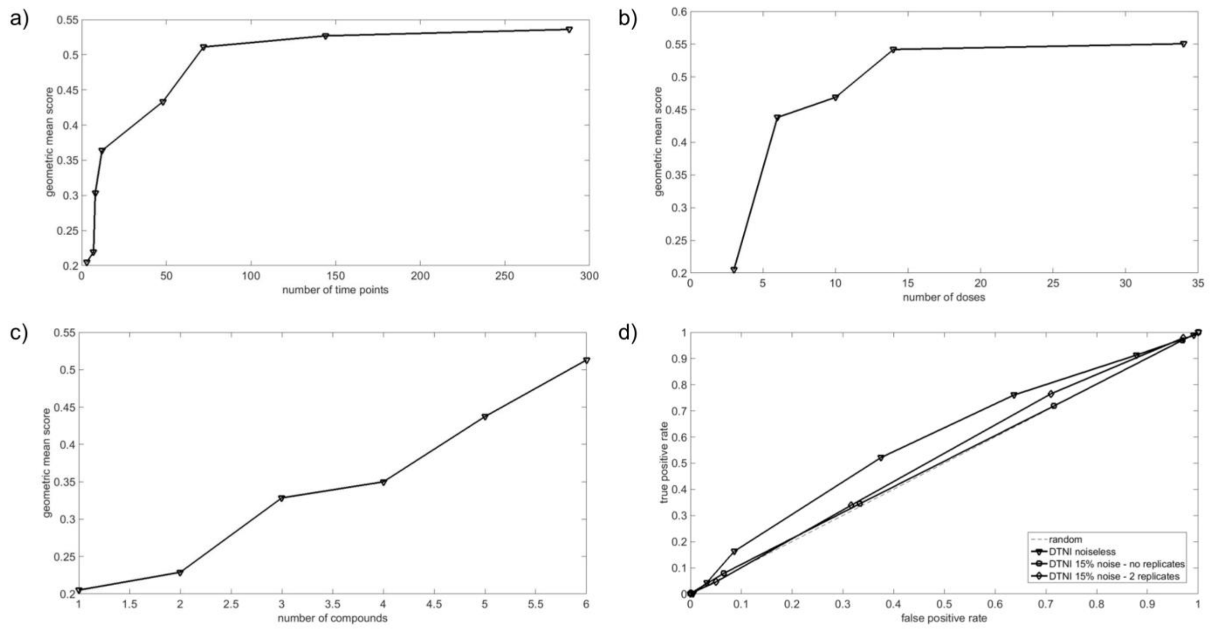

Figure S3-3: a) Influence of the number of time points on the performance of DTNI for simulated data of a single compound with three doses (low, middle, high) and time-matched controls (dose = 0). Plot of geometric mean score versus number of time points for a p-value threshold of 0.05. b) Influence of the number of doses on the performance of DTNI for simulated data of a single compound with three time points (2h, 8h, 24h) and time-matched controls (dose = 0). Plot of geometric mean score versus number of doses for a p-value threshold of 0.05. c) Influence of the number of compounds on the performance of DTNI for simulated data with three time points (2h, 8h, 24h), three doses (low, middle, high) and time-matched controls (dose = 0). Plot of geometric mean score versus number of compounds for a p-value threshold of 0.05. d) Influence of noise on the performance of DTNI for simulated data of a single compound with three time points (2h, 8h, 24h), three doses (low, middle, high) and time-matched controls (dose = 0). ▽: ROC curve for noiseless simulated data; □: average ROC curve for 10 simulated data sets with 15% noise and no replicates; ◊: average ROC curve for 5 simulated data sets with 15% noise and two replicates.

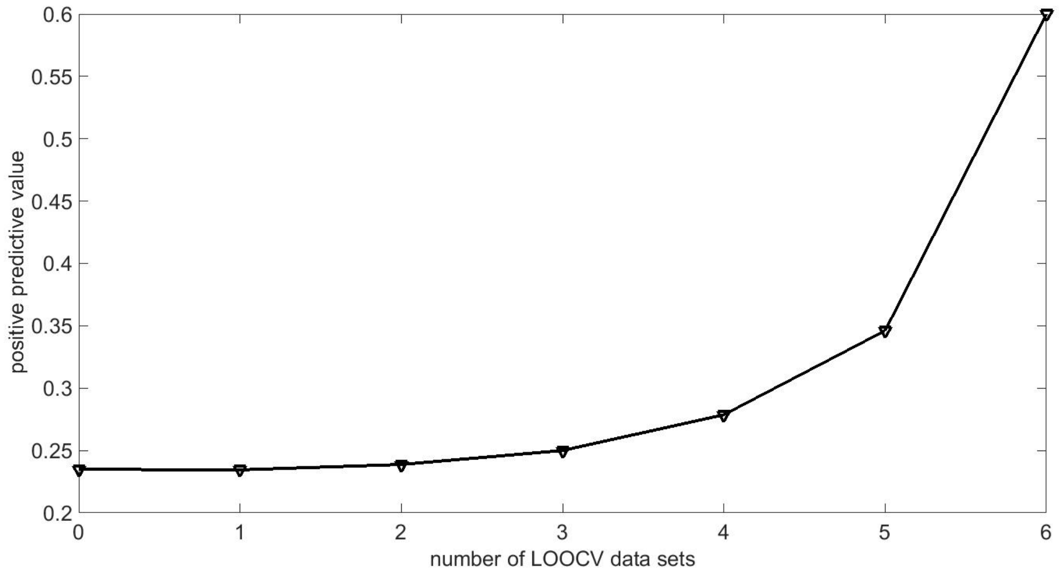

Figure S3-4: Influence of LOOCV on the performance of DTNI for simulated data of 6 compounds with three time points (2h, 8h, 24h), three doses (low, middle, high) and time-matched controls (dose = 0). Plot of positive predictive value versus number of LOOCV data sets for a p-value threshold of 0.05.

Validation on real experiments – PPAR signalling pathway

Peroxisome proliferator-activated receptor (PPAR) signalling is known to play an important role in drug-induced liver injury, in particular liver steatosis (Browning and Horton 2004, Tsakovska et al. 2014).
The PPAR signalling pathway was extracted from KEGG (http://www.genome.jp/kegg/)(Kanehisa et al. 2016) and consists of 72 genes, of which 67 were present in TG-GATEs.
Based on information from text-mining, extracted from the Comparative Toxicogenomics Database (http://ctdbase.org/) (Davis et al. 2015), we looked up the 4 compounds in TG-GATEs (http://toxico.nibiohn.go.jp/) (Igarashi et al. 2015) affecting the largest number of genes of the PPAR signalling pathway. These compounds are clofibrate (affecting 51 genes of the PPAR signalling pathway), thioacetamide (35 genes), tetracycline (30 genes) and amiodarone (28 genes). We studied how DTNI performs: (1) when inferring the subnetwork of the PPAR signalling pathway affected by clofibrate only; (2) when inferring the subnetwork of the PPAR signalling pathway affected by the four compounds, using LOOCV.
The resulting subnetwork was further validated by means of CPDB induced network modules analysis, and connections are classified using the following definitions: (i) a true positive interaction (TP): a gene pair linked in CPDB by direct interaction or via biological variables not in the analysed gene list (pathway) that is predicted by DTNI as a directly interacting gene pair; (ii) a false positive interaction (FP): a gene pair only linked in CPDB via genes in the analysed gene list (pathway) that is predicted by DTNI as a directly interacting gene pair; (iii) a false negative (false absent interaction): a direct interaction in CPDB that is not predicted by DTNI; (iv) a true negative (true absent interaction): absence of a direct interaction correctly identified.
The following performance measures were used to check out the inferred networks: (i) sensitivity (true positive rate (TPR)): the proportion of directly interacting gene pairs that are inferred as such; (ii) specificity (true negative rate (TNR)): the proportion of not directly interacting gene pairs that are inferred as such; (iii) geometric mean score (geometric mean of the sensitivity and the specificity), a larger geometric mean score means a better prediction of the toxicant(s)-induced GRN.
Table S3-6 shows the results of the analysis.

Table S3-6: Results of DTNI analysis of the genes of the PPAR signalling pathway

Experiment	Number of nodes	Number of edges	Sensitivity (TPR)	Specificity (TNR)	Geometric mean score
Clofibrate only	58	239	0.50	0.94	0.6860
Four compounds + LOOCV	12	6	0.50	0.96	0.6933

For the subnetwork of the PPAR signalling pathway affected by clofibrate only, we checked if the predicted network contains key genes for compound reactions by comparing the genes in the network with information on the effect of fibrates on PPAR alpha, available from Fruchart 2001. Table S3-7 shows that the network indeed contains key genes for compound reactions, but also many other genes, of which most of them were already related before to clofibrate exposure in previous studies and included in the Comparative Toxicogenomics Database (CTD) (http://ctdbase.org/) (Davis et al. 2015).

Table S3-7: Comparison of genes in the inferred network for clofibrate with prior information in CTD and Fruchart et al.

Gene	CTD	Fruchart et al
ACAA1	Yes	---
ACADL	Yes	---
ACADM	Yes	---
ACOX1	Yes	---
ACOX3	---	---
ACSBG1	---	---
ACSL1	Yes	---
ACSL3	---	---
ACSL5	Yes	---
ADIPOQ	---	---
ANGPTL4	Yes	---
APOA1	Yes	Key gene (high-density lipid metabolism)
APOA2	---	Key gene (high-density lipid metabolism)
APOA5	Yes	---
APOC3	Yes	---
CD36	Yes	---
CPT1C	---	---
CPT2	Yes	---
CYP27A1	---	---
CYP7A1	Yes	---
CYP8B1	Yes	---
DBI	Yes	---
EHHADH	Yes	---
FABP2	Yes	---
FABP3	Yes	---
FABP4	Yes	---
FABP6	---	---
FABP7	Yes	---
FADS2	Yes	---
GK	---	---
HMGCS2	Yes	---
ILK	---	---
LPL	Yes	Key gene (high-density lipid metabolism)
ME1	Yes	---
MMP1	Yes	---
NR1H3	Yes	---
OLR1	Yes	---
PCK1	Yes	---
PDPK1	---	---
PLIN1	---	---
PLIN2	---	---
PLIN4	---	---
PLTP	Yes	---
PPARA	Yes	Directly affected by fibrates
PPARD	Yes	Fibrates induce transcription of PPARs
PPARG	Yes	Fibrates induce transcription of PPARs
RXRA	Yes	Fibrates influence RXRs
RXRB	Yes	Fibrates influence RXRs
RXRG	---	Fibrates influence RXRs
SCD	Yes	---
SCD5	---	---
SCP2	Yes	---
SLC27A1	Yes	---
SLC27A2	Yes	---
SLC27A4	Yes	---
SLC27A6	---	---
SORBS1	---	---
UCP1	Yes	---

References

Browning JD, Horton JD (2004) Molecular mediators of hepatic steatosis and liver injury. J Clin Invest 114(2):147-152

Davis AP, Grondin CJ, Lennon-Hopkins K, et al. (2015) The Comparative Toxicogenomics Database's 10th year anniversary: update 2015. Nucleic Acids Res 43(Database issue):D914-20

Fruchart JC (2001) Peroxisome Proliferator–Activated Receptor-alpha Activation and High-Density Lipoprotein Metabolism. Am J Cardiol, 88(suppl):24N-29N.

Igarashi Y, Nakatsu N, Yamashita T, et al. (2015) Open TG-GATEs: a large-scale toxicogenomics database. Nucleic Acids Res 43(Database issue):D921-7

Kanehisa M, Sato Y, Kawashima M, Furumichi M, Tanabe M (2016) KEGG as a reference resource for gene and protein annotation. Nucleic Acids Res 44(D1):D457-62

Tsakovska, I, Al Shariff M, Alov P, et al. (2014) Molecular Modelling Study of the PPARalpha Receptor in Relation to the Mode of Action/Adverse Outcome Pathway Framework for Liver Steatosis. Int J Mol Sci 15(5):7651-7666
