# Supplementary material for: DTNI: a novel toxicogenomics data analysis tool for identifying the molecular mechanisms underlying the adverse effects of toxic compounds
Source: Arch Toxicol. 2016 Dec 28;91(6):2343–52. doi: 10.1007/s00204-016-1922-5 (PMC5429357; doi:10.1007/s00204-016-1922-5)
Supplement: Supplementary file 5 — Supplementary material 5 (PDF 734 kb) [file 204_2016_1922_MOESM5_ESM.pdf]

Supplementary Material 4

Article title: DTNI: a novel toxicogenomics data analysis tool for identifying the molecular mechanisms underlying the adverse effects of toxic compounds

Journal name: Archives of Toxicology

Authors names: Diana M. Hendrickx[1], Terezinha Souza[1], Danyel G. J. Jennen[1], Jos C. S. Kleinjans[1]

Affiliation: [1] Department of Toxicogenomics, GROW-School for Oncology and Developmental Biology, Maastricht University, Universiteitssingel 40, 6229 ER Maastricht, The Netherlands. Postal address: P.O. Box 616, 6200 MD Maastricht, The Netherlands. Telephone: +31 43 3881845.

E-mail address of the corresponding author: Diana M. Hendrickx, d.hendrickx@maastrichtuniversity.nl

Example 1: NF-kB pathway – additional information

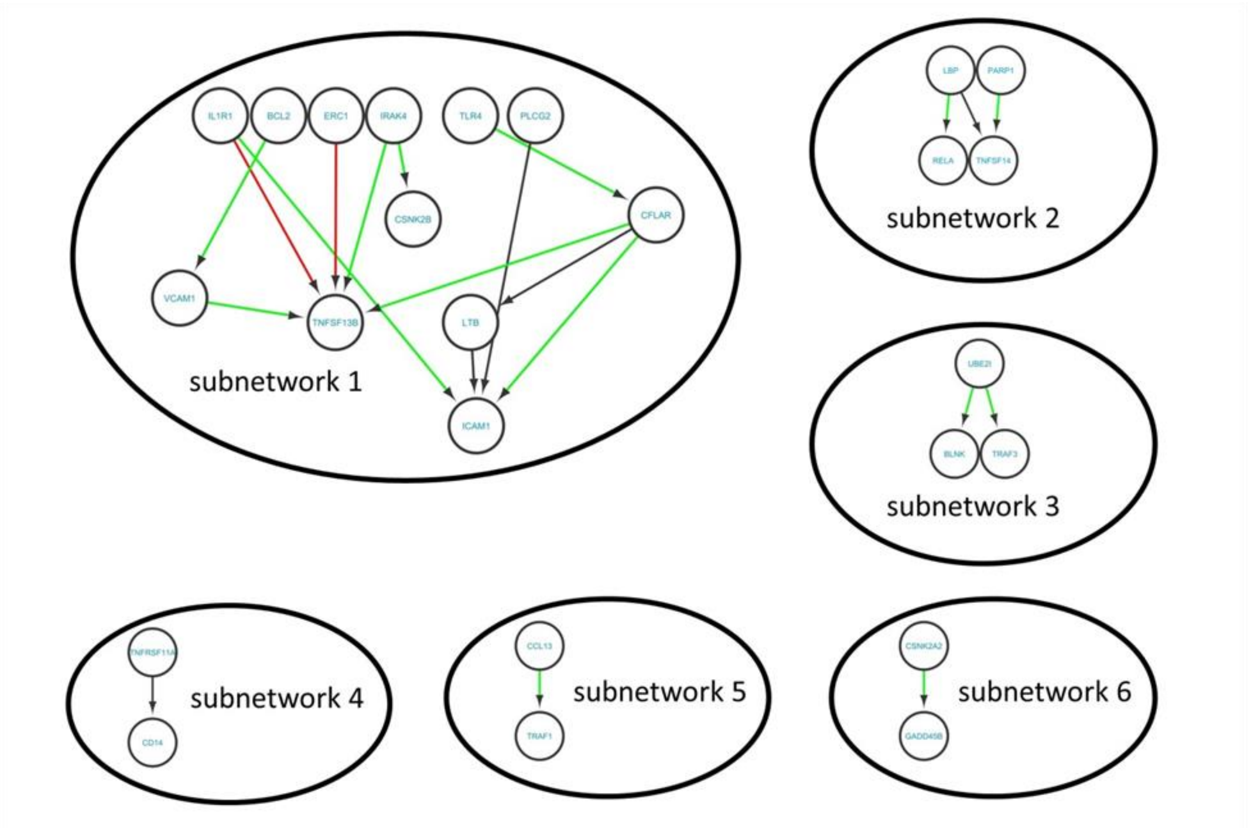

Figure S4-1: NF-kB pathway - interaction network inferred with DTNI for $p \leq 0.05$. Green edges: true positives (TP), red edges: false positives (FP), black edges: novel interactions. Subnetworks within the inferred network are indicated with ellipses.

Table S4-1: Details of the edges in the network of Figure S4-1. PI = protein interaction; BI = biochemical interaction.

From (EntrezID)	To (EntrezID)	From (name)	To (name)	Interaction	Intermediate	Intermediate type	Interaction type; gene regulatory and …	Intermediates (EntrezID)	In gene list?	Conclusion
7329	7187	UBE2I	TRAF3	direct			PI			TP
8837	4050	CFLAR	LTB	none						novel
6357	7185	CCL13	TRAF1	indirect	BCL6	gene/protein	PI	604	no	TP
3554	3383	IL1R1	ICAM1	indirect	GlcNAc	compound	PI		no	TP
8837	10673	CFLAR	TNFSF13B	indirect	CASP8	gene/protein	PI and BI	841	no	TP
					MAP3K14			9020	yes	
					TRAF3			7187	yes	
5336	3383	PLCG2	ICAM1	none						novel
7412	10673	VCAM1	TNFSF13B	indirect	HEATR1	gene/protein	PI	55127	no	TP
					ITGB1			3688	no	
8792	929	TNFRSF11A	CD14	none						novel
1459	4616	CSNK2A2	GADD45B	indirect	EGR1	gene/protein	PI and BI	1958	no	TP
142	8740	PARP1	TNFSF14	indirect	TRAF2	gene/protein	PI and BI	7186	no	TP
					BIRC2			329	no	
7329	29760	UBE2I	BLNK	indirect	AR	gene/protein	PI and BI	367	no	TP
					CD2AP			23607	no	
					SH3KBP1			30011	no	
					p-7Y-KIT			3815	no	
51135	1460	IRAK4	CSNK2B	indirect	PDS5A	gene/protein	PI and BI	23244	no	TP
					VRK3			51231	no	
					p-6S-NCF1			653361	no	
					MAST3			23031	no	
					BRSK2			9024	no	
					BRSK1			84446	no	
					RPS6KB2			6199	no	
3929	5970	LBP	RELA	indirect	CEBPB	gene/protein	PI	1051	no	TP
23085	10673	ERC1	TNFSF13B	indirect	MAP3K7	gene/protein	PI and BI	6885	yes	FP
3554	10673	IL1R1	TNFSF13B	indirect	MAP3K14	gene/protein	PI and BI	9020	yes	FP
4050	3383	LTB	ICAM1	none						novel
51135	10673	IRAK4	TNFSF13B	indirect	NF1(2-2839)	gene/protein	PI	4763	no	TP
					TAC1			23495	no	
596	7412	BCL2	VCAM1	indirect	NFkB.complex	complex	PI		no	TP
					SF1	gene/protein		7536	no	
7099	8837	TLR4	CFLAR	indirect	STUB1	gene/protein	PI	10273	no	TP
					SPI1			6688	no	
					TRAF3			7187	yes	
					TICAM1			148022	yes	
8837	3383	CFLAR	ICAM1	indirect	NF-kB complex	complex	PI and BI		no	TP
					EIF3F	gene/protein		8665	no	
3929	8740	LBP	TNFSF14	none						novel

Table S4-2: Pathways (q-value ≤ 0.05) in ConsensusPathDB related to the network in Figure S4-1. Disease pathways of non-liver diseases were removed from the table.

pathway	database	q-value
NF-kappa B signaling pathway - Homo sapiens (human)	KEGG	2.44E-51
Immune System	Reactome	1.87E-08
Cytokine Signaling in Immune system	Reactome	0.00000216
Innate Immune System	Reactome	0.000339
Toll Like Receptor 4 (TLR4) Cascade	Reactome	0.000000304
Toll-Like Receptors Cascades	Reactome	0.000000611
RANKL-RANK Signaling Pathway	Wikipathways	0.000000175
TLR NFkB	INOH	0.000000304
Toll-like receptor signaling pathway	Wikipathways	0.00000183
Toll-like receptor signaling pathway - Homo sapiens (human)	KEGG	0.00000183
TNF signaling pathway - Homo sapiens (human)	KEGG	0.00000316
Regulation of toll-like receptor signaling pathway	Wikipathways	0.00000917
Cytokine-cytokine receptor interaction - Homo sapiens (human)	KEGG	0.000145
TNF receptor superfamily (TNFSF) members mediating non-canonical NF-kB pathway	Reactome	0.000000029
TLR ECSIT MEKK1 JNK	INOH	0.000000182
TNFR2 non-canonical NF-kB pathway	Reactome	0.00000146
hiv-1 nef: negative effector of fas and tnf	BioCarta	0.00000246
Apoptosis	Wikipathways	0.0000158
Apoptosis - Homo sapiens (human)	KEGG	0.0000176
Corticotropin-releasing hormone	Wikipathways	0.000024
Activated TLR4 signalling	Reactome	0.0000398
TNFalpha	NetPath	0.00062
Pathways in cancer - Homo sapiens (human)	KEGG	0.00546
TLR ECSIT MEKK1 p38	INOH	0.00000358
IL1-mediated signaling events	PID	0.0000145
HIV-1 Nef: Negative effector of Fas and TNF-alpha	PID	0.0000158
TLR JNK	INOH	0.0000897
TLR p38	INOH	0.0000922
MyD88:Mal cascade initiated on plasma membrane	Reactome	0.000213
Toll Like Receptor TLR1:TLR2 Cascade	Reactome	0.000213
Toll Like Receptor TLR6:TLR2 Cascade	Reactome	0.000213
Toll Like Receptor 2 (TLR2) Cascade	Reactome	0.000213
Apoptosis Modulation and Signaling	Wikipathways	0.000271
TRIF-mediated TLR3/TLR4 signaling	Reactome	0.000271
MyD88-independent TLR3/TLR4 cascade	Reactome	0.000271
Toll Like Receptor 3 (TLR3) Cascade	Reactome	0.000271
HIF-1 signaling pathway - Homo sapiens (human)	KEGG	0.00039
Apoptosis	Reactome	0.000483
Programmed Cell Death	Reactome	0.000512
Neurotrophin signaling pathway - Homo sapiens (human)	KEGG	0.000602
MAPK signaling pathway - Homo sapiens (human)	KEGG	0.00649
Signaling by Interleukins	Reactome	0.00762
tnfr2 signaling pathway	BioCarta	0.0000257
Activation of IRF3/IRF7 mediated by TBK1/IKK epsilon	Reactome	0.0000309
Ligand-dependent caspase activation	Reactome	0.0000525
nf-kb signaling pathway	BioCarta	0.0000922
Endogenous TLR signaling	PID	0.000104
RANKL	NetPath	0.000134
Caspase activation via extrinsic apoptotic signalig pathway	Reactome	0.000181
TWEAK	NetPath	0.000213
toll-like receptor pathway	BioCarta	0.000312
CD40/CD40L signaling	PID	0.000312
inactivation of gsk3 by akt causes accumulation of b-catenin in alveolar macrophages	BioCarta	0.000407
FAS pathway and Stress induction of HSP regulation	Wikipathways	0.000483
TWEAK Signaling Pathway	Wikipathways	0.000507
Interleukin-11 Signaling Pathway	Wikipathways	0.000519
Structural Pathway of Interleukin 1 (IL-1)	Wikipathways	0.00054
keratinocyte differentiation	BioCarta	0.000805
IL-1 signaling pathway	Wikipathways	0.00083
IL1	NetPath	0.00083
IL12-mediated signaling events	PID	0.0011
IL-1 NFkB	INOH	0.0012
BCR signaling pathway	PID	0.00147
B cell receptor signaling pathway - Homo sapiens (human)	KEGG	0.00176
B cell receptor signaling	INOH	0.0032

B Cell Receptor Signaling Pathway	Wikipathways	0.00386
Leukocyte transendothelial migration - Homo sapiens (human)	KEGG	0.00546
BCR	NetPath	0.00718
Hepatitis B - Homo sapiens (human)	KEGG	0.00897
MicroRNAs in cancer - Homo sapiens (human)	KEGG	0.0103
Signaling by the B Cell Receptor (BCR)	Reactome	0.0109
Transcriptional misregulation in cancer - Homo sapiens (human)	KEGG	0.0132
Transfer of LPS from LBP carrier to CD14	Reactome	0.0000435
yaci and bcma stimulation of b cell immune responses	BioCarta	0.000446
WNT mediated activation of DVL	Reactome	0.000522
TRIF-mediated programmed cell death	Reactome	0.00062
cd40l signaling pathway	BioCarta	0.00062
TRAF6 mediated induction of TAK1 complex	Reactome	0.000866
Condensation of Prometaphase Chromosomes	Reactome	0.000866
Signal transduction by L1	Reactome	0.00265
EBV LMP1 signaling	Wikipathways	0.00265
IKK complex recruitment mediated by RIP1	Reactome	0.00314
Canonical NF-kappaB pathway	PID	0.00314
TNFs bind their physiological receptors	Reactome	0.00336
IL1 and megakaryotyces in obesity	Wikipathways	0.00362
Beta2 integrin cell surface interactions	PID	0.00482
IL17 signaling pathway	Wikipathways	0.00546
ceramide signaling pathway	BioCarta	0.00591
Alpha4 beta1 integrin signaling events	PID	0.00591
EPO signaling pathway	PID	0.00622
signal transduction through il1r	BioCarta	0.00673
CRH	NetPath	0.00673
IL2 signaling events mediated by PI3K	PID	0.00737
Antigen activates B Cell Receptor (BCR) leading to generation of second messengers	Reactome	0.00762
Interleukin-1 signaling	Reactome	0.00835
CLEC7A (Dectin-1) signaling	Reactome	0.00869
TNF receptor signaling pathway	PID	0.00972
RAGE	NetPath	0.00972
Class I PI3K signaling events	PID	0.0101
Vitamin B12 Metabolism	Wikipathways	0.0103
Ceramide signaling pathway	PID	0.0109
RIG-I/MDA5 mediated induction of IFN-alpha/beta pathways	Reactome	0.0121
Signaling events mediated by HDAC Class I	PID	0.0138
Caspase Cascade in Apoptosis	PID	0.014
Folate Metabolism	Wikipathways	0.014
Thromboxane A2 receptor signaling	PID	0.014

Table S4-3: Pathways (q-value ≤ 0.05) in ConsensusPathDB related to the subnetworks in Figure S4-1. Disease pathways of non-liver diseases were removed from the table.

subnetwork	pathway	database	q-value
1	NF-kappa B signaling pathway - Homo sapiens (human)	KEGG	3.45E-24
	Immune System	Reactome	0.00000675
	Apoptosis - Homo sapiens (human)	KEGG	0.000031
	TLR ECSIT MEKK1 JNK	INOH	0.0000354
	IL1-mediated signaling events	PID	0.00009
	TLR NFkB	INOH	0.000435
	Apoptosis Modulation and Signaling	Wikipathways	0.000821
	Corticotropin-releasing hormone	Wikipathways	0.000821
	TNF receptor superfamily (TNFSF) members mediating non-canonical NF-kB pathway	Reactome	0.000887
	Ligand-dependent caspase activation	Reactome	0.000887
	HIF-1 signaling pathway - Homo sapiens (human)	KEGG	0.000887
	TNF signaling pathway - Homo sapiens (human)	KEGG	0.000903
	Apoptosis	Reactome	0.000903
	Programmed Cell Death	Reactome	0.000903
	nf-kb signaling pathway	BioCarta	0.000903
	Leukocyte transendothelial migration - Homo sapiens (human)	KEGG	0.000903
	Toll Like Receptor 4 (TLR4) Cascade	Reactome	0.000903
	Neurotrophin signaling pathway - Homo sapiens (human)	KEGG	0.000903
	TLR ECSIT MEKK1 p38	INOH	0.000926
	IL1 and megakaryotyces in obesity	Wikipathways	0.000971
	Endogenous TLR signaling	PID	0.00107
	Caspase activation via extrinsic apoptotic signaling pathway	Reactome	0.00109
	Toll-Like Receptors Cascades	Reactome	0.00109
	Beta2 integrin cell surface interactions	PID	0.00128
	Cytokine Signaling in Immune system	Reactome	0.00136
	EPO signaling pathway	PID	0.00146
	HIV-1 Nef: Negative effector of Fas and TNF-alpha	PID	0.00154
	FAS pathway and Stress induction of HSP regulation	Wikipathways	0.00209
	Interleukin-1 signaling	Reactome	0.00209
	Interleukin-11 Signaling Pathway	Wikipathways	0.00209
	Structural Pathway of Interleukin 1 (IL-1)	Wikipathways	0.00209
	TNFR2 non-canonical NF-kB pathway	Reactome	0.00222
	hiv-1 nef: negative effector of fas and tnf	BioCarta	0.00288
	IL-1 signaling pathway	Wikipathways	0.0029
	RANKL-RANK Signaling Pathway	Wikipathways	0.0029
	IL1	NetPath	0.0029
	Thromboxane A2 receptor signaling	PID	0.00294
	IL-1 JNK	INOH	0.00356
	TLR JNK	INOH	0.00356
	TLR p38	INOH	0.0036
	IL-1 NFkB	INOH	0.00364
	Integrin cell surface interactions	Reactome	0.00368
	IL-1 p38	INOH	0.00372
	Cytokine-cytokine receptor interaction - Homo sapiens (human)	KEGG	0.00394
	Validated targets of C-MYC transcriptional repression	PID	0.00421
	Apoptosis	Wikipathways	0.00511
	MyD88:Mal cascade initiated on plasma membrane	Reactome	0.00511
	Toll Like Receptor TLR1:TLR2 Cascade	Reactome	0.00511
	Toll Like Receptor TLR6:TLR2 Cascade	Reactome	0.00511
	Toll Like Receptor 2 (TLR2) Cascade	Reactome	0.00511
	Inflammatory mediator regulation of TRP channels - Homo sapiens (human)	KEGG	0.00663
	Toll-like receptor signaling pathway	Wikipathways	0.00684
	Innate Immune System	Reactome	0.00684
	Toll-like receptor signaling pathway - Homo sapiens (human)	KEGG	0.00724
	Activated TLR4 signalling	Reactome	0.00755
	Natural killer cell mediated cytotoxicity - Homo sapiens (human)	KEGG	0.0108
	Cell adhesion molecules (CAMs) - Homo sapiens (human)	KEGG	0.0117
	Regulation of toll-like receptor signaling pathway	Wikipathways	0.0117
	Immunoregulatory interactions between a Lymphoid and a non-Lymphoid cell	Reactome	0.0117
2	NF-kappa B signaling pathway - Homo sapiens (human)	KEGG	6.54E-08
	hiv-1 nef: negative effector of fas and tnf	BioCarta	0.00105
	TLR NFkB	INOH	0.0012
	Corticotropin-releasing hormone	Wikipathways	0.0012
	Toll-like receptor signaling pathway	Wikipathways	0.0012
	Toll-like receptor signaling pathway - Homo sapiens (human)	KEGG	0.0012

	Toll Like Receptor 4 (TLR4) Cascade	Reactome	0.00133
	Toll-Like Receptors Cascades	Reactome	0.00153
	Regulation of toll-like receptor signaling pathway	Wikipathways	0.00153
	Immune System	Reactome	0.00512
	Cytokine Signaling in Immune system	Reactome	0.00734
3	NF-kappa B signaling pathway - Homo sapiens (human)	KEGG	0.00000208
	TNFalpha	NetPath	0.00257
	Cytokine Signaling in Immune system	Reactome	0.00438
4	NF-kappa B signaling pathway - Homo sapiens (human)	KEGG	0.000131
5	NF-kappa B signaling pathway - Homo sapiens (human)	KEGG	6.53E-05
6	NF-kappa B signaling pathway - Homo sapiens (human)	KEGG	6.53E-05

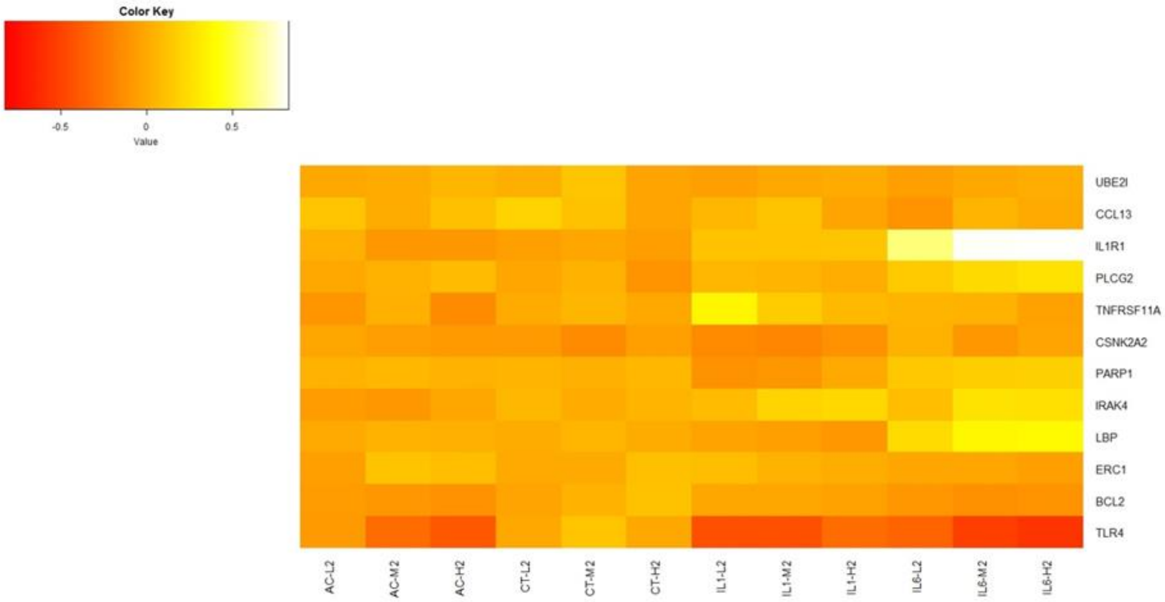

Figure S4-2: Heat map for the 12 start nodes (nodes with only outgoing edges) in the network of Figure 1, displaying the log2 ratios at the earliest time point (2 hours). AC = acetaminophen; CT = carbon tetrachloride; IL1 = interleukin 1; IL6 = interleukin 6; L = low dose; M = middle dose; H = high dose.

Table S4-4: Function of the genes in the network of Figure 1, extracted from GeneCards.

Entrez gene ID	Gene name	subnetwork	function
7329	UBE2I	3	involved in DNA recombination and essential for cell-cycle progression
8837	CFLAR	1	Apoptosis regulator protein
6357	CCL13	5	Plays a role in the accumulation of leukocytes at both sides of allergic and non-allergic inflammation.
3554	IL1R1	1	mediates interleukin-1-dependent activation of NF-kappa-B, MAPK and other pathways.
5336	PLCG2	1	involved in signal transduction, crucial in transmembrane signaling
7412	VCAM1	1	Important in cell-cell recognition, mediates both adhesion and signal transduction
8792	TNFRSF11A	4	Involved in the regulation of interactions between T-cells and dendritic cells.
1459	CSNK2A2	6	Regulates numerous cellular processes, such as cell cycle progression, apoptosis and transcription. Can also negatively regulate apoptosis.
142	PARP1	2	Involved in the base excision repair.
51135	IRAK4	1	plays a critical role in initiating innate immune response against foreign pathogens.
3929	LBP	2	Plays a role in the innate immune response.
23085	ERC1	1	Regulatory subunit of the IKK complex.
4050	LTB	1	May play a specific role in immune response regulation.
596	BCL2	1	key regulator of apoptosis
7099	TLR4	1	mediate the innate immune response to bacterial lipopolysaccharide (LPS), also involved in LPS-independent inflammatory responses
7187	TRAF3	3	plays a central role in the regulation of B-cell survival. Plays a role T-cell dependent immune responses.
7185	TRAF1	5	involved in the blockage of apoptotic pathway, plays a role in the regulation of cell survival and apoptosis.
3383	ICAM1	1	inhibiting interleukin 4 production by naive T cells
10673	TNFSF13B	1	promotes the survival of mature B-cells and the B-cell response, it can directly or indirectly regulate the differential expression of a large number of genes involved in the innate immune response and the regulation of apoptosis.
929	CD14	4	involved in the clearance of apoptotic cells, mediating the innate immune response to bacterial lipopolysaccharide (LPS), acts via the inflammatory response
4616	GADD45B	6	Involved in the regulation of growth and apoptosis.
8740	TNFSF14	2	inducing apoptosis, stimulates the proliferation of T-cells
29760	BLNK	3	regulating biological outcomes of B-cell function and development, plays a critical role in orchestrating the pro-B cell to pre-B cell transition. May play an important role in BCR-induced B-cell apoptosis.
1460	CSNK2B	1	involved in regulation of cell growth
5970	RELA	2	related to many biological processes such as inflammation, immunity, differentiation, cell growth, tumorigenesis and apoptosis.Essential for cytokine gene expression in T-cells

Table S4-5: Unknown interactions in CPDB – putative functional relationships inferred with Biograph, based on known interactions. Gene pairs without functional relationships in BioGraph were omitted from the table. E = expression, PI = protein interaction, GI = genetic interaction, P = phosphorylation.

from	to	putative functional relations	type of interactions	common function of the genes
CFLAR	LTB	CFLAR -> TRAF3 -> TNFSF14 -> LTB	E, PI, PI	inflammatory response
		CFLAR -> TRAF3 -> LTBR -> LTB	E, PI/GI, PI	inflammatory response
		CFLAR -> TRAF1 -> LTBR -> LTB	PI, PI, PI	inflammatory response
PLCG2	ICAM1	PLCG2 -> EGFR -> ICAM1	P/PI, PI	cell-cell communication
TNFRSF11A	CD14	TNFRSF11A -> TAB2 -> LGALS3BP -> CD14	PI, PI, PI	immune response

Table S4-6: Unknown interactions in CPDB – gene pairs with functional interaction in STRING. Gene pairs without functional relationship in STRING were omitted from the table.

from	to	known/predicted/other?	type
PLCG2	ICAM1	predicted	gene neighbourhood

Table S4-7: Unknown interactions in CPDB – relation of gene pairs with one (or more) of the four studied compounds, extracted from CTD. Gene pairs that have no relationship with one of the compounds in CTD were omitted from the table.

from	to	relationship in CTD
CFLAR	LTB	both affected by carbon tetrachloride
PLCG2	ICAM1	both affected by acetaminophen
LBP	TNFSF14	both affected by acetaminophen

Table S4-8: Unknown interactions in CPDB – evidence for a relationship from other databases – summary of Tables S4-6, S4-7, S4-8. Interactions for which no evidence could be found were omitted.

From	To	Biograph	STRING	CTD gene pair - compound
CFLAR	LTB	x		x
PLCG2	ICAM1	x	x	x
TNFRS1A	CD14	x		
LBP	TNFSF14			x
