# Supplementary material for: DTNI: a novel toxicogenomics data analysis tool for identifying the molecular mechanisms underlying the adverse effects of toxic compounds
Source: Arch Toxicol. 2016 Dec 28;91(6):2343–52. doi: 10.1007/s00204-016-1922-5 (PMC5429357; doi:10.1007/s00204-016-1922-5)
Supplement: Supplementary file 6 — Supplementary material 6 (PDF 2325 kb) [file 204_2016_1922_MOESM6_ESM.pdf]

Supplementary Material 5

Article title: DTNI: a novel toxicogenomics data analysis tool for identifying the molecular mechanisms underlying the adverse effects of toxic compounds

Journal name: Archives of Toxicology

Authors names: Diana M. Hendrickx[1], Terezinha Souza[1], Danyel G. J. Jennen[1], Jos C. S. Kleinjans[1]

Affiliation: [1] Department of Toxicogenomics, GROW-School for Oncology and Developmental Biology, Maastricht University, Universiteitssingel 40, 6229 ER Maastricht, The Netherlands. Postal address: P.O. Box 616, 6200 MD Maastricht, The Netherlands. Telephone: +31 43 3881845.

E-mail address of the corresponding author: Diana M. Hendrickx, d.hendrickx@maastrichtuniversity.nl

Example 2: NRF2 pathway – additional information

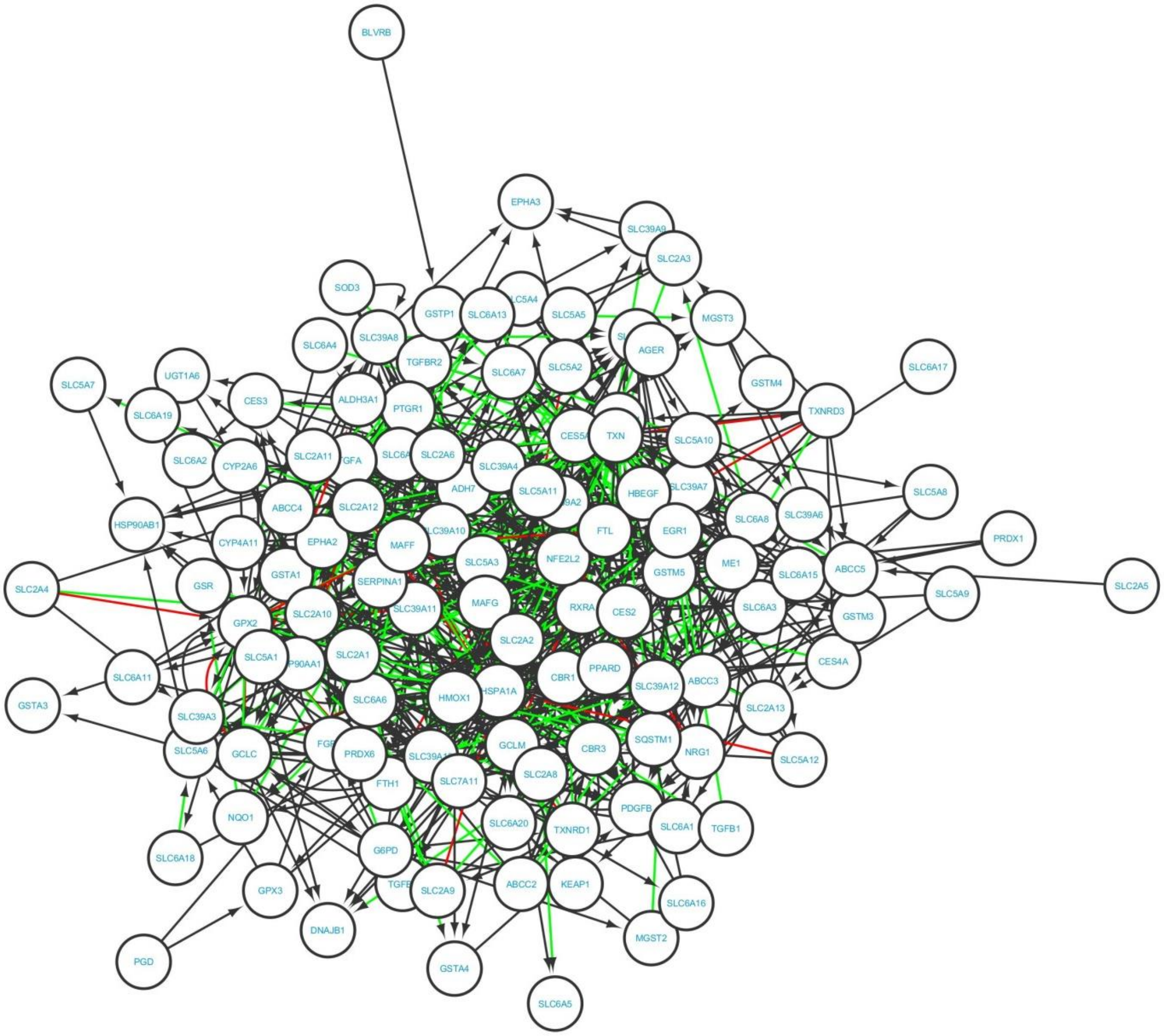

Figure S5-1: NRF2 pathway – interaction network inferred with DTNI for $p \leq 0.05$. Green edges: true positives (TP), red edges: false positives (FP), black edges: novel interactions.

Table S5-1: Details of the edges in the network of Figure S5-1. PI = protein interaction; BI = biochemical interaction.

From (EntrezID)	To (EntrezID)	From (name)	To (name)	Interaction	Intermediate	Intermediate type	Interaction type: gene regulatory and …	Intermediates (EntrezID)	In gene list?	Conclusion
218	4780	ALDH3A1	NFE2L2	none						novel
218	29986	ALDH3A1	SLC39A2	none						novel
218	1969	ALDH3A1	EPHA2	none						novel
645	2950	BLVRB	GSTP1	none						novel
873	10057	CBR1	ABCC5	indirect	PGE2	compound	BI and PI		no	TP
873	3303	CBR1	HSPA1A	indirect	ITGB4BP	gene/protein	PI	3692	no	TP
873	4780	CBR1	NFE2L2	indirect	AK1C1	gene/protein	BI	1645	no	TP
873	2877	CBR1	GPX2	none						novel
874	57181	CBR3	SLC39A10	none						novel
874	54716	CBR3	SLC6A20	none						novel
874	8878	CBR3	SQSTM1	indirect	SGK1	gene/protein	PI and BI	6446	no	TP
874	1244	CBR3	ABCC2	none						novel
874	5467	CBR3	PPARD	none						novel
874	3084	CBR3	NRG1	none						novel
874	2947	CBR3	GSTM3	none						novel
874	201266	CBR3	SLC39A11	none						novel
874	1728	CBR3	NQO1	none						novel
874	6526	CBR3	SLC5A3	none						novel
874	3303	CBR3	HSPA1A	indirect	DCUN1D1	gene/protein	PI	54165	no	TP
874	221074	CBR3	SLC39A12	none						novel
874	5265	CBR3	SERPINA1	none						novel
874	125206	CBR3	SLC5A10	none						novel
874	3320	CBR3	HSP90AA1	indirect	DCUN1D1	gene/protein	PI	54165	no	TP
874	29986	CBR3	SLC39A2	none						novel
874	3337	CBR3	DNAJB1	indirect	CYP2C8	gene/protein	BI and PI	1558	no	TP
874	7295	CBR3	TXN	indirect	DCUN1D1	gene/protein	PI	54165	no	TP
874	2730	CBR3	GCLM	none						novel
874	5155	CBR3	PDGFB	none						novel
1244	8824	ABCC2	CES2	none						novel
1244	5467	ABCC2	PPARD	none						novel
1244	3320	ABCC2	HSP90AA1	indirect	IRF3<br>TOMM70A<br>NFKB1(1-433)	gene/protein	PI	3661<br>9868<br>4790	no<br>no<br>no	TP
1244	221074	ABCC2	SLC39A12	indirect	SLC30A5	gene/protein	PI and BI	64924	no	TP
1244	6533	ABCC2	SLC6A6	none						novel
1244	874	ABCC2	CBR3	none						novel
1244	8878	ABCC2	SQSTM1	indirect	RARA	gene/protein	PI	5914	no	TP
1548	81031	CYP2A6	SLC2A10	none						novel
1548	6513	CYP2A6	SLC2A1	none						novel
1548	57181	CYP2A6	SLC39A10	none						novel
1548	6533	CYP2A6	SLC6A6	none						novel
1548	55630	CYP2A6	SLC39A4	none						novel
1548	2877	CYP2A6	GPX2	none						novel
1579	3303	CYP4A11	HSPA1A	none						novel
1579	57181	CYP4A11	SLC39A10	none						novel
1579	11182	CYP4A11	SLC2A6	none						novel
1579	6513	CYP4A11	SLC2A1	none						novel
1579	6526	CYP4A11	SLC5A3	none						novel
1728	2936	NQO1	GSR	indirect	FAD	compound	PI		no	TP
1728	81031	NQO1	SLC2A10	none						novel
1728	5265	NQO1	SERPINA1	indirect	AP-1_I.complex -><br>CEBPB	complex -><br>gene/protein	---	none -><br>1051	no<br>no	TP
1958	125206	EGR1	SLC5A10	none						novel
1958	3084	EGR1	NRG1	indirect	SREBF2(1-1141)<br>p-10Y-ERBB3-1	gene/protein	PI<br>PI and BI	6721<br>2065	no<br>no	TP
1958	25800	EGR1	SLC39A6	none						novel
1958	22949	EGR1	PTGR1	none						novel
1958	283375	EGR1	SLC39A5	none						novel

From (EntrezID)	To (EntrezID)	From (name)	To (name)	Interaction	Intermediate	Intermediate type	Interaction type: gene regulatory and …	Intermediates (EntrezID)	In gene list?	Conclusion
1969	3303	EPHA2	HSPA1A	indirect	AP2M1	gene/protein	PI	1173	no	TP
					EPS15	gene/protein	PI	2060	no	
					INPPL1	gene/protein	PI	3636	no	
					PIK3R2	gene/protein	PI	5296	no	
					AXL	gene/protein	BI and PI	558	no	
					TP53 Tetramer	complex	---		no	
1969	3320	EPHA2	HSP90AA1	direct			PI			TP
1969	2730	EPHA2	GCLM	none						novel
1969	2877	EPHA2	GPX2	none						novel
1969	2258	EPHA2	FGF13	none						novel
1969	6533	EPHA2	SLC6A6	none						novel
1969	201266	EPHA2	SLC39A11	none						novel
2258	115584	FGF13	SLC5A11	none						novel
2258	8714	FGF13	ABCC3	none						novel
2258	6513	FGF13	SLC2A1	none						novel
2495	4780	FTH1	NFE2L2	direct			---			TP
2495	873	FTH1	CBR1	none						novel
2495	81031	FTH1	SLC2A10	none						novel
2495	5467	FTH1	PPARD	indirect	NCOR1	gene/protein	PI	9611	no	TP
2495	6529	FTH1	SLC6A1	none						novel
2495	3303	FTH1	HSPA1A	indirect	CEP57	gene/protein	PI	9702	no	TP
					ASB16	gene/protein	PI	92591	no	
					CXCR4	gene/protein	PI	7852	no	
2512	6526	FTL	SLC5A3	none						novel
2512	3303	FTL	HSPA1A	indirect	CEP57	gene/protein	PI	9702	no	TP
					PACSIN2		PI	11252	no	
					HSPD1(1-573)		PI	3329	no	
2512	2042	FTL	EPHA3	none						novel
2512	221074	FTL	SLC39A12	none						novel
2512	1839	FTL	HBEGF	none						novel
2512	6534	FTL	SLC6A7	none						novel
2512	125206	FTL	SLC5A10	none						novel
2512	55117	FTL	SLC6A15	none						novel
2512	1958	FTL	EGR1	indirect	GADD45A	gene/protein	PI	1647	no	TP
2512	23764	FTL	MAFF	indirect	NFE2L2	gene/protein	PI	4780	yes	FP
2512	23657	FTL	SLC7A11	none						novel
2512	4780	FTL	NFE2L2	direct			---			TP
2539	4258	G6PD	MGST2	none						novel
2539	7042	G6PD	TGFB2	indirect	ATF2	gene/protein	---	1386	no	TP
2539	873	G6PD	CBR1	none						novel
2539	2258	G6PD	FGF13	none						novel
2539	7296	G6PD	TXNRD1	none						novel
2539	2512	G6PD	FTL	none						novel
2539	6514	G6PD	SLC2A2	none						novel
2539	9817	G6PD	KEAP1	none						novel
2539	8884	G6PD	SLC5A6	none						novel
2539	2938	G6PD	GSTA1	indirect	BAG3	gene/protein	PI	9531	no	TP
					YWHAE		PI	7531	no	
2539	201266	G6PD	SLC39A11	none						novel
2729	3337	GCLC	DNAJB1	none						novel
2729	2877	GCLC	GPX2	indirect	NFE2L2	gene/protein	---	4780	yes	FP
2729	6514	GCLC	SLC2A2	none						novel
2729	3320	GCLC	HSP90AA1	indirect	CYP40	gene/protein	PI	5481	no	TP
2729	23657	GCLC	SLC7A11	none						novel
2729	91252	GCLC	SLC39A13	none						novel
2730	54716	GCLM	SLC6A20	none						novel
2730	2512	GCLM	FTL	indirect	NFE2L2	gene/protein	---	4780	yes	FP
2730	4097	GCLM	MAFG	indirect	JUN	gene/protein	PI	3725	no	TP
					NFE2L2	gene/protein	PI	4780	yes	
2730	2941	GCLM	GSTA4	none						novel
2730	57181	GCLM	SLC39A10	none						novel

From (EntrezID)	To (EntrezID)	From (name)	To (name)	Interaction	Intermediate	Intermediate type	Interaction type: gene regulatory and …	Intermediates (EntrezID)	In gene list?	Conclusion
2730	874	GCLM	CBR3	none						novel
2730	2949	GCLM	GSTM5	none						novel
2730	9588	GCLM	PRDX6	none						novel
2730	10057	GCLM	ABCC5	none						novel
2730	873	GCLM	CBR1	indirect	MCC<br>TRM7	gene/protein<br>gene/protein	PI<br>PI	4163<br>24140	no<br>no	TP
2730	2938	GCLM	GSTA1	none						novel
2730	154091	GCLM	SLC2A12	none						novel
2730	3303	GCLM	HSPA1A	indirect	NFE2L2 -><br>HSPD1(1-573)<br>NFE2L2 -><br>TRIM24-p-2Y-FGFR1<br>fusion	gene/protein<br>gene/protein<br>gene/protein<br>gene/protein	BI and PI<br><br>PI	4780 -><br>3329<br>4780-><br>8805	yes<br>no<br>yes<br>no	FP
2730	55630	GCLM	SLC39A4	none						novel
2730	23657	GCLM	SLC7A11	none						novel
2730	9152	GCLM	SLC6A5	none						novel
2730	3337	GCLM	DNAJB1	none						novel
2877	29985	GPX2	SLC39A3	none						novel
2877	23764	GPX2	MAFF	indirect	NFE2L2	gene/protein	PI	4780	yes	FP
2877	23657	GPX2	SLC7A11	none						novel
2877	2729	GPX2	GCLC	indirect	NFE2L2	gene/protein	---	4780	yes	FP
2877	5265	GPX2	SERPINA1	indirect	TCF1	gene/protein	PI	6927	no	TP
2877	1969	GPX2	EPHA2	none						novel
2877	6533	GPX2	SLC6A6	none						novel
2877	131	GPX2	ADH7	none						novel
2877	201266	GPX2	SLC39A11	none						novel
2877	91252	GPX2	SLC39A13	none						novel
2877	7048	GPX2	TGFBR2	none						novel
2877	3337	GPX2	DNAJB1	none						novel
2877	3320	GPX2	HSP90AA1	none						novel
2877	64116	GPX2	SLC39A8	none						novel
2877	6526	GPX2	SLC5A3	none						novel
2877	873	GPX2	CBR1	none						novel
2877	3326	GPX2	HSP90AB1	none						novel
2877	1728	GPX2	NQO1	indirect	NFE2L2<br>TP63 Tetramer	gene/protein<br>complex	PI<br>BI	4780	yes<br>no	TP
2877	3303	GPX2	HSPA1A	indirect	TP63 tetramer -><br>IKBKB<br>NFE2L2 -><br>TRIM24-p-2Y-FGFR1<br>fusion<br>NFE2L2 -><br>HSPD1 (1-573)	complex<br>gene/protein<br>gene/protein<br>gene/protein<br>gene/protein<br>gene/protein	BI and PI<br><br>BI and PI<br><br>PI	none -><br>3551<br>4780 -><br>8805<br>4780 -><br>3329	no<br>no<br>yes<br>no<br>yes<br>no	TP
2877	9588	GPX2	PRDX6	direct			BI			TP
2877	81031	GPX2	SLC2A10	none						novel
2877	2495	GPX2	FTH1	indirect	NFE2L2	gene/protein	---	4780	yes	FP
2877	348932	GPX2	SLC6A18	none						novel
2877	8884	GPX2	SLC5A6	none						novel
2877	4097	GPX2	MAFG	indirect	NFE2L2	gene/protein	PI	4780	yes	FP
2877	7295	GPX2	TXN	indirect	NFE2L2<br>GPX1	gene/protein	---<br>BI and PI	4780<br>2876	yes<br>no	TP
2877	10257	GPX2	ABCC4	none						novel
2878	8884	GPX3	SLC5A6	none						novel
2878	3303	GPX3	HSPA1A	none						novel
2878	6513	GPX3	SLC2A1	none						novel
2936	3303	GSR	HSPA1A	indirect	ISG15<br>HSPD1(1-573)	gene/protein	PI<br>PI	9636<br>3329	no<br>no	TP
2936	6513	GSR	SLC2A1	none						novel
2938	2730	GSTA1	GCLM	none						novel
2938	55630	GSTA1	SLC39A4	none						novel
2938	3303	GSTA1	HSPA1A	none						novel

From (EntrezID)	To (EntrezID)	From (name)	To (name)	Interaction	Intermediate	Intermediate type	Interaction type: gene regulatory and …	Intermediates (EntrezID)	In gene list?	Conclusion
2938	6513	GSTA1	SLC2A1	none						novel
2938	23491	GSTA1	CES3	none						novel
2938	7048	GSTA1	TGFBR2	indirect	PPP2CB	gene/protein	PI	5516	no	TP
2941	5155	GSTA4	PDGFB	none						novel
2947	221074	GSTM3	SLC39A12	none						novel
2947	4780	GSTM3	NFE2L2	none						novel
2947	1839	GSTM3	HBEGF	none						novel
2947	874	GSTM3	CBR3	none						novel
2947	114134	GSTM3	SLC2A13	none						novel
2947	1958	GSTM3	EGR1	none						novel
2949	10057	GSTM5	ABCC5	none						novel
2949	1958	GSTM5	EGR1	none						novel
2949	8714	GSTM5	ABCC3	none						novel
2949	283375	GSTM5	SLC39A5	none						novel
2949	873	GSTM5	CBR1	none						novel
2949	115584	GSTM5	SLC5A11	none						novel
2949	6534	GSTM5	SLC6A7	indirect	Cl-	compound	BI		no	TP
2949	3303	GSTM5	HSPA1A	none						novel
2949	125206	GSTM5	SLC5A10	none						novel
2949	159963	GSTM5	SLC5A12	none						novel
2949	23657	GSTM5	SLC7A11	none						novel
2950	57181	GSTP1	SLC39A10	indirect	HIST1H4 -><br>CREB1	gene/protein<br>family	PI	8370/<br>8368/<br>8367/<br>8366/<br>8365/<br>8364/<br>8363/<br>8362/<br>8361/<br>8360/<br>8359/<br>8294/<br>554313/<br>-> 1385	no<br>no<br>no<br>no<br>no<br>no<br>no<br>no<br>no<br>no<br>no<br>no<br>no<br>no	TP
2950	3320	GSTP1	HSP90AA1	indirect	MAP3K5<br>ISG15<br>FANCC	gene/protein	PI<br>PI<br>PI	4217<br>9636<br>2176	no<br>no<br>no	TP
2950	7048	GSTP1	TGFBR2	indirect	FANCC<br>JUN	gene/protein	PI<br>PI	2176<br>3725	no<br>no	TP
2950	1958	GSTP1	EGR1	indirect	ATF2<br>JUN	gene/protein	PI<br>PI	1386<br>3725	no<br>no	TP
2950	201266	GSTP1	SLC39A11	none						novel
2950	4259	GSTP1	MGST3	direct			BI			TP
2950	23764	GSTP1	MAFF	none						novel
3084	7295	NRG1	TXN	none						novel
3084	5155	NRG1	PDGFB	none						novel
3084	6533	NRG1	SLC6A6	none						novel
3084	4097	NRG1	MAFG	none						novel
3084	4780	NRG1	NFE2L2	indirect	HMOX1	gene/protein	PI	3162	yes	FP
3084	29986	NRG1	SLC39A2	none						novel
3084	91252	NRG1	SLC39A13	none						novel
3084	874	NRG1	CBR3	none						novel
3162	873	HMOX1	CBR1	indirect	HLA-C	gene/protein	PI	3107	no	TP
3162	6533	HMOX1	SLC6A6	none						novel
3162	2936	HMOX1	GSR	indirect	FAD	compound	BI and PI		no	TP
3162	29986	HMOX1	SLC39A2	none						novel
3162	57181	HMOX1	SLC39A10	none						novel
3162	3303	HMOX1	HSPA1A	indirect	POT1	gene/protein	PI	25913	no	TP
3162	201266	HMOX1	SLC39A11	none						novel

From (EntrezID)	To (EntrezID)	From (name)	To (name)	Interaction	Intermediate	Intermediate type	Interaction type: gene regulatory and …	Intermediates (EntrezID)	In gene list?	Conclusion
3162	1958	HMOX1	EGR1	indirect	ATF2	gene/protein	---	1386	no	TP
					NOS1		PI	4842	no	
					CREB3		PI	10488	no	
3162	2878	HMOX1	GPX3	none						novel
3162	2495	HMOX1	FTH1	indirect	NFE2L2	gene/protein	---	4780	yes	TP
					Fe3+	compound	BI		no	
3162	3320	HMOX1	HSP90AA1	indirect	NOS1	gene/protein	PI	4842	no	TP
3162	2941	HMOX1	GSTA4	none						novel
3162	23516	HMOX1	SLC39A14	none						novel
3162	221223	HMOX1	CES5A	none						novel
3162	81031	HMOX1	SLC2A10	none						novel
3162	3084	HMOX1	NRG1	direct			PI			TP
3303	6256	HSPA1A	RXRA	indirect	MED1	gene/protein	PI	5469	no	TP
					TBP		PI	6908	no	
					HMGA1		PI	3159	no	
					NFKBIB		PI	4793	no	
					TIF1A		PI	8805	no	
3303	873	HSPA1A	CBR1	indirect	ITGB4BP	gene/protein	PI	3692	no	TP
3303	91252	HSPA1A	SLC39A13	indirect	REL	gene/protein	PI	5966	no	TP
3303	8824	HSPA1A	CES2	none						novel
3303	57181	HSPA1A	SLC39A10	none						novel
3303	8884	HSPA1A	SLC5A6	none						novel
3303	2512	HSPA1A	FTL	indirect	CEP57	gene/protein	PI	9702	no	TP
					PACSIN2			11252	no	
3303	221074	HSPA1A	SLC39A12	none						novel
3303	8714	HSPA1A	ABCC3	none						novel
3303	11182	HSPA1A	SLC2A6	indirect	SLC5A1	gene/protein	PI and BI	6523	yes	FP
3303	4097	HSPA1A	MAFG	none						novel
3303	7042	HSPA1A	TGFB2	none						novel
3320	3303	HSP90AA1	HSPA1A	direct			PI/BI			TP
3320	4780	HSP90AA1	NFE2L2	indirect	MAFG	gene/protein	PI	4097	yes	TP
					PMF1	gene/protein	PI	11243	no	
					GNAI2	gene/protein	PI	2771	no	
					HSP40	gene/protein	BI	171221	no	
					AHR	gene/protein	PI	196	no	
					enc1_human	gene/protein	PI	8507	no	
					CK2.complex	complex	PI	---	no	
					PRKC.family	group	BI	---	no	
					PKC	group	BI	---	no	
3320	57181	HSP90AA1	SLC39A10	none						novel
3320	2877	HSP90AA1	GPX2	none						novel
3326	6536	HSP90AB1	SLC6A9	none						novel
4097	6531	MAFG	SLC6A3	none						novel
4097	54716	MAFG	SLC6A20	none						novel
4097	2495	MAFG	FTH1	indirect	NFE2L2	gene/protein	PI	4780	yes	FP
4097	221074	MAFG	SLC39A12	none						novel
4097	5467	MAFG	PPARD	none						novel
4097	7295	MAFG	TXN	indirect	NFE2L2	gene/protein	PI	4780	yes	TP
					NFE2		PI	4778	no	
					VIM		PI	7431	no	
4097	5155	MAFG	PDGFB	none						novel
4097	7296	MAFG	TXNRD1	indirect	NFE2L2	gene/protein	PI	4780	yes	FP
4097	6529	MAFG	SLC6A1	none						novel
4097	7922	MAFG	SLC39A7	none						novel
4097	3320	MAFG	HSP90AA1	direct			PI			TP
4097	4780	MAFG	NFE2L2	direct			PI			TP
4097	5265	MAFG	SERPINA1	none						novel
4097	283375	MAFG	SLC39A5	none						novel
4097	23657	MAFG	SLC7A11	none						novel
4097	3303	MAFG	HSPA1A	none						novel

From (EntrezID)	To (EntrezID)	From (name)	To (name)	Interaction	Intermediate	Intermediate type	Interaction type: gene regulatory and . .	Intermediates (EntrezID)	In gene list?	Conclusion
4097	7048	MAFG	TGFBR2	indirect	JUN<br>PPP1CA	gene/protein	PI<br>PI	3725<br>5499	no<br>no	TP
4097	6532	MAFG	SLC6A4	none						novel
4097	4199	MAFG	ME1	none						novel
4097	3084	MAFG	NRG1	none						novel
4199	125206	ME1	SLC5A10	none						novel
4199	874	ME1	CBR3	indirect	NADH	compound	BI		no	TP
4199	283375	ME1	SLC39A5	none						novel
4199	7048	ME1	TGFBR2	indirect	NFYA	gene/protein	PI	4800	no	TP
4199	3303	ME1	HSPA1A	none						novel
4199	4097	ME1	MAFG	none						novel
4199	873	ME1	CBR1	indirect	NADH<br>NAD+	compound	BI<br>BI		no<br>no	TP
4199	6529	ME1	SLC6A1	none						novel
4199	221074	ME1	SLC39A12	none						novel
4199	4780	ME1	NFE2L2	none						novel
4258	2949	MGST2	GSTM5	direct			BI			TP
4258	54716	MGST2	SLC6A20	none						novel
4259	25800	MGST3	SLC39A6	none						novel
4259	10057	MGST3	ABCC5	none						novel
4780	29986	NFE2L2	SLC39A2	none						novel
4780	4097	NFE2L2	MAFG	direct			PI			TP
4780	64116	NFE2L2	SLC39A8	none						novel
4780	131	NFE2L2	ADH7	none						novel
4780	10057	NFE2L2	ABCC5	none						novel
4780	5155	NFE2L2	PDGFB	none						novel
4780	1969	NFE2L2	EPHA2	indirect	KPNA3	gene/protein	PI	3839	no	TP
4780	7039	NFE2L2	TGFA	none						novel
4780	6514	NFE2L2	SLC2A2	none						novel
4780	8824	NFE2L2	CES2	none						novel
4780	2730	NFE2L2	GCLM	direct			---			TP
4780	6526	NFE2L2	SLC5A3	none						novel
4780	7295	NFE2L2	TXN	direct			---			TP
4780	874	NFE2L2	CBR3	indirect	AKR1C1	gene/protein	BI	1645	no	TP
4780	6534	NFE2L2	SLC6A7	none						novel
4780	1839	NFE2L2	HBEGF	none						novel
4780	5265	NFE2L2	SERPINA1	indirect	CK2.complex -><br>NCOA2(869-1464)	complex -><br>gene/protein	BI	10499	no	TP
4780	9588	NFE2L2	PRDX6	indirect	PRDX1<br>GPX2<br>RARA	gene/protein	BI/PI<br>BI<br>PI	5052<br>2877<br>5914	yes<br>yes<br>no	TP
4780	221074	NFE2L2	SLC39A12	none						novel
5052	2949	PRDX1	GSTM5	none						novel
5052	55117	PRDX1	SLC6A15	none						novel
5052	10057	PRDX1	ABCC5	none						novel
5155	4097	PDGFB	MAFG	none						novel
5155	3303	PDGFB	HSPA1A	none						novel
5155	8824	PDGFB	CES2	none						novel
5155	874	PDGFB	CBR3	none						novel
5155	3084	PDGFB	NRG1	none						novel
5155	5265	PDGFB	SERPINA1	indirect	LRP1<br>A2M	gene/protein	PI<br>PI and BI	4035<br>2	no<br>no	TP
5226	2258	PGD	FGF13	none						novel
5226	2878	PGD	GPX3	none						novel
5265	8824	SERPINA1	CES2	none						novel
5265	1548	SERPINA1	CYP2A6	none						novel
5265	6526	SERPINA1	SLC5A3	none						novel
5265	4097	SERPINA1	MAFG	none						novel
5265	3326	SERPINA1	HSP90AB1	none						novel
5265	81031	SERPINA1	SLC2A10	none						novel

From (EntrezID)	To (EntrezID)	From (name)	To (name)	Interaction	Intermediate	Intermediate type	Interaction type: gene regulatory and . . .	Intermediates (EntrezID)	In gene list?	Conclusion
5265	3320	SERPINA1	HSP90AA1	indirect	MIS12<br>LRP1<br>p-S795-RB1<br>CEBPB	gene/protein	PI<br>PI<br>BI<br>---	79003<br>4035<br>5925<br>1051	no<br>no<br>no<br>no	TP
5265	10257	SERPINA1	ABCC4	none						novel
5265	2729	SERPINA1	GCLC	none						novel
5265	8884	SERPINA1	SLC5A6	none						novel
5265	7295	SERPINA1	TXN	indirect	p-S795-RB1	gene/protein	BI	5925	no	TP
5265	6513	SERPINA1	SLC2A1	indirect	CANX	gene/protein	PI	821	no	TP
5265	64116	SERPINA1	SLC39A8	indirect	Zn2+	compound	PI and BI		no	TP
5265	874	SERPINA1	CBR3	none						novel
5265	201266	SERPINA1	SLC39A11	indirect	Zn2+	compound	PI and BI		no	TP
5265	1728	SERPINA1	NQO1	indirect	CEBPB -><br>AP-1_I.complex	gene/protein<br>-> complex	---	1051<br>none	no<br>no	TP
5265	9588	SERPINA1	PRDX6	indirect	HNF4G	gene/protein	PI	3174	no	TP
5265	1839	SERPINA1	HBEGF	indirect	MMP7	gene/protein	PI	4316	no	TP
5265	22949	SERPINA1	PTGR1	none						novel
5265	1969	SERPINA1	EPHA2	indirect	FOXA1<br>TP53 Tetramer	gene/protein<br>complex	---	3169<br>----	no<br>no	TP
5265	2877	SERPINA1	GPX2	indirect	TCF1	gene/protein	PI	6927	no	TP
5265	5155	SERPINA1	PDGFB	indirect	LRP1<br>A2M	gene/protein	PI<br>BI and PI	4035<br>2	no<br>no	TP
5265	131	SERPINA1	ADH7	indirect	Zn2+	compound	PI		no	TP
5265	57181	SERPINA1	SLC39A10	indirect	Zn2+	compound	PI and BI		no	TP
5265	218	SERPINA1	ALDH3A1	indirect	ALDH2	gene/protein	PI and BI	217	no	TP
5265	5467	SERPINA1	PPARD	indirect	NCOA2(869-1464)	gene/protein	PI	10499	no	TP
5265	6534	SERPINA1	SLC6A7	none						novel
5265	7048	SERPINA1	TGFBR2	none						novel
5265	4780	SERPINA1	NFE2L2	indirect	NCOA2(869-1464) -><br>CK2.complex	gene/protein<br>complex	BI	10499<br>none	no<br>no	TP
5265	6256	SERPINA1	RXRA	indirect	NCOA2(869-1464)<br>Zn2+	gene/protein<br>compound	BI/PI<br>PI	10499<br>none	no<br>no	TP
5265	23657	SERPINA1	SLC7A11	none						novel
5265	3303	SERPINA1	HSPA1A	indirect	PLG(20-810)	gene/protein	BI and PI	6927	no	TP
5265	873	SERPINA1	CBR1	none						novel
5467	115584	PPARD	SLC5A11	none						novel
5467	1839	PPARD	HBEGF	indirect	BCL6	gene/protein	PI	604	no	TP
6256	7042	RXRA	TGFB2	none						novel
6256	55334	RXRA	SLC39A9	indirect	Zn2+	compound	PI and BI		no	TP
6256	6533	RXRA	SLC6A6	none						novel
6256	873	RXRA	CBR1	indirect	ESR1<br>CYP2C8	gene/protein	PI<br>PI and BI	2099<br>1558	no<br>no	TP
6256	2512	RXRA	FTL	indirect	GADD45A	gene/protein	PI	1647	no	TP
6256	6526	RXRA	SLC5A3	none						novel
6256	57181	RXRA	SLC39A10	indirect	Zn2+	compound	PI and BI		no	TP
6256	2949	RXRA	GSTM5	none						novel
6256	3303	RXRA	HSPA1A	indirect	NFKBIB<br>TBP<br>MED1<br>TRIM24-p-2Y-FGFR1 fusion<br>HMGA1	gene/protein	PI<br>PI<br>PI<br>PI<br>PI	4793<br>6908<br>5469<br>8805<br>3159	no<br>no<br>no<br>no<br>no	TP
6513	4780	SLC2A1	NFE2L2	none						novel
6513	2258	SLC2A1	FGF13	none						novel
6513	11182	SLC2A1	SLC2A6	direct			BI			TP
6513	23657	SLC2A1	SLC7A11	none						novel
6513	56606	SLC2A1	SLC2A9	direct			BI			TP
6513	221223	SLC2A1	CES5A	none						novel
6513	3320	SLC2A1	HSP90AA1	indirect	Myc/Max heterodimer<br>TYK2<br>SLC2A4	complex<br>gene/protein<br>gene/protein	---<br>PI<br>BI and PI	none<br>7297<br>6517	no<br>no<br>yes	TP

From (EntrezID)	To (EntrezID)	From (name)	To (name)	Interaction	Intermediate	Intermediate type	Interaction type: gene regulatory and …	Intermediates (EntrezID)	In gene list?	Conclusion
6513	9817	SLC2A1	KEAP1	none						novel
6513	2877	SLC2A1	GPX2	none						novel
6513	1958	SLC2A1	EGR1	indirect	CALR	gene/protein	PI	3830	no	TP
6513	57181	SLC2A1	SLC39A10	none						novel
6514	1958	SLC2A2	EGR1	none						novel
6514	4097	SLC2A2	MAFG	none						novel
6514	6256	SLC2A2	RXRA	none						novel
6514	10257	SLC2A2	ABCC4	none						novel
6514	6526	SLC2A2	SLC5A3	direct			BI			TP
6514	7048	SLC2A2	TGFBR2	none						novel
6514	23764	SLC2A2	MAFF	none						novel
6514	5155	SLC2A2	PDGFB	none						novel
6514	23516	SLC2A2	SLC39A14	none						novel
6514	2730	SLC2A2	GCLM	none						novel
6514	1969	SLC2A2	EPHA2	none						novel
6514	4780	SLC2A2	NFE2L2	none						novel
6514	91252	SLC2A2	SLC39A13	none						novel
6514	873	SLC2A2	CBR1	none						novel
6514	1839	SLC2A2	HBEGF	none						novel
6514	3303	SLC2A2	HSPA1A	indirect	SLC5A1<br>p-T611-FOXM1	gene/protein	BI and PI	6523<br>2305	yes<br>no	TP
6515	22949	SLC2A3	PTGR1	none						novel
6515	2042	SLC2A3	EPHA3	none						novel
6515	7048	SLC2A3	TGFBR2	none						novel
6515	4780	SLC2A3	NFE2L2	indirect	CREB3	gene/protein	PI	10488	no	TP
6517	10257	SLC2A4	ABCC4	none						novel
6517	81031	SLC2A4	SLC2A10	direct			BI			TP
6517	29985	SLC2A4	SLC39A3	none						novel
6517	2877	SLC2A4	GPX2	indirect	PPARG -><br>NFE2L2	gene/protein<br>gene/protein	PI	5468 -><br>4780	no<br>yes	FP
6518	10057	SLC2A5	ABCC5	none						novel
6523	6513	SLC5A1	SLC2A1	direct			BI			TP
6523	7042	SLC5A1	TGFB2	none						novel
6523	6536	SLC5A1	SLC6A9	indirect	Na+	compound	BI		no	TP
6523	23491	SLC5A1	CES3	none						novel
6523	3303	SLC5A1	HSPA1A	direct			PI			TP
6523	218	SLC5A1	ALDH3A1	none						novel
6523	5467	SLC5A1	PPARD	none						novel
6524	1958	SLC5A2	EGR1	none						novel
6524	55334	SLC5A2	SLC39A9	none						novel
6524	6526	SLC5A2	SLC5A3	direct			BI			TP
6524	3303	SLC5A2	HSPA1A	indirect	SLC5A1	gene/protein	BI and PI	6523	yes	FP
6524	5265	SLC5A2	SERPINA1	none						novel
6526	5155	SLC5A3	PDGFB	none						novel
6526	1969	SLC5A3	EPHA2	none						novel
6526	4780	SLC5A3	NFE2L2	none						novel
6526	5265	SLC5A3	SERPINA1	none						novel
6526	2877	SLC5A3	GPX2	none						novel
6526	1839	SLC5A3	HBEGF	none						novel
6526	8824	SLC5A3	CES2	none						novel
6526	23764	SLC5A3	MAFF	none						novel
6526	221074	SLC5A3	SLC39A12	none						novel
6526	873	SLC5A3	CBR1	none						novel
6526	64116	SLC5A3	SLC39A8	none						novel
6526	3303	SLC5A3	HSPA1A	indirect	SLC5A1	gene/protein	BI and PI	6523	yes	FP
6526	7295	SLC5A3	TXN	none						novel
6527	5265	SLC5A4	SERPINA1	none						novel
6527	1958	SLC5A4	EGR1	none						novel
6527	22949	SLC5A4	PTGR1	none						novel
6527	7295	SLC5A4	TXN	none						novel

From (EntrezID)	To (EntrezID)	From (name)	To (name)	Interaction	Intermediate	Intermediate type	Interaction type: gene regulatory and . .	Intermediates (EntrezID)	In gene list?	Conclusion
6527	57181	SLC5A4	SLC39A10	none						novel
6528	23516	SLC5A5	SLC39A14	none						novel
6528	57181	SLC5A5	SLC39A10	none						novel
6528	201266	SLC5A5	SLC39A11	none						novel
6528	1958	SLC5A5	EGR1	none						novel
6529	3303	SLC6A1	HSPA1A	none						novel
6529	3084	SLC6A1	NRG1	none						novel
6529	7042	SLC6A1	TGFB2	none						novel
6529	10057	SLC6A1	ABCC5	none						novel
6529	4199	SLC6A1	ME1	none						novel
6530	4097	SLC6A2	MAFG	none						novel
6530	6526	SLC6A2	SLC5A3	indirect	Na+	compound	BI		no	TP
6532	7295	SLC6A4	TXN	indirect	CAMK2A	gene/protein	BI	815	no	TP
6532	2877	SLC6A4	GPX2	none						novel
6533	23491	SLC6A6	CES3	none						novel
6533	6526	SLC6A6	SLC5A3	indirect	Na+	compound	BI		no	TP
6533	1728	SLC6A6	NQO1	none						novel
6533	81031	SLC6A6	SLC2A10	indirect	Na+	compound	BI		no	TP
6533	6513	SLC6A6	SLC2A1	indirect	Na+	compound	BI		no	TP
6533	5467	SLC6A6	PPARD	none						novel
6533	2936	SLC6A6	GSR	none						novel
6533	2877	SLC6A6	GPX2	none						novel
6533	8824	SLC6A6	CES2	none						novel
6533	3303	SLC6A6	HSPA1A	none						novel
6533	2941	SLC6A6	GSTA4	indirect	Cl-	compound	BI		no	TP
6533	2729	SLC6A6	GCLC	indirect	EGR1	gene/protein	---	1958	yes	FP
6533	3326	SLC6A6	HSP90AB1	none						novel
6533	221074	SLC6A6	SLC39A12	none						novel
6533	4780	SLC6A6	NFE2L2	none						novel
6534	81031	SLC6A7	SLC2A10	indirect	Na+	compound	BI		no	TP
6534	1969	SLC6A7	EPHA2	none						novel
6534	57181	SLC6A7	SLC39A10	none						novel
6534	1958	SLC6A7	EGR1	none						novel
6535	221223	SLC6A8	CES5A	none						novel
6535	3303	SLC6A8	HSPA1A	none						novel
6535	6515	SLC6A8	SLC2A3	indirect	Na+	compound	BI		no	TP
6535	1839	SLC6A8	HBEGF	none						novel
6535	5155	SLC6A8	PDGFB	none						novel
6536	55630	SLC6A9	SLC39A4	none						novel
6536	6513	SLC6A9	SLC2A1	indirect	Na+	compound	BI		no	TP
6536	2042	SLC6A9	EPHA3	none						novel
6536	4780	SLC6A9	NFE2L2	none						novel
6536	3303	SLC6A9	HSPA1A	none						novel
6536	2730	SLC6A9	GCLM	none						novel
6536	1839	SLC6A9	HBEGF	none						novel
6536	2949	SLC6A9	GSTM5	indirect	Cl-	compound	BI		no	TP
6538	91252	SLC6A11	SLC39A13	none						novel
6538	1969	SLC6A11	EPHA2	none						novel
6538	2877	SLC6A11	GPX2	none						novel
6540	283375	SLC6A13	SLC39A5	none						novel
6540	221223	SLC6A13	CES5A	none						novel
6540	2949	SLC6A13	GSTM5	indirect	Cl-	compound	BI		no	TP
6540	4780	SLC6A13	NFE2L2	none						novel
6540	55334	SLC6A13	SLC39A9	none						novel
6540	81031	SLC6A13	SLC2A10	indirect	Na+	compound	BI		no	TP
6649	4097	SOD3	MAFG	none						novel
6649	64116	SOD3	SLC39A8	none						novel
6649	7048	SOD3	TGFBR2	indirect	SP1	gene/protein	PI	6667	no	TP
6649	6526	SOD3	SLC5A3	none						novel
7039	2258	TGFA	FGF13	none						novel

From (EntrezID)	To (EntrezID)	From (name)	To (name)	Interaction	Intermediate	Intermediate type	Interaction type: gene regulatory and …	Intermediates (EntrezID)	In gene list?	Conclusion
7039	4780	TGFA	NFE2L2	none						novel
7039	29986	TGFA	SLC39A2	none						novel
7039	7048	TGFA	TGFBR2	indirect	PKN3	gene/protein	BI and PI	29941	no	TP
					p-Y,Y877-ERBB2		BI/PI	2064	no	
7039	8824	TGFA	CES2	none						novel
7039	7295	TGFA	TXN	indirect	MAPK11	gene/protein	BI	5600	no	TP
7039	2729	TGFA	GCLC	none						novel
7039	23764	TGFA	MAFF	none						novel
7039	131	TGFA	ADH7	none						novel
7040	3303	TGFB1	HSPA1A	indirect	EPS15	gene/protein	PI	2060	no	TP
					EZH2		PI	2146	no	
7040	1958	TGFB1	EGR1	direct			PI			TP
7042	6514	TGFB2	SLC2A2	none						novel
7048	4199	TGFBR2	ME1	indirect	NFYA	gene/protein	PI	4800	no	TP
7048	23516	TGFBR2	SLC39A14	none						novel
7048	2936	TGFBR2	GSR	indirect	PJA1	gene/protein	BI and PI	64219	no	TP
7295	873	TXN	CBR1	indirect	class I MHC B42	gene/protein	PI	3106	no	TP
					HLA-B	gene/protein		3106	no	
					EIF1B	gene/protein		10289	no	
					Protein 4.1	gene/protein		2035	no	
7295	1969	TXN	EPHA2	indirect	FER	gene/protein	BI	2241	no	TP
					p-Y-JAK1		BI	3716	no	
7295	177	TXN	AGER	indirect	HMGB1	gene/protein	PI and BI	3146	no	TP
					RHOA		BI and PI	387	no	
7295	5265	TXN	SERPINA1	indirect	p-S795-RB1	gene/protein	BI	5925	no	TP
7295	6514	TXN	SLC2A2	none						novel
7295	4780	TXN	NFE2L2	direct			---			TP
7295	8824	TXN	CES2	none						novel
7295	283848	TXN	CES4A	none						novel
7295	4097	TXN	MAFG	indirect	VIM	gene/protein	PI	7431	no	TP
					NFE2		PI	4778	no	
					NFE2L2		PI	4780	yes	
7295	3303	TXN	HSPA1A	indirect	HDJ2	gene/protein	PI	3301	no	TP
					MAP3K5	gene/protein	BI and PI	4217	no	
					IL7R	gene/protein	PI	3575	no	
					HSP70.family	group	BI	---	no	
7295	160728	TXN	SLC5A8	none						novel
7295	3084	TXN	NRG1	none						novel
7295	114112	TXN	TXNRD3	indirect	TXNRD1	gene/protein	PI and BI	7296	yes	FP
7295	25800	TXN	SLC39A6	none						novel
7295	6531	TXN	SLC6A3	indirect	SP1	gene/protein	BI	6667	no	TP
7295	1958	TXN	EGR1	indirect	STAT3	gene/protein	BI and PI	6774	no	TP
					GABPA		BI and PI	2551	no	
					MYD88		PI	4615	no	
7295	6534	TXN	SLC6A7	none						novel
7295	5467	TXN	PPARD	none						novel
7295	8878	TXN	SQSTM1	indirect	MYD88	gene/protein	PI	4615	no	TP
					NFE2L2		PI	4780	yes	
					MLH1		PI	4292	no	
					CAPN1		BI	823	no	
7295	1839	TXN	HBEGF	none						novel
7295	874	TXN	CBR3	indirect	DCUN1D1	gene/protein	PI	54165	no	TP
7295	55630	TXN	SLC39A4	indirect	PTPN1	gene/protein	BI and PI	5770	no	TP
7296	6513	TXNRD1	SLC2A1	indirect	TXNIP	gene/protein	PI	10628	no	TP
7296	2730	TXNRD1	GCLM	indirect	NFE2L2	gene/protein	---	4780	yes	FP
7296	3303	TXNRD1	HSPA1A	indirect	ISG15	gene/protein	PI	9636	no	TP
					TUBA4A	gene/protein	PI	7277	no	
7296	23657	TXNRD1	SLC7A11	none						novel
7296	1839	TXNRD1	HBEGF	none						novel
7922	221074	SLC39A7	SLC39A12	direct			BI			TP
7922	23516	SLC39A7	SLC39A14	direct			BI			TP

From (EntrezID)	To (EntrezID)	From (name)	To (name)	Interaction	Intermediate	Intermediate type	Interaction type: gene regulatory and …	Intermediates (EntrezID)	In gene list?	Conclusion
7922	873	SLC39A7	CBR1	none						novel
7922	23764	SLC39A7	MAFF	indirect	MAX	gene/protein	---	4149	no	TP
8714	5155	ABCC3	PDGFB	none						novel
8714	6526	ABCC3	SLC5A3	none						novel
8714	873	ABCC3	CBR1	none						novel
8714	3303	ABCC3	HSPA1A	none						novel
8714	56606	ABCC3	SLC2A9	none						novel
8714	2949	ABCC3	GSTM5	none						novel
8714	125206	ABCC3	SLC5A10	none						novel
8714	2258	ABCC3	FGF13	none						novel
8824	4780	CES2	NFE2L2	none						novel
8824	2730	CES2	GCLM	none						novel
8824	23764	CES2	MAFF	indirect	MAX	gene/protein	---	4149	no	TP
8824	218	CES2	ALDH3A1	none						novel
8824	5265	CES2	SERPINA1	none						novel
8824	201266	CES2	SLC39A11	none						novel
8824	7295	CES2	TXN	none						novel
8824	1969	CES2	EPHA2	none						novel
8824	7039	CES2	TGFA	none						novel
8824	5155	CES2	PDGFB	none						novel
8878	3303	SQSTM1	HSPA1A	indirect	MAP3K3<br>MAPT<br>MAP1LC3A<br>TG (C)<br>TAB2<br>RAD23A<br>NOD2	gene/protein	PI<br>BI and PI<br>PI<br>PI<br>BI and PI<br>PI<br>PI	4215<br>4137<br>84557<br>7052<br>23118<br>5886<br>64127	no<br>no<br>no<br>no<br>no<br>no<br>no	TP
8878	221223	SQSTM1	CES5A	none						novel
8878	4780	SQSTM1	NFE2L2	direct			PI			TP
8878	2730	SQSTM1	GCLM	indirect	NFE2L2<br>CALM1	gene/protein<br>gene/protein family	PI<br>PI	4780<br>801<br>805<br>808	yes<br>no	TP
8878	3084	SQSTM1	NRG1	indirect	CAMK2A	gene/protein	PI and BI	815	no	TP
8878	4199	SQSTM1	ME1	none						novel
8878	81031	SQSTM1	SLC2A10	none						novel
8878	874	SQSTM1	CBR3	indirect	SGK1	gene/protein	BI and PI	6446	no	TP
8884	2940	SLC5A6	GSTA3	none						novel
8884	6526	SLC5A6	SLC5A3	indirect	Na+	compound	BI		no	TP
8884	7042	SLC5A6	TGFB2	none						novel
8884	2877	SLC5A6	GPX2	none						novel
8884	3326	SLC5A6	HSP90AB1	none						novel
9588	23764	PRDX6	MAFF	indirect	HDAC5	gene/protein	PI	10014	no	TP
9588	221074	PRDX6	SLC39A12	none						novel
9817	874	KEAP1	CBR3	none						novel
9817	4780	KEAP1	NFE2L2	direct			PI/BI			TP
9817	3303	KEAP1	HSPA1A	indirect	IKBKB<br>MAP1LC3A<br>PTMA<br>PGAM5<br>IRF1<br>RBX1	gene/protein	PI<br>PI<br>PI<br>PI<br>PI<br>PI	3551<br>84557<br>5757<br>192111<br>3659<br>9978	no<br>no<br>no<br>no<br>no<br>no	TP
9817	2730	KEAP1	GCLM	indirect	NFE2L2	gene/protein	BI/PI	4780	yes	FP
10057	1839	ABCC5	HBEGF							
10057	7295	ABCC5	TXN	indirect	GSH	compound	PI and BI		no	TP
10057	221074	ABCC5	SLC39A12	none						novel
10257	1839	ABCC4	HBEGF	none						novel
11182	4780	SLC2A6	NFE2L2	none						novel
11182	3303	SLC2A6	HSPA1A	indirect	SLC5A1	gene/protein	BI and PI	6523	yes	FP
11182	4259	SLC2A6	MGST3	none						novel
11182	2938	SLC2A6	GSTA1	none						novel

From (EntrezID)	To (EntrezID)	From (name)	To (name)	Interaction	Intermediate	Intermediate type	Interaction type: gene regulatory and ...	Intermediates (EntrezID)	In gene list?	Conclusion
11182	1969	SLC2A6	EPHA2	none						novel
11182	873	SLC2A6	CBR1	none						novel
11182	283375	SLC2A6	SLC39A5	none						novel
11182	7295	SLC2A6	TXN	none						novel
11182	6526	SLC2A6	SLC5A3	direct			BI			TP
11182	2877	SLC2A6	GPX2	none						novel
11182	2949	SLC2A6	GSTM5	none						novel
11182	8824	SLC2A6	CES2	indirect	MAX	gene/protein	---	4149	no	TP
11182	2495	SLC2A6	FTH1	none						novel
11182	3326	SLC2A6	HSP90AB1	none						novel
22949	221074	PTGR1	SLC39A12	none						novel
22949	221223	PTGR1	CES5A	none						novel
22949	23491	PTGR1	CES3	none						novel
22949	6649	PTGR1	SOD3	none						novel
22949	29986	PTGR1	SLC39A2	none						novel
22949	218	PTGR1	ALDH3A1	none						novel
22949	6533	PTGR1	SLC6A6	none						novel
22949	201266	PTGR1	SLC39A11	none						novel
22949	57181	PTGR1	SLC39A10	none						novel
22949	2877	PTGR1	GPX2	none						novel
22949	23764	PTGR1	MAFF	none						novel
22949	54578	PTGR1	UGT1A6	none						novel
22949	2936	PTGR1	GSR	indirect	NADPH	compound	BI		no	TP
22949	6513	PTGR1	SLC2A1	none						novel
22949	2938	PTGR1	GSTA1	none						novel
22949	3303	PTGR1	HSPA1A	none						novel
23491	6530	CES3	SLC6A2	none						novel
23491	221223	CES3	CES5A	direct			BI		no	TP
23491	11182	CES3	SLC2A6	none						novel
23491	57181	CES3	SLC39A10	none						novel
23516	4780	SLC39A14	NFE2L2	none						novel
23516	8714	SLC39A14	ABCC3	none						novel
23516	6514	SLC39A14	SLC2A2	none						novel
23516	81031	SLC39A14	SLC2A10	none						novel
23516	4097	SLC39A14	MAFG	none						novel
23516	8824	SLC39A14	CES2	none						novel
23516	29986	SLC39A14	SLC39A2	direct			BI		no	TP
23516	1958	SLC39A14	EGR1	none						novel
23657	2938	SLC7A11	GSTA1	none						novel
23657	6533	SLC7A11	SLC6A6	none						novel
23657	3303	SLC7A11	HSPA1A	indirect	EZH2<br>TERT	gene/protein<br>gene/protein	PI<br>PI	2146<br>7015	no<br>no	TP
23657	3337	SLC7A11	DNAJB1	none						novel
23657	8878	SLC7A11	SQSTM1	none						novel
23657	874	SLC7A11	CBR3	none						novel
23657	29985	SLC7A11	SLC39A3	none						novel
23657	2949	SLC7A11	GSTM5	none						novel
23657	81031	SLC7A11	SLC2A10	none						novel
23657	23764	SLC7A11	MAFF	none						novel
23657	8714	SLC7A11	ABCC3	none						novel
23764	7039	MAFF	TGFA	none						novel
23764	3303	MAFF	HSPA1A	indirect	NR3C1-1	gene/protein	PI	2908	no	TP
23764	55630	MAFF	SLC39A4	none						novel
23764	54578	MAFF	UGT1A6	none						novel
23764	91252	MAFF	SLC39A13	none						novel
23764	221223	MAFF	CES5A	none						novel
23764	874	MAFF	CBR3	none						novel
25800	874	SLC39A6	CBR3	none						novel
25800	6514	SLC39A6	SLC2A2	none						novel
25800	2949	SLC39A6	GSTM5	none						novel

From (EntrezID)	To (EntrezID)	From (name)	To (name)	Interaction	Intermediate	Intermediate type	Interaction type: gene regulatory and . .	Intermediates (EntrezID)	In gene list?	Conclusion
25800	7295	SLC39A6	TXN	none						novel
25800	131	SLC39A6	ADH7	indirect	Zn2+	compound	BI and PI		no	TP
25800	8714	SLC39A6	ABCC3	none						novel
25800	8824	SLC39A6	CES2	indirect	MAX	gene/protein	---	4149	no	TP
28968	8824	SLC6A16	CES2	none						novel
28968	873	SLC6A16	CBR1	none						novel
29985	81031	SLC39A3	SLC2A10	none						novel
29985	4097	SLC39A3	MAFG	none						novel
29985	2877	SLC39A3	GPX2	none						novel
29985	2539	SLC39A3	G6PD	none						novel
29985	2495	SLC39A3	FTH1	none						novel
29985	131	SLC39A3	ADH7	indirect	Zn2+	compound	BI and PI		no	TP
29986	6526	SLC39A2	SLC5A3	none						novel
29986	5467	SLC39A2	PPARD	none						novel
29988	9152	SLC2A8	SLC6A5	indirect	Na+	compound	BI		no	TP
29988	9817	SLC2A8	KEAP1	indirect	MAX<br>PRKAG1	gene/protein	PI	4149<br>5571	no<br>no	TP
29988	2877	SLC2A8	GPX2	none						novel
29988	6526	SLC2A8	SLC5A3	direct			BI			TP
29988	873	SLC2A8	CBR1	none						novel
29988	8878	SLC2A8	SQSTM1	indirect	MAX	gene/protein	---	4149	no	TP
29988	5155	SLC2A8	PDGFB	none						novel
29988	4097	SLC2A8	MAFG	none						novel
29988	55630	SLC2A8	SLC39A4	none						novel
29988	54716	SLC2A8	SLC6A20	indirect	Na+	compound	BI		no	TP
29988	57181	SLC2A8	SLC39A10	none						novel
29988	201266	SLC2A8	SLC39A11	none						novel
29988	23764	SLC2A8	MAFF	indirect	MAX	gene/protein	---	4149	no	TP
29988	55117	SLC2A8	SLC6A15	indirect	Na+	compound	BI		no	TP
29988	221074	SLC2A8	SLC39A12	none						novel
54578	6513	UGT1A6	SLC2A1	none						novel
54716	1958	SLC6A20	EGR1	none						novel
54716	2877	SLC6A20	GPX2	none						novel
54716	6256	SLC6A20	RXRA	none						novel
55117	1839	SLC6A15	HBEGF	none						novel
55117	3303	SLC6A15	HSPA1A	none						novel
55117	7295	SLC6A15	TXN	none						novel
55334	2042	SLC39A9	EPHA3	none						novel
55630	2949	SLC39A4	GSTM5	none						novel
55630	873	SLC39A4	CBR1	none						novel
55630	283375	SLC39A4	SLC39A5	direct			BI			TP
55630	3303	SLC39A4	HSPA1A	none						novel
56606	2729	SLC2A9	GCLC	none						novel
56606	2495	SLC2A9	FTH1	none						novel
56606	81031	SLC2A9	SLC2A10	direct			BI			TP
56606	8714	SLC2A9	ABCC3	none						novel
56606	3303	SLC2A9	HSPA1A	indirect	SLC5A1	gene/protein	BI and PI	6523	yes	FP
56606	29988	SLC2A9	SLC2A8	direct			BI			TP
57181	1839	SLC39A10	HBEGF	none						novel
57181	81031	SLC39A10	SLC2A10	none						novel
57181	2729	SLC39A10	GCLC	none						novel
57181	8824	SLC39A10	CES2	none						novel
57181	7048	SLC39A10	TGFBR2	indirect	CREB1 -><br>FL1	gene/protein	PI	1385 -><br>2313	no<br>no	TP
57181	6524	SLC39A10	SLC5A2	none						novel
57181	340024	SLC39A10	SLC6A19	none						novel
57181	2730	SLC39A10	GCLM	none						novel
57181	873	SLC39A10	CBR1	none						novel
57181	1958	SLC39A10	EGR1	indirect	CREB1	gene/protein	PI	1385	no	TP
60482	3326	SLC5A7	HSP90AB1	none						novel

From (EntrezID)	To (EntrezID)	From (name)	To (name)	Interaction	Intermediate	Intermediate type	Interaction type: gene regulatory and …	Intermediates (EntrezID)	In gene list?	Conclusion
64116	2042	SLC39A8	EPHA3	none						novel
64116	4780	SLC39A8	NFE2L2	none						novel
64116	6526	SLC39A8	SLC5A3	none						novel
64116	6534	SLC39A8	SLC6A7	none						novel
64116	2877	SLC39A8	GPX2	none						novel
64116	2938	SLC39A8	GSTA1	none						novel
64116	81031	SLC39A8	SLC2A10	none						novel
64116	6649	SLC39A8	SOD3	none						novel
64116	2949	SLC39A8	GSTM5	none						novel
64116	283375	SLC39A8	SLC39A5	direct			BI			TP
66035	3326	SLC2A11	HSP90AB1	none						novel
66035	8884	SLC2A11	SLC5A6	indirect	Na+	compound	BI		no	TP
66035	873	SLC2A11	CBR1	none						novel
66035	81031	SLC2A11	SLC2A10	direct			BI			TP
66035	64116	SLC2A11	SLC39A8	none						novel
66035	4780	SLC2A11	NFE2L2	none						novel
66035	283375	SLC2A11	SLC39A5	none						novel
81031	873	SLC2A10	CBR1	none						novel
81031	2940	SLC2A10	GSTA3	none						novel
81031	2730	SLC2A10	GCLM	none						novel
81031	201266	SLC2A10	SLC39A11	none						novel
81031	2877	SLC2A10	GPX2	none						novel
91252	1839	SLC39A13	HBEGF	none						novel
91252	3337	SLC39A13	DNAJB1	none						novel
91252	4780	SLC39A13	NFE2L2	none						novel
91252	28968	SLC39A13	SLC6A16	none						novel
91252	5155	SLC39A13	PDGFB	none						novel
91252	10257	SLC39A13	ABCC4	none						novel
91252	8884	SLC39A13	SLC5A6	none						novel
91252	2729	SLC39A13	GCLC	none						novel
91252	6514	SLC39A13	SLC2A2	none						novel
91252	2877	SLC39A13	GPX2	none						novel
91252	4097	SLC39A13	MAFG	none						novel
91252	23657	SLC39A13	SLC7A11	none						novel
91252	3320	SLC39A13	HSP90AA1	none						novel
91252	201266	SLC39A13	SLC39A11	direct			BI			TP
91252	221074	SLC39A13	SLC39A12	direct			BI			TP
91252	7040	SLC39A13	TGFB1	none						novel
91252	29985	SLC39A13	SLC39A3	direct			BI			TP
91252	2495	SLC39A13	FTH1	none						novel
91252	3303	SLC39A13	HSPA1A	indirect	REL	gene/protein	PI	5966	no	TP
91252	1958	SLC39A13	EGR1	none						novel
91252	9588	SLC39A13	PRDX6	none						novel
91252	81031	SLC39A13	SLC2A10	none						novel
91252	57181	SLC39A13	SLC39A10	direct			BI			TP
114112	4199	TXNRD3	ME1	indirect	NADPH	compound	BI		no	TP
114112	23516	TXNRD3	SLC39A14	none						novel
114112	221223	TXNRD3	CES5A	none						novel
114112	283848	TXNRD3	CES4A	none						novel
114112	5467	TXNRD3	PPARD	none						novel
114112	4780	TXNRD3	NFE2L2	indirect	TXNRD1	gene/protein	BI	7296	yes	FP
114112	1839	TXNRD3	HBEGF	none						novel
114112	6515	TXNRD3	SLC2A3	none						novel
114112	10057	TXNRD3	ABCC5	none						novel
114134	9817	SLC2A13	KEAP1	none						novel
114134	5155	SLC2A13	PDGFB	none						novel
114134	57181	SLC2A13	SLC39A10	none						novel
114134	25800	SLC2A13	SLC39A6	none						novel
114134	3303	SLC2A13	HSPA1A	none						novel
114134	1958	SLC2A13	EGR1	none						novel

From (EntrezID)	To (EntrezID)	From (name)	To (name)	Interaction	Intermediate	Intermediate type	Interaction type: gene regulatory and …	Intermediates (EntrezID)	In gene list?	Conclusion
114134	6526	SLC2A13	SLC5A3	indirect	Ins	compound	BI		no	TP
115584	7048	SLC5A11	TGFBR2	none						novel
115584	6256	SLC5A11	RXRA	none						novel
115584	91252	SLC5A11	SLC39A13	none						novel
115584	2949	SLC5A11	GSTM5	none						novel
115584	3303	SLC5A11	HSPA1A	none						novel
115584	283375	SLC5A11	SLC39A5	none						novel
115584	1958	SLC5A11	EGR1	none						novel
115584	10257	SLC5A11	ABCC4	none						novel
115584	10057	SLC5A11	ABCC5	none						novel
115584	873	SLC5A11	CBR1	none						novel
115584	1548	SLC5A11	CYP2A6	none						novel
115584	8714	SLC5A11	ABCC3	none						novel
125206	2949	SLC5A10	GSTM5	none						novel
125206	2512	SLC5A10	FTL	none						novel
125206	23657	SLC5A10	SLC7A11	none						novel
125206	7295	SLC5A10	TXN	none						novel
125206	10057	SLC5A10	ABCC5	none						novel
125206	283375	SLC5A10	SLC39A5	none						novel
125206	1958	SLC5A10	EGR1	none						novel
125206	115584	SLC5A10	SLC5A11	indirect	SLC5A3<br>Na+	gene/protein<br>compound	BI	6526<br>---	yes<br>no	TP
125206	1839	SLC5A10	HBEGF	none						novel
125206	131	SLC5A10	ADH7	none						novel
125206	6534	SLC5A10	SLC6A7	indirect	Na+	compound	BI		no	TP
125206	874	SLC5A10	CBR3	none						novel
125206	2948	SLC5A10	GSTM4	none						novel
125206	8824	SLC5A10	CES2	none						novel
154091	7048	SLC2A12	TGFBR2	none						novel
154091	6540	SLC2A12	SLC6A13	indirect	Na+	compound	BI		no	TP
154091	2729	SLC2A12	GCLC	none						novel
154091	60482	SLC2A12	SLC5A7	indirect	Na+	compound	BI		no	TP
154091	57181	SLC2A12	SLC39A10	none						novel
154091	1969	SLC2A12	EPHA2	indirect	TP53	gene/protein	PI	7157	no	TP
154091	3320	SLC2A12	HSP90AA1	indirect	SLC2A4	gene/protein	BI and PI	6517	yes	FP
154091	125206	SLC2A12	SLC5A10	direct			BI			TP
154091	10257	SLC2A12	ABCC4	none						novel
154091	81031	SLC2A12	SLC2A10	direct			BI			TP
154091	3303	SLC2A12	HSPA1A	indirect	SLC5A1	gene/protein	BI and PI	6523	yes	FP
154091	2495	SLC2A12	FTH1	none						novel
154091	1958	SLC2A12	EGR1	none						novel
159963	3303	SLC5A12	HSPA1A	indirect	SLC5A1	gene/protein	BI and PI	6523	yes	FP
159963	874	SLC5A12	CBR3	none						novel
159963	2949	SLC5A12	GSTM5	none						novel
159963	1958	SLC5A12	EGR1	none						novel
159963	8714	SLC5A12	ABCC3	none						novel
160728	10057	SLC5A8	ABCC5	none						novel
160728	8714	SLC5A8	ABCC3	none						novel
160728	1958	SLC5A8	EGR1	none						novel
200010	114134	SLC5A9	SLC2A13	none						novel
200010	8824	SLC5A9	CES2	none						novel
200010	25800	SLC5A9	SLC39A6	none						novel
201266	55630	SLC39A11	SLC39A4	direct			BI			TP
201266	1958	SLC39A11	EGR1	none						novel
201266	6538	SLC39A11	SLC6A11	none						novel
201266	2877	SLC39A11	GPX2	none						novel
201266	1969	SLC39A11	EPHA2	none						novel
201266	8884	SLC39A11	SLC5A6	none						novel
201266	874	SLC39A11	CBR3	none						novel
201266	221223	SLC39A11	CES5A	none						novel

From (EntrezID)	To (EntrezID)	From (name)	To (name)	Interaction	Intermediate	Intermediate type	Interaction type: gene regulatory and …	Intermediates (EntrezID)	In gene list?	Conclusion
201266	2949	SLC39A11	GSTM5	none						novel
201266	8824	SLC39A11	CES2	none						novel
221074	10057	SLC39A12	ABCC5	none						novel
221074	4780	SLC39A12	NFE2L2	none						novel
221074	8714	SLC39A12	ABCC3	none						novel
221074	6256	SLC39A12	RXRA	indirect	Zn2+	compound	BI and PI		no	TP
221074	1958	SLC39A12	EGR1	none						novel
221074	1839	SLC39A12	HBEGF	none						novel
221223	6514	CES5A	SLC2A2	none						novel
221223	4780	CES5A	NFE2L2	none						novel
221223	6535	CES5A	SLC6A8	none						novel
221223	4259	CES5A	MGST3	none						novel
221223	1958	CES5A	EGR1	none						novel
221223	23516	CES5A	SLC39A14	none						novel
221223	1839	CES5A	HBEGF	none						novel
221223	8714	CES5A	ABCC3	none						novel
283375	4780	SLC39A5	NFE2L2	none						novel
283375	55117	SLC39A5	SLC6A15	none						novel
283375	125206	SLC39A5	SLC5A10	none						novel
283375	1839	SLC39A5	HBEGF	none						novel
283375	6526	SLC39A5	SLC5A3	none						novel
283375	4097	SLC39A5	MAFG	none						novel
283848	873	CES4A	CBR1	none						novel
283848	7295	CES4A	TXN	none						novel
283848	8824	CES4A	CES2	direct			BI			TP
283848	3303	CES4A	HSPA1A	none						novel
283848	1958	CES4A	EGR1	none						novel
340024	2877	SLC6A19	GPX2	none						novel
348932	3303	SLC6A18	HSPA1A	none						novel
348932	8884	SLC6A18	SLC5A6	indirect	Na+	compound	BI		no	TP
388662	1958	SLC6A17	EGR1	none						novel

Table S5-2: Pathways (q-value ≤ 0.05) in ConsensusPathDB related to the network in Figure S5-1. Disease pathways of non-liver diseases were removed from the table.

pathway	database	q-value
NRF2 pathway	Wikipathways	1.9E-279
Nuclear Receptors Meta-Pathway	Wikipathways	1.8E-197
Transport of glucose and other sugars, bile salts and organic acids, metal ions and amine compounds	Reactome	1.02E-51
SLC-mediated transmembrane transport	Reactome	2.44E-38
Transmembrane transport of small molecules	Reactome	9.85E-31
Fructose and mannose metabolism	EHMN	2.63E-29
Na+/Cl- dependent neurotransmitter transporters	Reactome	2.25E-19
Glutathione metabolism - Homo sapiens (human)	KEGG	5.26E-19
Facilitative Na+-independent glucose transporters	Reactome	6.24E-19
Amine compound SLC transporters	Reactome	8.55E-18
Galactose metabolism	EHMN	5.25E-17
Metabolism of xenobiotics by cytochrome P450 - Homo sapiens (human)	KEGG	1.97E-15
Zinc influx into cells by the SLC39 gene family	Reactome	2.67E-15
Glutathione conjugation	Reactome	7.36E-14
Chemical carcinogenesis - Homo sapiens (human)	KEGG	2.27E-13
Drug metabolism - cytochrome P450 - Homo sapiens (human)	KEGG	3.79E-13
Inositol transporters	Reactome	1.48E-12
glutathione-mediated detoxification	HumanCyc	4.12E-12
Zinc transporters	Reactome	4.12E-12
Detoxification of Reactive Oxygen Species	Reactome	1.96E-10
Metal ion SLC transporters	Reactome	2.88E-10
Phase II conjugation	Reactome	5.68E-10
Biological oxidations	Reactome	6.09E-10
Oxidative Stress	Wikipathways	8.25E-10
Metabolism	Reactome	1.32E-09
Glutathione metabolism	Wikipathways	1.55E-09
Transcriptional activation by NRF2	Wikipathways	2.96E-09
Hexose transport	Reactome	4.07E-09
Na+-dependent glucose transporters	Reactome	3.55E-08
4-hydroxy-2-nonenal detoxification	HumanCyc	4.75E-07
oxidative stress induced gene expression via nrf2	BioCarta	4.88E-07
Constitutive Androstane Receptor Pathway	Wikipathways	5.65E-07
Pregnane X Receptor pathway	Wikipathways	7.23E-07
Amino acid and oligopeptide SLC transporters	Reactome	2.17E-06
Metapathway biotransformation	Wikipathways	6.22E-06
Urea cycle and metabolism of arginine, proline, glutamate, aspartate and asparagine	EHMN	8.34E-06
Aryl Hydrocarbon Receptor Pathway	Wikipathways	8.96E-06
Amino acid transport across the plasma membrane	Reactome	2.56E-05
Vitamin C metabolism	EHMN	2.9E-05
Class II GLUTs	Reactome	2.9E-05
Selenium Micronutrient Network	Wikipathways	5.22E-05
Reuptake of GABA	Reactome	0.000105
Arachidonic acid metabolism	EHMN	0.000176
Metabolism of carbohydrates	Reactome	0.000183
Arylhydrocarbon receptor (AhR) signaling pathway	Wikipathways	0.000188
Tyrosine metabolism	EHMN	0.000195
Transport of inorganic cations/anions and amino acids/oligopeptides	Reactome	0.000208
Heme degradation	Reactome	0.000219
Quercetin and Nf-kB- AP-1 Induced Cell Apoptosis	Wikipathways	0.000236
Cori Cycle	Wikipathways	0.000236
Mineral absorption - Homo sapiens (human)	KEGG	0.000236
ErbB receptor signaling network	PID	0.000312
Fluoropyrimidine Activity	Wikipathways	0.000337
Androgen and estrogen biosynthesis and metabolism	EHMN	0.000354
Monoamine Transport	Wikipathways	0.000378
Prostaglandin formation from arachidonate	EHMN	0.000436
Selenium Metabolism and Selenoproteins	Wikipathways	0.000572
glutathione redox reactions I	HumanCyc	0.000611
Xenobiotics metabolism	EHMN	0.000842
Creatine metabolism	Reactome	0.001336
reactive oxygen species degradation	HumanCyc	0.001336
TP53 Regulates Metabolic Genes	Reactome	0.001779
Transcriptional Regulation by TP53	Reactome	0.001779
Bile secretion - Homo sapiens (human)	KEGG	0.001779
Cellular responses to stress	Reactome	0.001804

Glycolysis and Gluconeogenesis	Wikipathways	0.002126
Attenuation phase	Reactome	0.002407
Extracellular vesicle-mediated signaling in recipient cells	Wikipathways	0.003156
glutathione biosynthesis	HumanCyc	0.003281
Estrogen metabolism	Wikipathways	0.003404
HSF1 activation	Reactome	0.003404
Arachidonic acid metabolism - Homo sapiens (human)	KEGG	0.003412
Fatty Acid Omega Oxidation	Wikipathways	0.004147
Nuclear Receptors in Lipid Metabolism and Toxicity	Wikipathways	0.004675
HSF1-dependent transactivation	Reactome	0.005319
heme degradation	HumanCyc	0.005319
pentose phosphate pathway (oxidative branch)	HumanCyc	0.005319
TGFBR2 Kinase Domain Mutants in Cancer	Reactome	0.005319
Loss of Function of TGFBR2 in Cancer	Reactome	0.005319
TGFBR1 KD Mutants in Cancer	Reactome	0.005319
Loss of Function of TGFBR1 in Cancer	Reactome	0.005319
thioredoxin pathway	HumanCyc	0.005319
Metabolism of porphyrins	Reactome	0.005386
retinol biosynthesis	HumanCyc	0.005386
Leukotriene metabolism	EHMN	0.006247
Synthesis and interconversion of nucleotide di- and triphosphates	Reactome	0.006247
Drug Induction of Bile Acid Pathway	Wikipathways	0.006247
basic mechanism of action of ppara pparb(d) and pparg and effects on gene expression	BioCarta	0.007579
Codeine and Morphine Metabolism	Wikipathways	0.007579
Glucose transport	Reactome	0.007579
GABA synthesis, release, reuptake and degradation	Reactome	0.007952
ABC-family proteins mediated transport	Reactome	0.008183
TGF-beta signaling TAK1	INOH	0.009048
tgf beta signaling pathway	BioCarta	0.009048
ABC transporters - Homo sapiens (human)	KEGG	0.009548
Metabolism of amino acids and derivatives	Reactome	0.00967
gamma-glutamyl cycle	HumanCyc	0.010227
multi-drug resistance factors	BioCarta	0.010227
SMAD2/3 Phosphorylation Motif Mutants in Cancer	Reactome	0.010227
SMAD2/3 MH2 Domain Mutants in Cancer	Reactome	0.010227
Loss of Function of SMAD2/3 in Cancer	Reactome	0.010227
nicotine degradation III	HumanCyc	0.010227
Retinol metabolism - Homo sapiens (human)	KEGG	0.010632
Aryl Hydrocarbon Receptor	Wikipathways	0.01096
Platelet degranulation	Reactome	0.011718
ctcf: first multivalent nuclear factor	BioCarta	0.011953
Arachidonic acid metabolism	Reactome	0.012552
Pentose Phosphate Pathway	Wikipathways	0.012761
Pentose phosphate pathway (hexose monophosphate shunt)	Reactome	0.012761
Signaling by TGF-beta Receptor Complex in Cancer	Reactome	0.012761
Phase I biotransformations, non P450	Wikipathways	0.012761
Pathways in cancer - Homo sapiens (human)	KEGG	0.013952
Neurotransmitter Release Cycle	Reactome	0.013989
Glypican 1 network	PID	0.014384
Response to elevated platelet cytosolic Ca2+	Reactome	0.014384
Apoptosis-related network due to altered Notch3 in ovarian cancer	Wikipathways	0.014384
mechanism of gene regulation by peroxisome proliferators via ppara	BioCarta	0.014384
Glutamate Glutamine metabolism	INOH	0.014384
RXR and RAR heterodimerization with other nuclear receptor	PID	0.014384
Vitamin C (ascorbate) metabolism	Reactome	0.014384
Organic anion transporters	Reactome	0.014384
alk in cardiac myocytes	BioCarta	0.014384
Prostate cancer - Homo sapiens (human)	KEGG	0.014384
nfkb activation by nontypeable hemophilus influenzae	BioCarta	0.014384
Porphyrin and chlorophyll metabolism - Homo sapiens (human)	KEGG	0.015386
Glutathione synthesis and recycling	Reactome	0.017316
The NLRP3 inflammasome	Reactome	0.017316
pentose phosphate pathway	HumanCyc	0.017316
Import of palmitoyl-CoA into the mitochondrial matrix	Reactome	0.017316
Irinotecan Pathway	Wikipathways	0.017316
Drug metabolism - other enzymes - Homo sapiens (human)	KEGG	0.017534
EPHA-mediated growth cone collapse	Reactome	0.021768
Senescence and Autophagy in Cancer	Wikipathways	0.023351

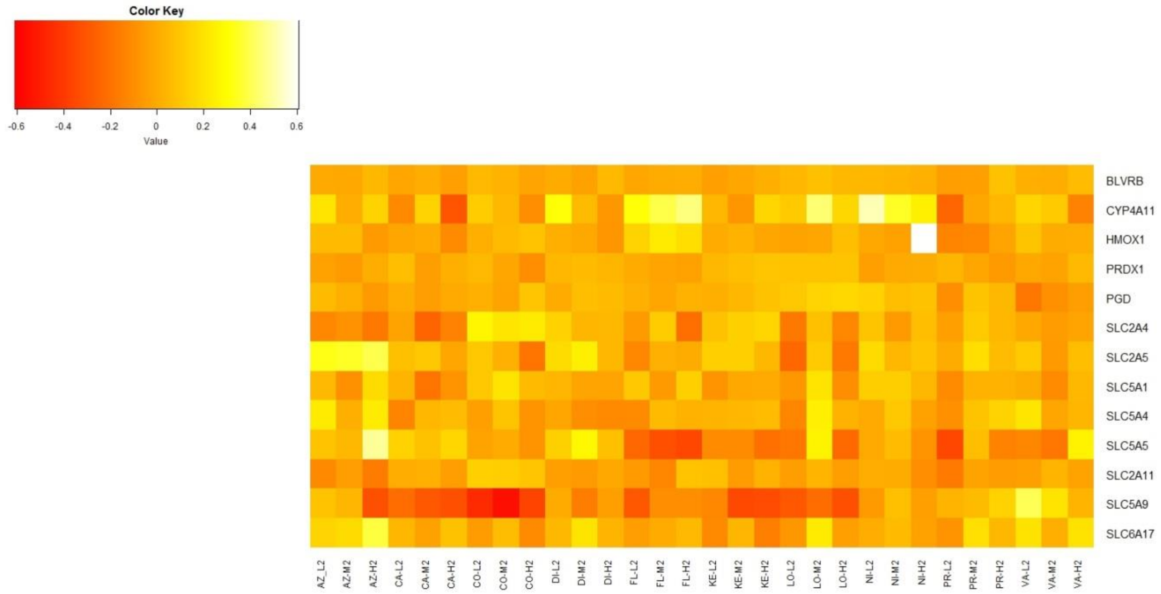

Figure S5-2: Heatmap for the 13 start nodes (nodes with only outgoing edges) in the network of Figure S5-1, displaying the log2 ratios at the earliest time point (2 hours). AZ = azathioprine; CA = carbamazepine; CO = coumarin; DI = diazepam; FL = flutamide; KE = ketoconazole; LO = lomustine; NI = nitrofurantoin; PR = propylthiouracil; VA = valproic acid; L = low dose; M = middle dose; H = high dose.

Table S5-3: Function of the genes in the network of Figure S5-1, extracted from GeneCards.

Entrez ID	Gene name	function
1244	ABCC2	drug resistance, transporter activity
8714	ABCC3	transporter activity, multi-drug resistance, metabolism
10257	ABCC4	transporter activity, multi-drug resistance, signaling
10057	ABCC5	transporter activity, multi-drug resistance, metabolism, glycosaminoglycan metabolism
131	ADH7	signaling, metabolism
177	AGER	immune system, signaling, oxidative stress
218	ALDH3A1	metabolism of corticosteroids, biogenic amines, neurotransmitters, and lipid peroxidation
645	BLVRB	metabolism, porphyrin and chlorophyll metabolism
873	CBR1	metabolism
874	CBR3	metabolism
8824	CES2	metabolism, fatty acyl and cholesterol ester metabolism, drug metabolism -cytochrome P450
23491	CES3	fatty acyl and cholesterol ester metabolism, detoxification of xenobiotics
283848	CES4A	fatty acyl and cholesterol ester metabolism, detoxification of drugs and xenobiotics
221223	CES5A	fatty acyl and cholesterol ester metabolism, detoxification of xenobiotics, activation of ester and amide prodrugs.
1548	CYP2A6	metabolism, drug metabolism - cytochrome P450
1579	CYP4A11	metabolism, lipid metabolism, drug metabolism - cytochrome P450
3337	DNAJB1	protein folding, signaling
1958	EGR1	immune system
1969	EPHA2	signaling, cell adhesion, cell proliferation and differentiation, apoptosis, cell migration
2042	EPHA3	development, signaling, cell-cell adhesion, cytoskeletal organization, cell migration
2258	FGF13	development, cell growth, morphogenesis, tissue repair, tumor growth, signaling
2495	FTH1	transporter activity
2512	FTL	transporter activity
2539	G6PD	metabolism
2729	GCLC	metabolism, drug metabolism - phase II conjugation
2730	GCLM	metabolism, drug metabolism - phase II conjugation
2877	GPX2	metabolism
2878	GPX3	cellular senescence, folate metabolism, protection against oxidative DNA damage
2936	GSR	metabolism
2938	GSTA1	metabolism, phase II conjugation
2940	GSTA3	metabolism, phase II conjugation
2941	GSTA4	metabolism, phase II conjugation
2947	GSTM3	metabolism, phase II conjugation
2948	GSTM4	detoxification of xenobiotics, metabolism, phase II conjugation
2949	GSTM5	metabolism, phase II conjugation
2950	GSTP1	metabolism, cellular senescence
1839	HBEGF	signaling, immune system
3162	HMOX1	metabolism, transporter activity
3320	HSP90AA1	cell signaling, inflammatory response
3326	HSP90AB1	signal transduction, protein folding, protein degradation, morphological evolution, apoptosis, inflammation
3303	HSPA1A	inflammatory response
9817	KEAP1	immune system
23764	MAFF	cellular stress response, signaling
4097	MAFG	signaling
4199	ME1	metabolism, lipid metabolism
4258	MGST2	inflammation, metabolism, phase II conjugation
4259	MGST3	inflammation, metabolism, phase II conjugation
4780	NFE2L2	inflammatory response, signaling
1728	NQO1	metabolism
3084	NRG1	cell-cell signaling, development
5155	PDGFB	signaling, development, cell proliferation, cell migration, survival, chemotaxis, angiogenesis, cell differentiation
5226	PGD	metabolism, glycosaminoglycan metabolism
5467	PPARD	signaling, lipid metabolism
5052	PRDX1	cellular senescence, redox regulation of the cell, signaling
9588	PRDX6	redox regulation of the cell, protection against oxidative injury, metabolism, cellular senescence
22949	PTGR1	metabolism, arachidonic acid metabolism
6256	RXRA	signaling
5265	SERPINA1	signaling
6513	SLC2A1	metabolism, glycosaminoglycan metabolism, transporter activity
81031	SLC2A10	transporter activity, regulation of glucose homeostatis
66035	SLC2A11	transporter activity
154091	SLC2A12	transporter activity
114134	SLC2A13	transporter activity

6514	SLC2A2	metabolism, glycosaminoglycan metabolism, transporter activity
6515	SLC2A3	metabolism, glycosaminoglycan metabolism, transporter activity
6517	SLC2A4	metabolism, glycosaminoglycan metabolism, transporter activity
6518	SLC2A5	metabolism, glycosaminoglycan metabolism, transporter activity
11182	SLC2A6	transporter activity
29988	SLC2A8	transporter activity
56606	SLC2A9	transporter activity
57181	SLC39A10	protein, nucleic acid, carbohydrate, lipid metabolism; gene transcription, growth, development, and differentiation
201266	SLC39A11	transporter activity
221074	SLC39A12	transporter activity
91252	SLC39A13	transporter activity
23516	SLC39A14	transporter activity
29986	SLC39A2	transporter activity, senescence, autophagy, tumorigenesis
29985	SLC39A3	transporter activity, senescence, autophagy
55630	SLC39A4	transporter activity, senescence, autophagy
283375	SLC39A5	transporter activity
25800	SLC39A6	transporter activity
7922	SLC39A7	transporter activity
64116	SLC39A8	transporter activity, inflammation
55334	SLC39A9	transporter activity
6523	SLC5A1	metabolism, glycosaminoglycan metabolism, transporter activity
125206	SLC5A10	transporter activity
115584	SLC5A11	transporter activity, apoptosis
159963	SLC5A12	transporter activity
6524	SLC5A2	metabolism, glycosaminoglycan metabolism, transporter activity
6526	SLC5A3	metabolism, glycosaminoglycan metabolism, transporter activity
6527	SLC5A4	metabolism, glycosaminoglycan metabolism, transporter activity
6528	SLC5A5	metabolism, transporter activity
8884	SLC5A6	metabolism, transporter activity
60482	SLC5A7	transporter activity
160728	SLC5A8	transporter activity, tumor suppressor
200010	SLC5A9	metabolism, glycosaminoglycan metabolism, transporter activity
6529	SLC6A1	transporter activity, circadian entrainment
6538	SLC6A11	metabolism, transporter activity
6540	SLC6A13	transporter activity, circadian entrainment
55117	SLC6A15	transporter activity
28968	SLC6A16	transporter activity
388662	SLC6A17	transporter activity
348932	SLC6A18	transporter activity
340024	SLC6A19	transporter activity
6530	SLC6A2	signaling, transporter activity
54716	SLC6A20	transporter activity, signaling
6531	SLC6A3	transporter activity
6532	SLC6A4	signaling, transporter activity
9152	SLC6A5	signaling, transporter activity
6533	SLC6A6	transporter activity
6534	SLC6A7	metabolism, transporter activity
6535	SLC6A8	metabolism, transporter activity
6536	SLC6A9	transporter activity
23657	SLC7A11	signaling, transporter activity
6649	SOD3	cellular senescence, folate metabolism
8878	SQSTM1	signaling, immune system, cell differentiation, apoptosis
7039	TGFA	signaling, cell proliferation, cell differentiation, development
7040	TGFB1	cell proliferation, cell differentiation, cell adhesion, cell migration, signaling
7042	TGFB2	cell proliferation, cell differentiation, cell adhesion, cell migration, signaling
7048	TGFBR2	cell proliferation, signaling
7295	TXN	metabolism
7296	TXNRD1	selenium metabolism, protection against oxidative stress, metabolism
114112	TXNRD3	defense against oxidative stress
54578	UGT1A6	metabolism, phase II conjugation, elimination of xenobiotics

Table S5-4: Unknown interactions in CPDB – putative functional relationships inferred with Biograph, based on known interactions. Gene pairs without functional relationships in BioGraph were omitted from the table.

from	to	putative functional relations	type of interactions
CBR3	PPARD	CBR3 -> GABARAPL1 -> DUT -> PPARD<br>CBR3 -> GABARAP -> DUT -> PPARD	protein interaction, protein interaction, protein interaction<br>protein interaction, protein interaction, protein interaction
CBR3	GSTM3	CBR3 -> GABARAPL1 -> ATG5 -> GSTM3<br>CBR3 -> USP3 -> Water -> GSTM3	protein interaction, protein interaction, protein interaction<br>protein interaction, reaction, reaction
CBR3	NQO1	CBR3 -> NADP -> NQO1	reaction, reaction
CBR3	SERPINA1	CBR3 -> USP3 -> LRP1 -> SERPINA1<br>CBR3 -> USP3 -> UBC -> SERPINA1<br>CBR3 -> SGK1 -> UBC -> SERPINA1<br>CBR3 -> ARIH2 -> UBC -> SERPINA1<br>CBR3 -> GABARAPL1 -> CANX -> SERPINA1	protein interaction, protein interaction, protein interaction<br>protein interaction, protein interaction, protein interaction<br>protein interaction, protein interaction, protein interaction<br>protein interaction, protein interaction, protein interaction<br>protein interaction, protein interaction, protein interaction
CBR3	PDGFB	CBR3 -> USP3 -> LRP1 -> PDGFB	protein interaction, protein interaction, protein interaction
CYP2A6	GPX2	CYP2A6 -> Water -> GPX2	reaction, reaction
CYP4A11	HSPA1A	CYP4A11 -> Oxygen -> MOS -> HSPA1A	reaction, expression, protein interaction
CYP4A11	SLC2A1	CYP4A11 -> Oxygen -> SLC2A1	reaction, reaction
EPHA2	FGF13	EPHA2 -> TIAM1 -> MAPK8IP2 -> FGF13	protein interaction, protein interaction, protein interaction
FTL	EPHA3	FTL -> MPHOSPH6 -> TP53 -> EPHA3	protein interaction, protein interaction, protein interaction
G6PD	CBR1	G6PD -> NADP -> CBR1	reaction, reaction
G6PD	TXNRD1	G6PD -> NADP -> TXNRD1	reaction, reaction
GPX2	ADH7	GPX2 -> water -> ADH7	reaction, reaction
GSTM3	NFE2L2	GSTM3 -> GRB2 -> NFE2L2	protein interaction, protein interaction
GSTM3	HBEGF	GSTM3 -> MPG -> FBLN1 -> HBEGF	protein interaction, protein interaction, protein interaction
GSTM3	EGR1	GSTM3 -> TFE3 -> PSMA3 -> EGR1	protein interaction, protein interaction, protein interaction
NRG1	TXN	NRG1 -> EGFR -> TXN	protein interaction, protein interaction
NRG1	PDGFB	NRG1 -> ADAM19 -> A2M -> PDGFB	protein interaction, protein interaction, protein interaction
HMOX1	GSTM5	HMOX1 -> water -> GSTM5	reaction, reaction
HMOX1	GPX3	HMOX1 -> water -> GPX3	reaction, reaction
HMOX1	GSTA4	HMOX1 -> water -> GSTA4	reaction, reaction
HSPA1A	MAFG	HSPA1A -> HCFC1 -> NFE2L1 -> MAFG	protein interaction, protein interaction, protein interaction
HSP90AB1	SLC6A9	HSP90AB1 -> MKNK1 -> PRKCA -> SLC6A9<br>HSP90AB1 -> RPS6 -> PRKCA -> SLC6A9<br>HSP90AB1 -> EEF2 -> PRKCA -> SLCA9<br>HSP90AB1 -> CFTR -> STX1A -> SLCA9	protein interaction, protein interaction, protein interaction<br>protein interaction, protein interaction, protein interaction<br>protein interaction, protein interaction, protein interaction<br>protein interaction, protein interaction, protein interaction
MAFG	SLC6A3	MAFG -> MED31 -> EPN1 -> SLC6A3<br>MAFG -> BACH1 -> PARK2 -> SLC6A3<br>MAFG -> BACH1 -> UBC -> SLC6A3<br>MAFG -> NFE2 -> NEDD4 -> SLC6A3<br>MAFG -> VIM -> STX1A -> SLC6A3	protein interaction, protein interaction, protein interaction<br>protein interaction, protein interaction, protein interaction<br>protein interaction, protein interaction, protein interaction<br>protein interaction, protein interaction, protein interaction<br>protein interaction, protein interaction, protein interaction
MAFG	PPARD	MAFG -> C7orf64 -> GADD45G -> PPARD<br>MAFG -> MED31 -> GADD45G -> PPARD	protein interaction, protein interaction, protein interaction<br>protein interaction, protein interaction, protein interaction
MAFG	SLC6A1	MAFG -> VIM -> STX1A -> SLC6A1	protein interaction, protein interaction, protein interaction
MAFG	SLC39A7	MAFG -> JUN -> CSNK2A2 -> SLC39A7<br>MAFG -> GTF2A1L -> CSNK2A2 -> SLC39A7<br>MAFG -> CREBBP -> CSNK2A2 -> SLC39A7	protein interaction, protein interaction, phosphorylation<br>protein interaction, protein interaction, phosphorylation<br>protein interaction, protein interaction, phosphorylation
MAFG	SERPINA1	MAFG -> BACH1 -> UBC -> SERPINA1	protein interaction, protein interaction, protein interaction
MAFG	HSPA1A	MAFG -> NFE2L1 -> HCFC1 -> HSPA1A	protein interaction, protein interaction, protein interaction
MAFG	SLC6A4	MAFG -> VIM -> STX1A -> SLC6A4<br>MAFG -> JUN -> CALR -> SLC6A4	protein interaction, protein interaction, protein interaction<br>protein interaction, protein interaction, protein interaction
PRDX1	GSTM5	PRDX1 -> water -> GSTM5	reaction, reaction
PDGFB	CBR3	PDGFB -> LRP1 -> USP3 -> CBR3<br>PDGFB -> HNRNPC -> GABARAPL1 -> CBR3<br>PDGFB -> HNRNPC -> GABARAP -> CBR3	protein interaction, protein interaction, protein interaction<br>protein interaction, protein interaction, protein interaction<br>protein interaction, protein interaction, protein interaction
PDGFB	NRG1	PDGFB -> A2M -> ADAM19 -> NRG1	protein interaction, protein interaction, protein interaction
SERPINA1	MAFG	SERPINA1 -> UBC -> BACH1 -> MAFG	protein interaction, protein interaction, protein interaction
SERPINA1	HSP90AB1	SERPINA1 -> CTRB1 -> RIPK1 -> HSP90AB1<br>SERPINA1 -> CANX -> RPL7 -> HSP90AB1	protein interaction, protein interaction, protein interaction<br>protein interaction, protein interaction, protein interaction
SERPINA1	CBR3	SERPINA1 -> LRP1 -> USP3 -> CBR3<br>SERPINA1 -> UBC -> USP3 -> CBR3<br>SERPINA1 -> UBC -> ARIH2 -> CBR3<br>SERPINA1 -> UBC -> SGK1 -> CBR3<br>SERPINA1 -> CANX -> GABARAPL1 -> CBR3	protein interaction, protein interaction, protein interaction<br>protein interaction, protein interaction, protein interaction<br>protein interaction, protein interaction, protein interaction<br>protein interaction, protein interaction, protein interaction<br>protein interaction, protein interaction, protein interaction
SERPINA1	TGFBR2	SERPINA1 -> UBC -> TGFBR2	protein interaction, protein interaction
SERPINA1	CBR1	SERPINA1 -> RAP2A -> GABARAPL2 -><br>CBR1	protein interaction, protein interaction, protein interaction<br>protein interaction, protein interaction, protein interaction

		SERPINA1 -> RAP2A -> TNIK -> CBR1<br>SERPINA1 -> SSR1 -> HLA-B -> CBR1<br>SERPINA1 -> SSR1 -> IKBKE -> CBR1<br>SERPINA1 -> CANX -> MAPK13 -> CBR1	protein interaction, protein interaction, protein interaction<br>protein interaction, protein interaction, protein interaction<br>protein interaction, protein interaction, protein interaction
SLC2A1	KEAP1	SLC2A1 -> STOM -> CUL2 -> KEAP1	protein interaction, protein interaction, protein interaction
SLC2A2	MAFG	SLC2A2 -> KPNA2 -> C7orf64 -> MAFG<br>SLC2A2 -> KPNA2 -> CHD3 -> MAFG<br>SLC2A2 -> KPNA2 -> CREBBP -> MAFG<br>SLC2A2 -> PRKCA -> NFE2L2 -> MAFG	protein interaction, protein interaction, protein interaction<br>protein interaction, protein interaction, protein interaction<br>protein interaction, protein interaction, protein interaction<br>phosphorylation, phosphorylation, phosphorylation
SLC2A2	CBR1	SLC2A2 -> KPNA2 -> UVRAG -> CBR1<br>SLC2A2 -> KPNA2 -> EPB41 -> CBR1	protein interaction, protein interaction, protein interaction<br>protein interaction, protein interaction, protein interaction
SLC5A1	TGFB2	SLC5A1 -> EGFR -> DCN -> TGFB2	protein interaction, protein interaction, protein interaction
SLC6A2	MAFG	SLC6A2 -> STX1A -> VIM -> MAFG	protein interaction, protein interaction, protein interaction
SLC6A8	HSPA1A	SLC6A8 -> CD59 -> CFTR -> HSPA1A	protein interaction, protein interaction, protein interaction
SLC6A8	HBEGF	SLC6A8 -> CD59 -> EGFR -> HBEGF<br>SLC6A8 -> CD59 -> CD9 -> HBEGF	protein interaction, protein interaction, protein interaction<br>protein interaction, protein interaction, protein interaction
SLC6A9	NFE2L2	SLC6A9 -> GABRR1 -> PRKCA -> PRKCD -> NFE2L2<br>SLC6A9 -> GABRR1 -> PRKCA -> RARA -> NFE2L2	protein interaction, protein interaction, protein interaction, protein interaction<br>protein interaction, protein interaction, protein interaction, protein interaction
SLC6A9	HSPA1A	SLC6A9 -> GABRR1 -> PRKCA -> HSPA1A<br>SLC6A9 -> GABRR1 -> PRKCA -> RICTOR -> HSPA1A<br>SLC6A9 -> IGHG1 -> KRT7 -> HSPA1A	protein interaction, protein interaction, protein interaction<br>protein interaction, protein interaction, protein interaction, protein interaction<br>protein interaction, protein interaction, protein interaction
SLC6A9	HBEGF	SLC6A9 -> PRKCA -> CD9 -> HBEGF	protein interaction, protein interaction, protein interaction
TGFB2	SLC2A2	TGFB2 -> VTN -> PRKACA -> SLC2A2	protein interaction, protein interaction, protein interaction
TXN	SLC2A2	TXN -> NCF4 -> PRKCA -> SLC2A2<br>TXN -> EPB41 -> KPNA2 -> SLCA2<br>TXN -> TXNIP -> KPNA2 -> SLCA2	protein interaction, phosphorylation, phosphorylation<br>protein interaction, protein interaction, protein interaction<br>protein interaction, protein interaction, protein interaction
TXN	NRG1	TXN -> EGFR -> NRG1	protein interaction, protein interaction
TXN	PPARD	TXN -> TMBIM4 -> PEBP1 -> PPARD	protein interaction, protein interaction, protein interaction
TXN	HBEGF	TXN -> EGFR -> HBEGF	protein interaction, protein interaction
TXNRD1	HBEGF	TXNRD1 -> ERBB4 -> HBEGF	phosphorylation, protein interaction
SLC39A7	CBR1	SLC39A7 -> CSNK1E -> MCC -> CBR1<br>SLC39A7 -> CD40 -> TRAF6 -> CBR1	phosphorylation, protein interaction, protein interaction<br>protein interaction, protein interaction, protein interaction
CES2	ALDH3A1	CES2 -> water -> ALDH3A1	reaction, reaction
KEAP1	CBR3	KEAP1 -> TMEM160 -> GABARAPL1 -> CBR3<br>KEAP1 -> TMEM160 -> GABARAP -> CBR3<br>KEAP1 -> GABARAP -> CBR3<br>KEAP1 -> PFAS -> SGK -> CBR3	protein interaction, protein interaction, protein interaction<br>protein interaction, protein interaction, protein interaction<br>protein interaction, protein interaction<br>protein interaction, protein interaction, protein interaction
KEAP1	GCLM	KEAP1 -> GET4 -> TNFRSF14 -> GCLM	protein interaction, protein interaction, protein interaction
PTGR1	ALDH3A1	PTGR1 -> NADP -> ALDH3A1<br>PTGR1 -> NADH -> ALDH3A1<br>PTGR1 -> NAD -> ALDH3A1	reaction, reaction<br>reaction, reaction<br>reaction, reaction
PTGR1	SLC2A1	PTGR1 -> NADH -> GAPDH -> SLC2A1<br>PTGR1 -> NAD -> GAPDH -> SLC2A1	reaction, reaction, protein interaction<br>reaction, reaction, protein interaction
PTGR1	HSPA1A	PTGR1 -> NADP -> NQO -> HSPA1A<br>PTGR1 -> NADH -> NQO -> HSPA1A<br>PTGR1 -> NAD -> NQO -> HSPA1A	reaction, reaction, protein interaction<br>reaction, reaction, protein interaction<br>reaction, reaction, protein interaction
MAFF	CBR3	MAFF -> HDAC5 -> GABARAP -> CBR3	protein interaction, protein interaction, protein interaction
SLC5A7	HSP90AB1	SLC5A7 -> PAWR -> PRKCZ -> HSP90AB1	protein interaction, protein interaction, protein interaction

Table S5-5: Top ten interactions in Biograph, based on absolute value of the interaction strength determined by DTNI – common function of the genes.

from	to	interaction strength	putative functional relations	common function of the genes
SLC6A9	HSPA1A	+ 0.0908	SLC6A9 -> GABRR1 -> PRKCA -> HSPA1A	inflammatory response: SLCA9 is involved in the transport of glycine, an amino acid known to reduce the inflammatory response (Grotz et al. 2001). GABRR1 codes for the protein GABA, a neurotransmitter known to reduce inflammation (Bhat et al. 2010). PRKCA and HSPA1A are both genes that play a role in the inflammatory response (source: GeneCards).
G6PD	TXNRD1	+ 0.0087	G6PD -> NADP -> TXNRD1	reaction with NADP
MAFG	SLC39A7	+ 0.0052	MAFG -> JUN -> CSNK2A2 -> SLC39A7	cell signalling: MAFG, JUN and CSNK2A are genes involved in cell signalling (source: GeneCards). SLC39A7 is involved in zinc transport, which is important for the activation of tyrosine kinases (source: GeneCards). Tyrosine kinases are involved in cell signalling (Lemmon and Schlessinger 2010).
G6PD	CBR1	+ 0.0038	G6PD -> NADP -> CBR1	reaction with NADP
SLC6A8	HSPA1A	+ 0.0032	SLC6A8 -> CD59 -> CFTR -> HSPA1A	cell signalling: SLC6A8 (creatine transporter) is regulated by tyrosine kinase. SLC6A8 and CD59 together regulate tyrosine phosphorylation (Wang et al. 2002). Tyrosine phosphorylation plays a role in cell signalling (Lemmon and Schlessinger 2010). HSPA1A also plays a role in signal transduction (Mayer and Bukau 2005).
TXN	HBEGF	- 0.0029	TXN -> EGFR -> HBEGF	immune system: TXN augments IL2 expression (source: GeneCards). EGFR and HBEGF are both involved in GPCR signalling (source: GeneCards). Both IL2 and GPCR have a role in the immune system.
HMOX1	GPX3	+ 0.0027	HMOX1 -> water -> GPX3	reaction with water
CBR3	NQO1	+ 0.0020	CBR3 -> NADP -> NQO1	reaction with NADP
CYP4A11	HSPA1A	- 0.0020	CYP4A11 -> Oxygen -> MOS -> HSPA1A	reaction with oxygen
PDGFB	NRG1	- 0.0018	PDGFB -> A2M -> ADAM19 -> NRG1	cell-cell communication (source: GeneCards)

Table S5-6: Unknown interactions in CPDB – gene pairs with functional interaction in STRING. Gene pairs without functional relationship in STRING were omitted from the table.

from	to	known/predicted/other?	type
EPHA2	FGF13	known	from curated databases
G6PD	TXNRD1	other	co-expression
GCLC	SLC7A11	other	text mining (PubMed)
GCLM	GSTA4	other	text mining (PubMed)
GCLM	ABCC5	other	text mining (PubMed)
GCLM	GSTA1	other	text mining (PubMed)
GCLM	SLC7A11	other	text mining (PubMed)
GSTA1	GCLM	other	text mining (PubMed)
HMOX1	GPX3	known	experimentally determined
SLC2A2	EPHA2	other	text mining (PubMed)
KEAP1	GCLM	other	text mining (PubMed)
SLC7A11	SLC6A6	other	text mining (PubMed)
TXNRD3	ABCC5	known	experimentally determined

Table S5-7: Unknown interactions in CPDB – relation of gene pairs with one (or more) of the ten studied compounds, extracted from CTD. Gene pairs that have no relationship with one of the compounds in CTD were omitted from the table.

from	to	relationship in CTD
BLVRB	GSTP1	both affected by flutamide and valproic acid
CBR1	GPX2	both affected by carbamazepine, flutamide and propylthiouracil
CBR3	SLC39A10	both affected by valproic acid
CBR3	SLC6A20	both affected by propylthiouracil and valproic acid
CBR3	ABCC2	both affected by propylthiouracil and valproic acid
CBR3	PPARD	both affected by propylthiouracil and valproic acid
CBR3	NRG1	both affected by propylthiouracil and valproic acid
CBR3	GSTM3	both affected by valproic acid
CBR3	SLC39A11	both affected by valproic acid
CBR3	SLC5A3	both affected by propylthiouracil and valproic acid
CBR3	SERPINA1	both affected by propylthiouracil and valproic acid
CBR3	GCLM	both affected by valproic acid
CBR3	PDGFB	both affected by propylthiouracil and valproic acid
ABCC2	CES2	both affected by ketoconazole, propylthiouracil and valproic acid
ABCC2	PPARD	both affected by propylthiouracil and valproic acid
ABCC2	SLC6A6	both affected by ketoconazole, propylthiouracil and valproic acid
ABCC2	CBR3	both affected by propylthiouracil and valproic acid
CYP2A6	SLC2A10	both affected by propylthiouracil and valproic acid
CYP2A6	SLC2A1	both affected by valproic acid
CYP2A6	SLC39A10	both affected by carbamazepine and valproic acid
CYP2A6	SLC6A6	both affected by valproic acid
CYP2A6	GPX2	both affected by carbamazepine
NQO1	SLC2A10	both affected by valproic acid
EGR1	SLC39A6	both affected by propylthiouracil and valproic acid
EGR1	PTGR1	both affected by propylthiouracil and valproic acid
EGR1	SLC39A5	both affected by valproic acid
EPHA2	GCLM	both affected by carbamazepine and valproic acid
EPHA2	GPX2	both affected by carbamazepine
EPHA2	FGF13	both affected by valproic acid
EPHA2	SLC6A6	both affected by valproic acid
EPHA2	SLC39A11	both affected by valproic acid
FGF13	SLC5A11	both affected by propylthiouracil and valproic acid
FGF13	ABCC3	both affected by propylthiouracil and valproic acid
FGF13	SLC2A1	both affected by valproic acid
FTH1	CBR1	both affected by carbamazepine, flutamide and valproic acid
FTH1	SLC2A10	both affected by valproic acid
FTH1	SLC6A1	both affected by valproic acid
FTL	SLC5A3	both affected by propylthiouracil and valproic acid
FTL	EPHA3	both affected by valproic acid
FTL	HBEGF	both affected by propylthiouracil and valproic acid
FTL	SLC6A7	both affected by propylthiouracil
FTL	SLC6A15	both affected by valproic acid
FTL	SLC7A11	both affected by propylthiouracil and valproic acid
G6PD	MGST2	both affected by flutamide and valproic acid
G6PD	CBR1	both affected by flutamide, ketoconazole and valproic acid
G6PD	FGF13	both affected by valproic acid
G6PD	TXNRD1	both affected by flutamide and valproic acid
G6PD	FTL	both affected by valproic acid
G6PD	SLC2A2	both affected by valproic acid
G6PD	KEAP1	both affected by flutamide and valproic acid
G6PD	SLC5A6	both affected by flutamide and valproic acid
G6PD	SLC39A11	both affected by valproic acid
GCLC	DNAJB1	both affected by carbamazepine and flutamide
GCLC	SLC7A11	both affected by carbamazepine and valproic acid
GCLM	SLC6A20	both affected by valproic acid
GCLM	GSTA4	both affected by coumarin, flutamide and valproic acid
GCLM	SLC39A10	both affected by carbamazepine and valproic acid
GCLM	CBR3	both affected by valproic acid
GCLM	GSTM5	both affected by coumarin and valproic acid
GCLM	PRDX6	both affected by flutamide and valproic acid
GCLM	ABCC5	both affected by flutamide and valproic acid
GCLM	GSTA1	both affected by azathioprine, carbamazepine and valproic acid

GCLM	SLC2A12	both affected by valproic acid
GCLM	SLC7A11	both affected by azathioprine, carbamazepine and valproic acid
GCLM	DNAJB1	both affected by carbamazepine and flutamide
GPX2	SLC7A11	both affected by azathioprine, carbamazepine and propylthiouracil
GPX2	EPHA2	both affected by carbamazepine
GPX2	SLC6A6	both affected by propylthiouracil
GPX2	TGFBR2	both affected by carbamazepine
GPX2	DNAJB1	both affacted by carbamazepine and flutamide
GPX2	HSP90AA1	both affected by carbamazepine, flutamide and propylthiouracil
GPX2	SLC39A8	both affected by flutamide and propylthiouracil
GPX2	SLC5A3	both affected by carbamazepine and propylthiouracil
GPX2	CBR1	both affected by carbamazepine, flutamide and propylthiouracil
GPX2	HSP90AB1	both affected by flutamide
GPX2	SLC5A6	both affected by flutamide
GPX2	ABCC4	both affected by carbamazepine, flutamide and propylthiouracil
GPX3	SLC5A6	both affected by valproic acid
GPX3	HSPA1A	both affected by valproic acid
GPX3	SLC2A1	both affected by valproic acid
GSR	SLC2A1	both affected by valproic acid
GSTA1	GCLM	both affected by azathioprine, carbamazepine and valproic acid
GSTA1	HSPA1A	both affected by carbamazepine and valproic acid
GSTA1	SLC2A1	both affected by valproic acid
GSTA4	PDGFB	both affected by propylthiouracil and valproic acid
GSTM3	NFE2L2	both affected by flutamide and valproic acid
GSTM3	HBEGF	both affected by valproic acid
GSTM3	CBR3	both affected by valproic acid
GSTM3	SLC2A13	both affected by valproic acid
GSTM3	EGR1	both affected by valproic acid
GSTM5	ABCC5	both affected by valproic acid
GSTM5	EGR1	both affected by valproic acid
GSTM5	ABCC3	both affected by valproic acid
GSTM5	SLC39A5	both affected by valproic acid
GSTM5	CBR1	both affected by coumarin and valproic acid
GSTM5	SLC5A11	both affected by valproic acid
GSTM5	HSPA1A	both affected by valproic acid
GSTM5	SLC5A12	both affected by valproic acid
GSTM5	SLC7A11	both affected by valproic acid
GSTP1	SLC39A11	both affected by valproic acid
GSTP1	MAFF	both affected by carbamazepine and valproic acid
NRG1	TXN	both affected by valproic acid
NRG1	PDGFB	both affected by propylthiouracil and valproic acid
NRG1	SLC6A6	both affected by propylthiouracil and valproic acid
NRG1	MAFG	both affected by flutamide and valproic acid
NRG1	CBR3	both affected by propylthiouracil and valproic acid
HMOX1	SLC6A6	both affected by ketoconazole and valproic acid
HMOX1	SLC39A2	both affected by carbamazepine
HMOX1	SLC39A10	both affected by carbamazepine and valproic acid
HMOX1	SLC39A11	both affected by valproic acid
HMOX1	GSTM5	both affected by valproic acid
HMOX1	GPX3	both affected by valproic acid
HMOX1	GSTA4	both affected by flutamide and valproic acid
HMOX1	SLC39A14	both affected by valproic acid
HMOX1	SLC2A10	both affected by valproic acid
HSPA1A	CES2	both affected by valproic acid
HSPA1A	SLC39A10	both affected by carbamazepine and valproic acid
HSPA1A	SLC5A6	both affected by valproic acid
HSPA1A	ABCC3	both affected by carbamazepine and valproic acid
HSPA1A	MAFG	both affected by valproic acid
HSPA1A	TGFB2	both affected by valproic acid
HSP90AA1	SLC39A10	both affected by carbamazepine and valproic acid
HSP90AA1	GPX2	both affected by carbamazepine
HSP90AB1	SLC6A9	both affected by flutamide
MAFG	SLC6A20	both affected by valproic acid
MAFG	PPARD	both affected by valproic acid
MAFG	PDGFB	both affected by valproic acid
MAFG	SLC6A1	both affected by valproic acid

MAFG	SLC39A7	both affected by valproic acid
MAFG	SERPINA1	both affected by flutamide and valproic acid
MAFG	SLC39A5	both affected by valproic acid
MAFG	SLC7A11	both affected by valproic acid
MAFG	HSPA1A	both affected by valproic acid
MAFG	ME1	both affected by flutamide and valproic acid
MAFG	NRG1	both affected by flutamide and valproic acid
ME1	SLC39A5	both affected by valproic acid
ME1	HSPA1A	both affected by carbamazepine and valproic acid
ME1	MAFG	both affected by flutamide and valproic acid
ME1	SLC6A1	both affected by valproic acid
ME1	NFE2L2	both affected by carbamazepine, flutamide and valproic acid
MGST2	SLC6A20	both affected by propylthiouracil and valproic acid
MGST3	SLC39A6	both affected by flutamide and valproic acid
MGST3	ABCC5	both affected by flutamide and valproic acid
NFE2L2	SLC39A2	both affected by carbamazepine
NFE2L2	SLC39A8	both affected by flutamide and valproic acid
NFE2L2	ABCC5	both affected by flutamide and valproic acid
NFE2L2	PDGFB	both affected by valproic acid
NFE2L2	TGFA	both affected by valproic acid
NFE2L2	SLC2A2	both affected by valproic acid
NFE2L2	CES2	both affected by ketoconazole and valproic acid
NFE2L2	SLC5A3	both affected by carbamazepine and valproic acid
NFE2L2	HBEGF	both affected by carbamazepine and valproic acid
PRDX1	GSTM5	both affected by valproic acid
PRDX1	SLC6A15	both affected by valproic acid
PRDX1	ABCC5	both affected by flutamide and valproic acid
PDGFB	MAFG	both affected by valproic acid
PDGFB	HSPA1A	both affected by valproic acid
PDGFB	CES2	both affected by propylthiouracil and valproic acid
PDGFB	CBR3	both affected by propylthiouracil and valproic acid
PDGFB	NRG1	both affected by propylthiouracil and valproic acid
PGD	FGF13	both affected by valproic acid
PGD	GPX3	both affected by valproic acid
SERPINA1	CES2	both affected by propylthiouracil and valproic acid
SERPINA1	CYP2A6	both affected by valproic acid
SERPINA1	SLC5A3	both affected by propylthiouracil and valproic acid
SERPINA1	MAFG	both affected by flutamide and valproic acid
SERPINA1	HSP90AB1	both affected by flutamide and valproic acid
SERPINA1	SLC2A10	both affected by valproic acid
SERPINA1	ABCC4	both affected by flutamide, propylthiouracil and valproic acid
SERPINA1	GCLC	both affected by azathioprine, propylthiouracil and valproic acid
SERPINA1	SLC5A6	both affected by flutamide and valproic acid
SERPINA1	CBR3	both affected by propylthiouracil and valproic acid
SERPINA1	PTGR1	both affected by flutamide, propylthiouracil and valproic acid
SERPINA1	SLC6A7	both affected by propylthiouracil
SERPINA1	TGFBR2	both affected by valproic acid
SERPINA1	SLC7A11	both affected by azathioprine, propylthiouracil and valproic acid
SERPINA1	CBR1	both affected by flutamide, propylthiouracil and valproic acid
PPARD	SLC5A11	both affected by propylthiouracil and valproic acid
RXRA	TGFB2	both affected by valproic acid
RXRA	SLC6A6	both affected by valproic acid
RXRA	SLC5A3	both affected by valproic acid
RXRA	GSTM5	both affected by valproic acid
SLC2A1	NFE2L2	both affected by valproic acid
SLC2A1	FGF13	both affected by valproic acid
SLC2A1	SLC7A11	both affected by valproic acid
SLC2A1	KEAP1	both affected by valproic acid
SLC2A1	SLC39A10	both affected by valproic acid
SLC2A2	EGR1	both affected by valproic acid
SLC2A2	MAFG	both affected by valproic acid
SLC2A2	RXRA	both affected by valproic acid
SLC2A2	ABCC4	both affected by valproic acid
SLC2A2	TGFBR2	both affected by valproic acid
SLC2A2	MAFF	both affected by valproic acid
SLC2A2	PDGFB	both affected by valproic acid

SLC2A2	SLC39A14	both affected by valproic acid
SLC2A2	GCLM	both affected by valproic acid
SLC2A2	EPHA2	both affected by valproic acid
SLC2A2	NFE2L2	both affected by valproic acid
SLC2A2	CBR1	both affected by valproic acid
SLC2A2	HBEGF	both affected by valproic acid
SLC2A3	PTGR1	both affected by carbamazepine, flutamide, propylthiouracil and valproic acid
SLC2A3	EPHA3	both affected by valproic acid
SLC2A3	TGFBR2	both affected by carbamazepine and valproic acid
SLC2A4	ABCC4	both affected by propylthiouracil and valproic acid
SLC2A4	SLC39A3	both affected by valproic acid
SLC2A5	ABCC5	both affected by valproic acid
SLC5A3	PDGFB	both affected by propylthiouracil and valproic acid
SLC5A3	EPHA2	both affected by carbamazepine and valproic acid
SLC5A3	NFE2L2	both affected by carbamazepine and valproic acid
SLC5A3	SERPINA1	both affected by propylthiouracil and valproic acid
SLC5A3	GPX2	both affected by carbamazepine and propylthiouracil
SLC5A3	HBEGF	both affected by carbamazepine, propylthiouracil and valproic acid
SLC5A3	CES2	both affected by propylthiouracil and valproic acid
SLC5A3	MAFF	both affected by carbamazepine and valproic acid
SLC5A3	CBR1	both affected by carbamazepine, propylthiouracil and valproic acid
SLC5A3	SLC39A8	both affected by propylthiouracil and valproic acid
SLC5A3	TXN	both affected by valproic acid
SLC5A5	SLC39A14	both affected by valproic acid
SLC5A5	SLC39A10	both affected by carbamazepine and valproic acid
SLC5A5	SLC39A11	both affected by valproic acid
SLC5A5	EGR1	both affected by propylthiouracil and valproic acid
SLC6A1	HSPA1A	both affected by valproic acid
SLC6A1	NRG1	both affected by valproic acid
SLC6A1	TGFB2	both affected by valproic acid
SLC6A1	ABCC5	both affected by valproic acid
SLC6A1	ME1	both affected by valproic acid
SLC6A2	MAFG	both affected by valproic acid
SLC6A4	GPX2	both affected by carbamazepine
SLC6A6	NQO1	both affected by ketoconazole and valproic acid
SLC6A6	PPARD	both affected by propylthiouracil and valproic acid
SLC6A6	GSR	both affected by valproic acid
SLC6A6	GPX2	both affected by propylthiouracil
SLC6A6	CES2	both affected by ketoconazole, propylthiouracil and valproic acid
SLC6A6	HSPA1A	both affected by valproic acid
SLC6A6	HSP90AB1	both affected by valproic acid
SLC6A6	NFE2L2	both affected by ketoconazole and valproic acid
SLC6A7	EGR1	both affected by propylthiouracil
SLC6A8	CES5A	both affected by propylthiouracil
SLC6A8	HSPA1A	both affected by carbamazepine and valproic acid
SLC6A8	HBEGF	both affected by carbamazepine, propylthiouracil and valproic acid
SLC6A8	PDGFB	both affected by propylthiouracil and valproic acid
SLC6A9	SLC39A4	both affected by propylthiouracil
SLC6A9	NFE2L2	both affected by coumarin and flutamide
SLC6A9	GCLM	both affected by coumarin and flutamide
SLC6A9	HBEGF	both affected by propylthiouracil
SLC6A11	EPHA2	both affected by valproic acid
SLC6A11	GPX2	both affected by propylthiouracil
SLC6A13	SLC39A5	both affected by valproic acid
SLC6A13	CES5A	both affected by propylthiouracil
SLC6A13	NFE2L2	both affected by coumarin, flutamide and valproic acid
SLC6A13	SLC39A9	both affected by valproic acid
SOD3	MAFG	both affected by flutamide
SOD3	SLC39A8	both affected by flutamide and propylthiouracil
SOD3	SLC5A3	both affected by propylthiouracil
TGFA	FGF13	both affected by valproic acid
TGFA	NFE2L2	both affected by valproic acid
TGFA	CES2	both affected by valproic acid
TGFA	GCLC	both affected by valproic acid
TGFA	MAFF	both affected by valproic acid
TGFB2	SLC2A2	both affected by valproic acid

TGFBR2	SLC39A14	both affected by valproic acid
TXN	SLC2A2	both affected by valproic acid
TXN	CES2	both affected by valproic acid
TXN	SLC5A8	both affected by valproic acid
TXN	NRG1	both affected by valproic acid
TXN	SLC39A6	both affected by valproic acid
TXN	PPARD	both affected by valproic acid
TXN	HBEGF	both affected by valproic acid
TXNRD1	SLC7A11	both affected by carbamazepine and valproic acid
TXNRD1	HBEGF	both affected by carbamazepine and valproic acid
SLC39A7	CBR1	both affected by valproic acid
ABCC3	PDGFB	both affected by propylthiouracil and valproic acid
ABCC3	SLC5A3	both affected by carbamazepine, propylthiouracil and valproic acid
ABCC3	CBR1	both affected by carbamazepine, coumarin, flutamide, propylthiouracil and valproic acid
ABCC3	HSPA1A	both affected by carbamazepine and valproic acid
ABCC3	SLC2A9	both affected by propylthiouracil and valproic acid
ABCC3	GSTM5	both affected by coumarin and valproic acid
ABCC3	FGF13	both affected by propylthiouracil and valproic acid
CES2	NFE2L2	both affected by ketoconazole and valproic acid
CES2	GCLM	both affected by valproic acid
CES2	ALDH3A1	both affected by propylthiouracil
CES2	SERPINA1	both affected by propylthiouracil and valproic acid
CES2	SLC39A11	both affected by valproic acid
CES2	TXN	both affected by valproic acid
CES2	EPHA2	both affected by valproic acid
CES2	TGFA	both affected by valproic acid
CES2	PDGFB	both affected by propylthiouracil and valproic acid
SQSTM1	ME1	both affected by carbamazepine, flutamide and valproic acid
SQSTM1	SLC2A10	both affected by valproic acid
SLC5A6	GSTA3	both affected by flutamide and valproic acid
SLC5A6	TGFB2	both affected by valproic acid
SLC5A6	GPX2	both affected by flutamide
SLC5A6	HSP90AB1	both affected by flutamide and valproic acid
KEAP1	CBR3	both affected by valproic acid
KEAP1	GCLM	both affected by azathioprine, flutamide and valproic acid
ABCC5	HBEGF	both affected by valproic acid
ABCC4	HBEGF	both affected by carbamazepine, propylthiouracil and valproic acid
SLC2A6	NFE2L2	both affected by valproic acid
SLC2A6	MGST3	both affected by valproic acid
SLC2A6	GSTA1	both affected by valproic acid
SLC2A6	EPHA2	both affected by valproic acid
SLC2A6	CBR1	both affected by valproic acid
SLC2A6	SLC39A5	both affected by valproic acid
SLC2A6	TXN	both affected by valproic acid
SLC2A6	GSTM5	both affected by valproic acid
SLC2A6	FTH1	both affected by valproic acid
SLC2A6	HSP90AB1	both affected by valproic acid
PTGR1	CES5A	both affected by propylthiouracil
PTGR1	SOD3	both affected by flutamide and propylthiouracil
PTGR1	SLC39A2	both affected by carbamazepine
PTGR1	ALDH3A1	both affected by propylthiouracil
PTGR1	SLC6A6	both affected by propylthiouracil and valproic acid
PTGR1	SLC39A11	both affected by valproic acid
PTGR1	SLC39A10	both affected by carbamazepine and valproic acid
PTGR1	GPX2	both affected by carbamazepine, flutamide and propylthiouracil
PTGR1	MAFF	both affected by carbamazepine and valproic acid
PTGR1	UGT1A6	both affected by carbamazepine, flutamide and valproic acid
PTGR1	SLC2A1	both affected by valproic acid
PTGR1	GSTA1	both affected by carbamazepine and valproic acid
PTGR1	HSPA1A	both affected by carbamazepine and valproic acid
SLC39A14	NFE2L2	both affected by valproic acid
SLC39A14	ABCC3	both affected by valproic acid
SLC39A14	SLC2A2	both affected by valproic acid
SLC39A14	SLC2A10	both affected by valproic acid
SLC39A14	MAFG	both affected by valproic acid
SLC39A14	CES2	both affected by valproic acid

SLC39A14	EGR1	both affected by valproic acid
SLC7A11	GSTA1	both affected by azathioprine, carbamazepine and valproic acid
SLC7A11	SLC6A6	both affected by propylthiouracil and valproic acid
SLC7A11	DNAJB1	both affected by carbamazepine
SLC7A11	SQSTM1	both affected by azathioprine, carbamazepine and valproic acid
SLC7A11	CBR3	both affected by propylthiouracil and valproic acid
SLC7A11	SLC39A3	both affected by valproic acid
SLC7A11	GSTM5	both affected by valproic acid
SLC7A11	SLC2A10	both affected by valproic acid
SLC7A11	MAFF	both affected by carbamazepine and valproic acid
SLC7A11	ABCC3	both affected by carbamazepine, propylthiouracil and valproic acid
MAFF	TGFA	both affected by valproic acid
MAFF	UGT1A6	both affected by carbamazepine and valproic acid
MAFF	CBR3	both affected by valproic acid
SLC39A6	CBR3	both affected by propylthiouracil and valproic acid
SLC39A6	SLC2A2	both affected by valproic acid
SLC39A6	GSTM5	both affected by valproic acid
SLC39A6	TXN	both affected by valproic acid
SLC39A6	ABCC3	both affected by flutamide, propylthiouracil and valproic acid
SLC6A16	CES2	both affected by valproic acid
SLC6A16	CBR1	both affected by carbamazepine and valproic acid
SLC39A3	SLC2A10	both affected by valproic acid
SLC39A3	MAFG	both affected by valproic acid
SLC39A3	G6PD	both affected by valproic acid
SLC39A3	FTH1	both affected by valproic acid
SLC39A2	SLC5A3	both affected by carbamazepine
SLC2A8	CBR1	both affected by valproic acid
SLC2A8	PDGFB	both affected by valproic acid
SLC2A8	MAFG	both affected by valproic acid
SLC2A8	SLC39A10	both affected by valproic acid
SLC2A8	SLC39A11	both affected by valproic acid
UGT1A6	SLC2A1	both affected by valproic acid
SLC6A20	EGR1	both affected by propylthiouracil and valproic acid
SLC6A20	GPX2	both affected by propylthiouracil
SLC6A20	RXRA	both affected by valproic acid
SLC6A15	HBEGF	both affected by valproic acid
SLC6A15	HSPA1A	both affected by valproic acid
SLC6A15	TXN	both affected by valproic acid
SLC39A9	EPHA3	both affected by valproic acid
SLC39A4	CBR1	both affected by propylthiouracil
SLC2A9	GCLC	both affected by valproic acid
SLC2A9	FTH1	both affected by valproic acid
SLC2A9	ABCC3	both affected by propylthiouracil and valproic acid
SLC39A10	HBEGF	both affected by carbamazepine and valproic acid
SLC39A10	SLC2A10	both affected by valproic acid
SLC39A10	GCLC	both affected by carbamazepine and valproic acid
SLC39A10	CES2	both affected by valproic acid
SLC39A10	GCLM	both affected by carbamazepine and valproic acid
SLC39A10	CBR1	both affected by carbamazepine and valproic acid
SLC5A7	HSP90AB1	both affected by valproic acid
SLC39A8	EPHA3	both affected by valproic acid
SLC39A8	NFE2L2	both affected by flutamide and valproic acid
SLC39A8	SLC5A3	both affected by propylthiouracil and valproic acid
SLC39A8	SLC6A7	both affected by propylthiouracil
SLC39A8	GPX2	both affected by flutamide and propylthiouracil
SLC39A8	GSTA1	both affected by valproic acid
SLC39A8	SLC2A10	both affected by valproic acid
SLC39A8	SOD3	both affected by flutamide and propylthiouracil
SLC39A8	GSTM5	both affected by valproic acid
SLC2A10	CBR1	both affected by valproic acid
SLC2A10	GSTA3	both affected by valproic acid
SLC2A10	GCLM	both affected by valproic acid
SLC2A10	SLC39A11	both affected by valproic acid
TXNRD3	SLC39A14	both affected by valproic acid
TXNRD3	CES5A	both affected by propylthiouracil
TXNRD3	PPARD	both affected by propylthiouracil and valproic acid

TXNRD3	HBEGF	both affected by propylthiouracil and valproic acid
TXNRD3	SLC2A3	both affected by flutamide, propylthiouracil and valproic acid
TXNRD3	ABCC5	both affected by flutamide and valproic acid
SLC2A13	KEAP1	both affected by valproic acid
SLC2A13	PDGFB	both affected by valproic acid
SLC2A13	SLC39A10	both affected by valproic acid
SLC2A13	SLC39A6	both affected by valproic acid
SLC2A13	HSPA1A	both affected by valproic acid
SLC2A13	EGR1	both affected by valproic acid
SLC5A11	TGFBR2	both affected by valproic acid
SLC5A11	RXRA	both affected by valproic acid
SLC5A11	GSTM5	both affected by valproic acid
SLC5A11	HSPA1A	both affected by valproic acid
SLC5A11	SLC39A5	both affected by valproic acid
SLC5A11	EGR1	both affected by propylthiouracil and valproic acid
SLC5A11	ABCC4	both affected by propylthiouracil and valproic acid
SLC5A11	ABCC5	both affected by propylthiouracil and valproic acid
SLC5A11	CBR1	both affected by propylthiouracil and valproic acid
SLC5A11	CYP2A6	both affected by valproic acid
SLC5A11	ABCC3	both affected by propylthiouracil and valproic acid
SLC2A12	TGFBR2	both affected by valproic acid
SLC2A12	GCLC	both affected by valproic acid
SLC2A12	SLC39A10	both affected by valproic acid
SLC2A12	ABCC4	both affected by propylthiouracil and valproic acid
SLC2A12	FTH1	both affected by valproic acid
SLC2A12	EGR1	both affected by propylthiouracil and valproic acid
SLC5A12	CBR3	both affected by propylthiouracil and valproic acid
SLC5A12	GSTM5	both affected by valproic acid
SLC5A12	EGR1	both affected by propylthiouracil and valproic acid
SLC5A12	ABCC3	both affected by propylthiouracil and valproic acid
SLC5A8	ABCC5	both affected by valproic acid
SLC5A8	ABCC3	both affected by valproic acid
SLC5A8	EGR1	both affected by valproic acid
SLC39A11	EGR1	both affected by valproic acid
SLC39A11	SLC6A11	both affected by valproic acid
SLC39A11	EPHA2	both affected by valproic acid
SLC39A11	SLC5A6	both affected by valproic acid
SLC39A11	CBR3	both affected by valproic acid
SLC39A11	GSTM5	both affected by valproic acid
SLC39A11	CES2	both affected by valproic acid
CES5A	SLC6A8	both affected by propylthiouracil
CES5A	EGR1	both affected by propylthiouracil
CES5A	HBEGF	both affected by propylthiouracil
CES5A	ABCC3	both affected by propylthiouracil
SLC39A5	NFE2L2	both affected by valproic acid
SLC39A5	SLC6A15	both affected by valproic acid
SLC39A5	HBEGF	both affected by valproic acid
SLC39A5	SLC5A3	both affected by valproic acid
SLC39A5	MAFG	both affected by valproic acid
SLC6A18	HSPA1A	both affected by valproic acid

Table S5-8: Unknown interactions in CPDB – evidence for a relationship from other databases – summary of Tables S5-5, S5-6, S5-7, S5-8. Interactions for which no evidence could be found were omitted. MF = molecular function, BP = biological process, CC = cellular component.

from	to	Biograph	STRING	CTD gene pair - compound
BLVRB	GSTP1			x
CBR1	GPX2			x
CBR3	SLC39A10			x
CBR3	SLC6A20			x
CBR3	ABCC2			x
CBR3	PPARD	x		x
CBR3	NRG1			x
CBR3	GSTM3	x		x
CBR3	SLC39A11			x
CBR3	NQO1	x		x
CBR3	SLC5A3			x
CBR3	SERPINA1	x		x
CBR3	GCLM			x
CBR3	PDGFB	x		x
ABCC2	CES2			x
ABCC2	PPARD			x
ABCC2	SLC6A6			x
ABCC2	CBR3			x
CYP2A6	SLC2A10			x
CYP2A6	SLC2A1			x
CYP2A6	SLC39A10			x
CYP2A6	SLC6A6			x
CBR3	GCLM			x
CYP2A6	GPX2	x		x
CYP4A11	HSPA1A	x		
CYP4A11	SLC2A1	x		
NQO1	SLC2A10			x
EGR1	SLC39A6			x
EGR1	PTGR1			x
EGR1	SLC39A5			x
EPHA2	GCLM			x
EPHA2	GPX2			x
EPHA2	FGF13	x	x	x
EPHA2	SLC6A6			x
EPHA2	SLC39A11			x
FGF13	SLC5A11			x
FGF13	ABCC3			x
FGF13	SLC2A1			x
FTH1	CBR1			x
FTH1	SLC2A10			x
FTH1	SLC6A1			x
FTL	SLC5A3			x
FTL	EPHA3	x		x
FTL	HBEGF			x
FTL	SLC6A7			x
FTL	SLC6A15			x
FTL	SLC7A11			x
G6PD	MGST2			x
G6PD	CBR1	x		x
G6PD	FGF13			x
G6PD	TXNRD1	x	x	x
G6PD	FTL			x
G6PD	SLC2A2			x
G6PD	KEAP1			x
G6PD	SLC5A6			x
G6PD	SLC39A11			x
GCLC	DNAJB1			x
GCLC	SLC2A2			x
GCLC	SLC7A11		x	x
GCLM	SLC6A20			x
GCLM	GSTA4		x	x

from	to	Biograph	STRING	CTD gene pair – compound
GCLM	SLC39A10			x
GCLM	CBR3			x
GCLM	GSTM5			x
GCLM	PRDX6			x
GCLM	ABCC5		x	x
GCLM	GSTA1		x	x
GCLM	SLC2A12			x
GCLM	SLC7A11		x	x
GCLM	DNAJB1			x
GPX2	SLC7A11			x
GPX2	EPHA2			x
GPX2	SLC6A6			x
GPX2	ADH7	x		
GPX2	TGFBR2			x
GPX2	DNAJB1			x
GPX2	HSP90AA1			x
GPX2	SLC39A8			x
GPX2	SLC5A3			x
GPX2	CBR1			x
GPX2	HSP90AB1			x
GPX2	SLC5A6			x
GPX2	ABCC4			x
GPX3	SLC5A6			x
GPX3	HSPA1A			x
GPX3	SLC2A1			x
GSR	SLC2A1			x
GSTA1	GCLM		x	x
GSTA1	HSPA1A			x
GSTA1	SLC2A1			x
GSTA4	PDGFB			x
GSTM3	NFE2L2	x		x
GSTM3	HBEGF	x		x
GSTM3	CBR3			x
GSTM3	SLC2A13			x
GSTM3	EGR1	x		x
GSTM5	ABCC5			x
GSTM5	EGR1			x
GSTM5	ABCC3			x
GSTM5	SLC39A5			x
GSTM5	CBR1			x
GSTM5	SLC5A11			x
GSTM5	HSPA1A			x
GSTM5	SLC5A12			x
GSTM5	SLC7A11			x
GSTP1	SLC39A11			x
GSTP1	MAFF			x
NRG1	TXN	x		x
NRG1	PDGFB	x		x
NRG1	SLC6A6			x
NRG1	MAFG			x
NRG1	CBR3			x
HMOX1	SLC6A6			x
HMOX1	SLC39A2			x
HMOX1	SLC39A10			x
HMOX1	SLC39A11			x
HMOX1	GSTM5	x		x
HMOX1	GPX3	x	x	x
HMOX1	GSTA4	x		x
HMOX1	SLC39A14			x
HSPA1A	MAFG	x		x
HMOX1	SLC2A10			x
HSPA1A	CES2			x
HSPA1A	SLC39A10			x
HSPA1A	SLC5A6			x

from	to	Biograph	STRING	CTD gene pair – compound
HSPA1A	ABCC3			x
HSPA1A	TGFB2			x
HSP90AA1	SLC39A10			x
HSP90AA1	GPX2			x
HSP90AB1	SLC6A9	x		x
MAFG	SLC6A3	x		
MAFG	SLC6A20			x
HSP90AA1	SLC39A10			x
MAFG	PPARD	x		x
MAFG	PDGFB			x
MAFG	SLC6A1	x		x
MAFG	SLC39A7	x		x
MAFG	SERPINA1	x		x
MAFG	SLC39A5			x
MAFG	SLC7A11			x
MAFG	HSPA1A	x		x
MAFG	SLC6A4	x		
MAFG	ME1			x
MAFG	NRG1			x
ME1	SLC39A5			x
ME1	HSPA1A			x
ME1	MAFG			x
ME1	SLC6A1			x
ME1	NFE2L2			x
MGST2	SLC6A20			x
MGST3	SLC39A6			x
MGST3	ABCC5			x
NFE2L2	SLC39A2			x
NFE2L2	SLC39A8			x
NFE2L2	ABCC5			x
NFE2L2	PDGFB			x
NFE2L2	TGFA			x
NFE2L2	SLC2A2			x
NFE2L2	CES2			x
NFE2L2	SLC5A3			x
NFE2L2	HBEGF			x
PRDX1	GSTM5	x		x
PRDX1	SLC6A15			x
PRDX1	ABCC5			x
PDGFB	MAFG			x
PDGFB	HSPA1A			x
PDGFB	CES2			x
PDGFB	CBR3	x		x
PDGFB	NRG1	x		x
PGD	FGF13			x
PGD	GPX3			x
SERPINA1	CES2			x
SERPINA1	CYP2A6			x
SERPINA1	SLC5A3			x
SERPINA1	MAFG	x		x
SERPINA1	HSP90AB1	x		x
SERPINA1	SLC2A10			x
SERPINA1	ABCC4			x
SERPINA1	GCLC			x
SERPINA1	SLC5A6			x
SERPINA1	CBR3	x		x
SERPINA1	PTGR1			x
SERPINA1	SLC6A7			x
SERPINA1	TGFBR2	x		x
SERPINA1	SLC7A11			x
SERPINA1	CBR1	x		x
PPARD	SLC5A11			x
RXRA	TGFB2			x
RXRA	SLC6A6			x

from	to	Biograph	STRING	CTD gene pair – compound
RXRA	SLC5A3			x
RXRA	GSTM5			x
SLC2A1	NFE2L2			x
SLC2A1	FGF13			x
SLC2A1	SLC7A11			x
SLC2A1	KEAP1	x		x
SLC2A1	SLC39A10			x
SLC2A2	EGR1			x
SLC2A2	MAFG	x		x
SLC2A2	RXRA			x
SLC2A2	ABCC4			x
SLC2A2	TGFBR2			x
SLC2A2	MAFF			x
SLC2A2	PDGFB			x
SLC2A2	SLC39A14			x
SLC2A2	GCLM			x
SLC2A2	EPHA2		x	x
SLC2A2	NFE2L2			x
SLC2A2	CBR1	x		x
SLC2A2	HBEGF			x
SLC2A3	PTGR1			x
SLC2A3	EPHA3			x
SLC2A3	TGFBR2			x
SLC2A4	ABCC4			x
SLC2A4	SLC39A3			x
SLC2A5	ABCC5			x
SLC5A1	TGFB2	x		
SLC5A3	PDGFB			x
SLC5A3	EPHA2			x
SLC5A3	NFE2L2			x
SLC5A3	SERPINA1			x
SLC5A3	GPX2			x
SLC5A3	HBEGF			x
SLC5A3	CES2			x
SLC5A3	MAFF			x
SLC5A3	CBR1			x
SLC5A3	SLC39A8			x
SLC5A3	TXN			x
SLC5A5	SLC39A14			x
SLC5A5	SLC39A10			x
SLC5A5	SLC39A11			x
SLC5A5	EGR1			x
SLC6A1	HSPA1A			x
SLC6A1	NRG1			x
SLC6A1	TGFB2			x
SLC6A1	ABCC5			x
SLC6A1	ME1			x
SLC6A2	MAFG	x		x
SLC6A4	GPX2			x
SLC6A6	NQO1			x
SLC6A6	PPARD			x
SLC6A6	GSR			x
SLC6A6	GPX2			x
SLC6A6	CES2			x
SLC6A6	HSPA1A			x
SLC6A6	HSP90AB1			x
SLC6A6	NFE2L2			x
SLC6A7	EGR1			x
SLC6A8	CES5A			x
SLC6A8	HSPA1A	x		x
SLC6A8	HBEGF	x		x
SLC6A8	PDGFB			x
SLC6A9	SLC39A4			x
SLC6A9	NFE2L2	x		x

from	to	Biograph	STRING	CTD gene pair – compound
SLC6A9	HSPA1A	x		
SLC6A9	GCLM			x
SLC6A9	HBEGF	x		x
SLC6A11	EPHA2			x
SLC6A11	GPX2			x
SLC6A13	SLC39A5			x
SLC6A13	CES5A			x
SLC6A13	NFE2L2			x
SLC6A13	SLC39A9			x
SOD3	MAFG			x
SOD3	SLC39A8			x
SOD3	SLC5A3			x
TGFA	FGF13			x
TGFA	NFE2L2			x
TGFA	CES2			x
TGFA	GCLC			x
TGFA	MAFF			x
TGFB2	SLC2A2	x		x
TGFBR2	SLC39A14			x
TXN	SLC2A2	x		x
TXN	CES2			x
TXN	SLC5A8			x
TXN	NRG1	x		x
TXN	SLC39A6			x
TXN	PPARD	x		x
TXN	HBEGF	x		x
TXNRD1	SLC7A11			x
TXNRD1	HBEGF	x		x
SLC39A7	CBR1	x		x
ABCC3	PDGFB			x
ABCC3	SLC5A3			x
ABCC3	CBR1			x
ABCC3	HSPA1A			x
ABCC3	SLC2A9			x
ABCC3	GSTM5			x
ABCC3	FGF13			x
CES2	NFE2L2			x
CES2	GCLM			x
CES2	ALDH3A1	x		x
CES2	SERPINA1			x
CES2	SLC39A11			x
CES2	TXN			x
CES2	EPHA2			x
CES2	TGFA			x
CES2	PDGFB			x
SQSTM1	ME1			x
SQSTM1	SLC2A10			x
SLC5A6	GSTA3			x
SLC5A6	TGFB2			x
SLC5A6	GPX2			x
SLC5A6	HSP90AB1			x
KEAP1	CBR3	x		x
KEAP1	GCLM	x	x	x
ABCC5	HBEGF			x
ABCC4	HBEGF			x
SLC2A6	NFE2L2			x
SLC2A6	MGST3			x
SLC2A6	GSTA1			x
SLC2A6	EPHA2			x
SLC2A6	CBR1			x
SLC2A6	SLC39A5			x
SLC2A6	TXN			x
SLC2A6	GSTM5			x
SLC2A6	FTH1			x

from	to	Biograph	STRING	CTD gene pair – compound
SLC2A6	HSP90AB1			x
PTGR1	CES5A			x
PTGR1	SOD3			x
PTGR1	SLC39A2			x
PTGR1	ALDH3A1	x		x
PTGR1	SLC6A6			x
PTGR1	SLC39A11			x
PTGR1	SLC39A10			x
PTGR1	GPX2			x
PTGR1	MAFF			x
PTGR1	UGT1A6			x
PTGR1	SLC2A1	x		x
PTGR1	GSTA1			x
PTGR1	HSPA1A	x		x
SLC39A14	NFE2L2			x
SLC39A14	ABCC3			x
SLC39A14	SLC2A2			x
SLC39A14	SLC2A10			x
SLC39A14	MAFG			x
SLC39A14	CES2			x
SLC39A14	EGR1			x
SLC7A11	GSTA1			x
SLC7A11	SLC6A6		x	x
SLC7A11	DNAJB1			x
SLC7A11	SQSTM1			x
SLC7A11	CBR3			x
SLC7A11	SLC39A3			x
SLC7A11	GSTM5			x
SLC7A11	SLC2A10			x
SLC7A11	MAFF			x
SLC7A11	ABCC3			x
MAFF	TGFA			x
MAFF	UGT1A6			x
MAFF	CBR3	x		x
SLC39A6	CBR3			x
SLC39A6	SLC2A2			x
SLC39A6	GSTM5			x
SLC39A6	TXN			x
SLC39A6	ABCC3			x
SLC6A16	CES2			x
SLC6A16	CBR1			x
SLC39A3	SLC2A10			x
SLC39A3	MAFG			x
SLC39A3	G6PD			x
SLC39A3	FTH1			x
SLC39A2	SLC5A3			x
SLC2A8	CBR1			x
SLC2A8	PDGFB			x
SLC2A8	MAFG			x
SLC2A8	SLC39A10			x
SLC2A8	SLC39A11			x
UGT1A6	SLC2A1			x
SLC6A20	EGR1			x
SLC6A20	GPX2			x
SLC6A20	RXRA			x
SLC6A15	HBEGF			x
SLC6A15	HSPA1A			x
SLC6A15	TXN			x
SLC39A9	EPHA3			x
SLC39A4	CBR1			x
SLC2A9	GCLC			x
SLC2A9	FTH1			x
SLC2A9	ABCC3			x
SLC39A10	HBEGF			x

from	to	Biograph	STRING	CTD gene pair – compound
SLC39A10	SLC2A10			x
SLC39A10	GCLC			x
SLC39A10	CES2			x
SLC39A10	GCLM			x
SLC39A10	CBR1			x
SLC5A7	HSP90AB1	x		x
SLC39A8	EPHA3			x
SLC39A8	NFE2L2			x
SLC39A8	SLC5A3			x
SLC39A8	SLC6A7			x
SLC39A8	GPX2			x
SLC39A8	GSTA1			x
SLC39A8	SLC2A10			x
SLC39A8	SOD3			x
SLC39A8	GSTM5			x
SLC2A10	CBR1			x
SLC2A10	GSTA3			x
SLC2A10	GCLM			x
SLC2A10	SLC39A11			x
TXNRD3	SLC39A14			x
TXNRD3	CES5A			x
TXNRD3	PPARD			x
TXNRD3	HBEGF			x
TXNRD3	SLC2A3			x
TXNRD3	ABCC5		x	x
SLC2A13	KEAP1			x
SLC2A13	PDGFB			x
SLC2A13	SLC39A10			x
SLC2A13	SLC39A6			x
SLC2A13	HSPA1A			x
SLC2A13	EGR1			x
SLC5A11	TGFBR2			x
SLC5A11	RXRA			x
SLC5A11	GSTM5			x
SLC5A11	HSPA1A			x
SLC5A11	SLC39A5			x
SLC5A11	EGR1			x
SLC5A11	ABCC4			x
SLC5A11	ABCC5			x
SLC5A11	CBR1			x
SLC5A11	CYP2A6			x
SLC5A11	ABCC3			x
SLC2A12	TGFBR2			x
SLC2A12	GCLC			x
SLC2A12	SLC39A10			x
SLC2A12	ABCC4			x
SLC2A12	FTH1			x
SLC2A12	EGR1			x
SLC5A12	CBR3			x
SLC5A12	GSTM5			x
SLC5A12	EGR1			x
SLC5A12	ABCC3			x
SLC5A8	ABCC5			x
SLC5A8	ABCC3			x
SLC5A8	EGR1			x
SLC39A11	EGR1			x
SLC39A11	SLC6A11			x
SLC39A11	EPHA2			x
SLC39A11	SLC5A6			x
SLC39A11	CBR3			x
SLC39A11	GSTM5			x
SLC39A11	CES2			x
CES5A	SLC6A8			x
CES5A	EGR1			x

from	to	Biograph	STRING	CTD gene pair – compound
CES5A	HBEGF			x
CES5A	ABCC3			x
SLC39A5	NFE2L2			x
SLC39A5	SLC6A15			x
SLC39A5	HBEGF			x
SLC39A5	SLC5A3			x
SLC39A5	MAFG			x
SLC6A18	HSPA1A			x

Table S5-9: True positive edges in Figure S5-1 that could also be inferred when analysing 10 compounds not affecting the NRF2 pathway.

from	to	interaction	intermediate(s)	involved in which pathway(s)?
SLC6A6	SLC5A3	indirect	Na+	transport of taurine (Humancyc)<br>sodium coupled myo-inositol plasma membrane transport (Humancyc)<br>Inositol transporters (Reactome)
GSTP1	MGST3	direct		Metabolism of xenobiotics by cytochrome P450 (KEGG)
CBR1	NFE2L2	indirect	AK1C1	Metabolism of xenobiotics by cytochrome P450 (KEGG)
SLC6A2	SLC5A3	indirect	Na+	Na+/Cl- dependent neurotransmitter transporters (Reactome)<br>sodium coupled noradrenaline plasma membrane transport (Humancyc)<br>sodium coupled myo-inositol plasma membrane transport (Humancyc)<br>Inositol transporters (Reactome)
SOD3	TGFBR2	indirect	SP1	Not assigned to a pathway
NFE2L2	CBR3	indirect	AKR1C1	Metabolism of xenobiotics by cytochrome P450 (KEGG)
SLC2A8	MAFF	indirect	MAX	Not assigned to a pathway
SLC39A10	EGR1	indirect	CREB1	Not assigned to a pathway
NFE2L2	PRDX6	indirect	PRDX1<br>GPX2<br><br>RARA	Not assigned to a pathway<br>Synthesis of 5-eicosatetraenoic acids (Reactome)<br>Arachidonic acid metabolism (KEGG)<br>Not assigned to a pathway

Table S5-10: Unknown edges in Figure S5-1 that could also be inferred when analysing 10 compounds not affecting the NRF2 pathway.

from	to	interaction in Biograph?
SLC2A2	MAFG	yes
SLC2A2	TGFBR2	no
SLC2A2	MAFF	no
SLC5A10	TXN	no
GSTM5	SLC39A5	no
SLC39A12	NFE2L2	no
NFE2L2	SLC2A2	no
GSTP1	MAFF	no
CES5A	MGST3	no
ABCC5	SLC39A12	no
ABCC3	SLC5A10	no
SLC6A9	NFE2L2	yes
SLC39A3	FTH1	no
TGFA	GCLC	no
HMOX1	SLC39A14	no
SLC2A2	NFE2L2	no
HMOX1	SLC2A10	no
SOD3	SLC5A3	no
TXN	HBEGF	yes
GSTM3	EGR1	yes
CYP4A11	SLC5A3	no
SLC39A11	GSTM5	no
SLC7A11	ABCC3	no

References

Bhat R, Axtell R, Mitra A, et al. (2010) Inhibitory role for GABA in autoimmune inflammation. PNAS 107(6):2580-5

Grotz MR, Pape HC, van Griensven M, et al. (2001) Glycine reduces the inflammatory response and organ damage in a two-hit sepsis model in rats. Shock 16(2):116-21

Lemmon MA, Schlessinger J (2010) Cell signaling by receptor tyrosine kinases. Cell 141(7):1117-34

Mayer MP, Bukau B (2005) Hsp70 chaperones: cellular functions and molecular mechanism. Cellular and molecular life sciences : CMLS 62(6):670-84

Wang W, Shang LH, Jacobs DO (2002) Complement regulatory protein CD59 involves c-SRC related tyrosine phosphorylation of the creatine transporter in skeletal muscle during sepsis. Surgery 132(2):334-40
